# New Antibacterial Diterpenoids from the South China Sea Soft Coral *Klyxum molle*

**DOI:** 10.3390/md21060362

**Published:** 2023-06-16

**Authors:** Jia-Dong Yu, Dan-Dan Yu, Ming-Zhi Su, Yu-Cheng Gu, Hong Wang, Yue-Wei Guo

**Affiliations:** 1 College of Pharmaceutical Science and Collaborative Innovation Center of Yangtze River Delta Region Green Pharmaceuticals, Zhejiang University of Technology, Hangzhou 310014, China; yjd0727@outlook.com; 2 State Key Laboratory of Drug Research, Shanghai Institute of Materia Medica, Chinese Academy of Sciences, Shanghai 201203, China; 3 Shandong Laboratory of Yantai Drug Discovery, Bohai Rim Advanced Research Institute for Drug Discovery, Yantai 264117, China; ddyu@baridd.ac.cn (D.-D.Y.); smz0310@163.com (M.-Z.S.); 4 Syngenta, Jealott’s Hill International Research Centre, Bracknell RG42 6EY, Berkshire, UK; yucheng.gu@syngenta.com

**Keywords:** soft coral, *Klyxum molle*, diterpenoids, stereochemistry, antibacterial activity

## Abstract

Fifteen new diterpenoids, namely xishaklyanes A-O (**1**–**15**), along with three known related ones (**16**–**18**), were isolated from the soft coral *Klyxum molle* collected from Xisha Islands, South China Sea. The stereochemistry of the new compounds was elucidated by a combination of detailed spectroscopic analyses, chemical derivatization, quantum chemical calculations, and comparison with the reported data. The absolute configuration of compound **18** was established by the modified Mosher’s method for the first time. In bioassay, some of these compounds exhibited considerable antibacterial activities on fish pathogenic bacteria, and compound **4** showed the most effective activity with MIC of 0.225 μg/mL against *Lactococcus garvieae*.

## 1. Introduction

Soft corals of the genus *Klyxum* (order Alcyonacea, family Alcyoniidae) are broadly distributed over the tropical Indo-Pacific, including the South China Sea [1]. Different from the commonly chemically investigated *Sinularia* [2] and *Sarcophyton* [3] soft corals, only three species from the genus *Klyxum* have been chemically studied, including *Klyxum simplex* [4,5,6,7], *Klyxum molle* [8,9,10,11], and *Klyxum flaccidum* [12,13,14,15]. Diverse secondary metabolites were discovered, including novel skeleton (Klyflaccilides A and B) and eunicellin-type diterpenoids [8,9,10,11,12]. These compounds exhibited widespread biological activities, such as antibacterial, cytotoxic, and anti-inflammatory effects [4,5,6,7,8,9,10,11,12,14,15].

**Figure 1:**
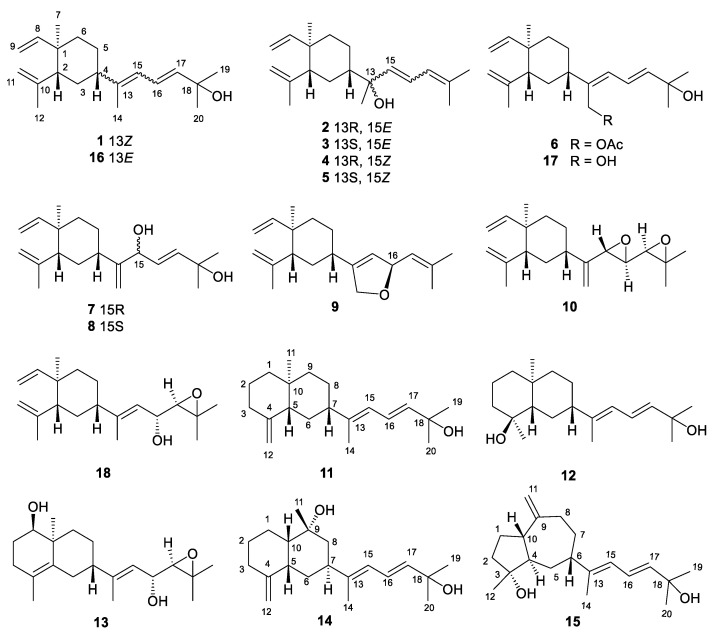
Chemical structures of the compounds isolated from *Klyxum molle*.

As part of our continuous research project aiming for the discovery of bioactive metabolites from Chinese marine *Cnidaria* [12,16,17,18], *Klyxum molle* collected off the Xisha Islands were subjected to systematic research, yielding fifteen new diterpenoids **1**−**15** and three known related diterpenoids **16**−**18** (Figure 1). Herein, the isolation, structural elucidation, and biological evaluations of these compounds are reported.

## 2. Results and Discussion

By a series of column chromatography in combination with HPLC, the acetone extract of *K. molle* resulted in the purification of fifteen new diterpenoids, namely xishaklyanes A-O (**1–15**), along with three known related ones (**16**–**18**) (Figure 1). Those known diterpenoids were unambiguously identified as fuscol (**16**) [19], lobovarol H (**17**) [20], and 17,18-epoxyloba-8,10,13(15)-trien-16-ol (**18**) [21], respectively, by comparing their NMR data and specific rotation values with those reported in the literature. 

**Figure 2:**
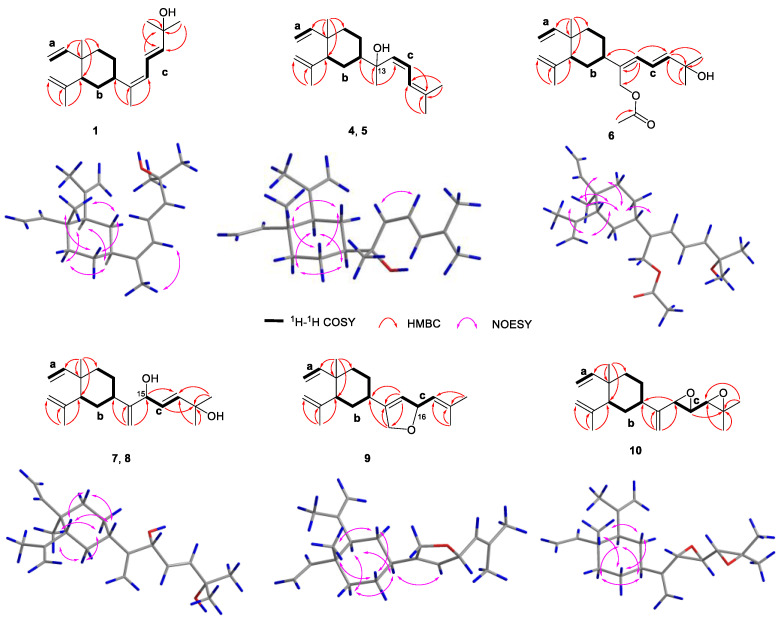
^1^H-^1^H COSY, key HMBC, and NOESY correlations of compounds **1**, **4–10**.

Xishaklyane A (**1**) was obtained as an optically active colourless oil. From the pseudo molecular ion peak at *m/z* 289.2527 ([M + H]^+^, calcd. 289.2526) in the HRESIMS spectrum, a molecular formula of C_20_H_32_O was established, indicating five degrees of unsaturation. The ^1^H and ^13^C NMR data of **1** (Table 1 and Table 2) highly resembled those of co-occurring **16**, with the only differences in signals on C-4 (*δ*_C_ 47.5 in **16** and 40.5 in **1**), C-14 (*δ*_C_ 15.3 in **16** and 20.2 in **1**), and C-15 (*δ*_C_ 122.9 in **16** and 124.6 in **1**), suggesting that **1** was the analogue of **16**, with the opposite geometry on Δ^13,15^. The characteristic NOE correlation between H_3_-14 and H-15 also confirmed the *Z* geometry of Δ^13,15^ in **1** (Figure 2). The structure of **1** was further confirmed by 2D NMR analysis, including HMBC and NOESY correlations. Thus, compound **1** was determined to be 13*Z*-fuscol, namely xishaklyane A. 

**Figure 3:**
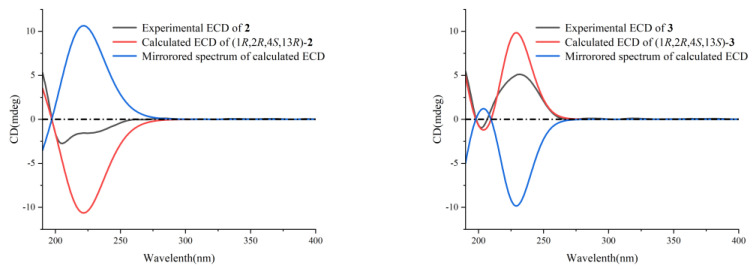

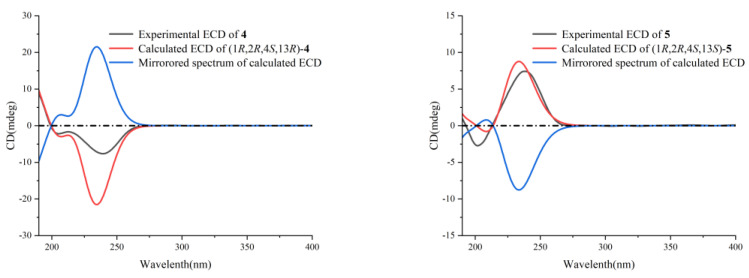
Comparison of experimental ECD spectrum (black) and DFT-predicted ECD spectra of **2–5**. ECD spectra were predicted by means of time-dependent DFT calculations at the MPW1PW91/6-31G(d, p) level.

Xishaklyane B (**2**) and xishaklyane C (**3**) were initially isolated as a mixture [22], displaying two sets of carbon signals in the ^13^C NMR spectrum. Normal Phase-High Performance Liquid Chromatography (NP-HPLC) CHIRALPAK^®^ IC (250 mm × 4.6 mm, 5 µm, Daicel Corporation, Osaka, Japan) (n-hexane/isopropanol (99.7:0.3), 1.0 mL/min) (**2**: *t*_R_ = 4.6 min; **3**: *t*_R_ = 5.5 min) was used to successfully separate the mixture into **2** and **3**. The absolute configurations (ACs) of **2** and **3** were further established by TDDFT-ECD calculations, a reliable approach to determine the ACs of natural products with chiral carbons near the chromophore groups [23,24,25,26]. As shown in Figure 3, the Boltzmann-averaged ECD spectrum of (1*R*,2*R*,4*S*,13*R*,15*E*)-**2** matched to the experimental ECD spectrum of **2**, and the Boltzmann-averaged ECD spectrum of (1*R*,2*R*,4*S*,13*S*,15*E*)-**3** matched to the experimental ECD spectrum of **3**. Consequently, the ACs of **2** and **3** were determined to be 1*R*, 2*R*, 4*S*, 13*R*, 15*E*, and 1*R*, 2*R*, 4*S*, 13*S*, 15*E*, respectively. Thus, xishaklyanes B (**2**) and C (**3**) were deduced to be 13*R*, 15*E*-isofuscol and 13*S*, 15*E*-isofusol, respectively. 

**Figure 4:**
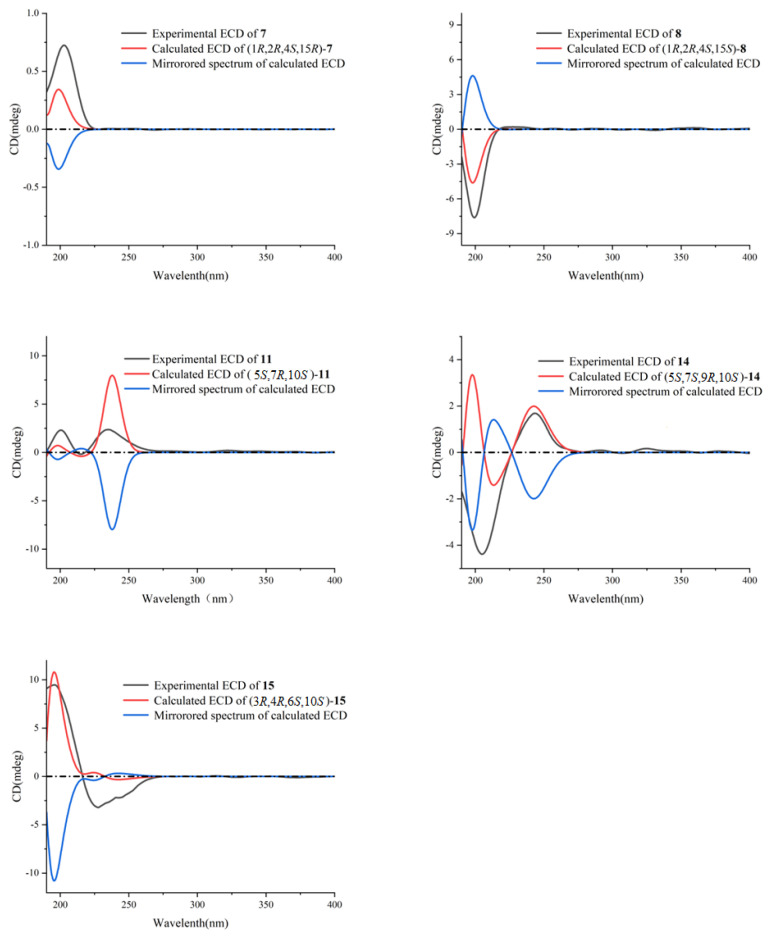
Comparison of experimental ECD spectrum (black) and DFT-predicted ECD spectra of **7**, **8**, **11**, **14**, **15**. ECD spectra were predicted by means of time-dependent DFT calculations at the MPW1PW91/6-31G(d, p) level.

Xishaklyane D (**4**) and xishaklyane E (**5**) were also isolated as a pair of epimers, which were separated by NP-HPLC (n-hexane/isopropanol (99.2:0.8), 1.0 mL/min) (**5**: *t*_R_ = 6.5 min; **6**: *t*_R_ = 5.6 min). The 1D and 2D NMR spectra of the mixture (Table 1 and Table 2) showed great similarity to those of the mixture of **2** and **3**, with the only differences in the chemical shifts between C-13 and C-17, indicating the opposite geometry of Δ^15,16^. The *Z* geometry of Δ^15,16^ was deduced by the ^1^H-^1^H coupling constants (*J* = 12.0 Hz). The ACs of **4** and **5** were also determined by TDDFT-ECD calculation. As shown in Figure 4, the Boltzmann-averaged ECD spectrum of (1*R*,2*R*,4*S*,13*R*,15*Z*)-**4** matched to the experimental ECD spectrum of **4**, while the Boltzmann-averaged ECD spectrum of (1*R*,2*R*,4*S*,13*S*,15*Z*)-**5** matched to the experimental ECD spectrum of **5**. Consequently, the ACs of **4** and **5** were determined to be 1*R*,2*R*,4*S*,13*R*,15*Z* and 1*R*,2*R*,4*S*,13*S*,15*Z*, respectively. Finally, xishaklyane D (**4**) was determined as 13*R*,15*Z*-isofuscol, while xishaklyane E (**5**) was determined to be 13*S*,15*Z*-isofusol. 

Xishaklyane F (**6**) was obtained as an optically active colourless oil. Its molecular formula was deduced to be C_22_H_34_O_3_ on the basis of HRESIMS (sodiated ion peak at *m/z* 369.2408 ([M + Na]^+^, calcd. 369.2400)), indicating six degrees of unsaturation. The ^1^H and ^13^C NMR data of **6** were reminiscent of those of the known compound **17** (Table 2 and Table 3), with the only difference on C-14 (*δ*_H_ 4.29, 4.31, *δ*_C_ 60.0 in **17,** and *δ*_H_ 4.75, 4.75, *δ*_C_ 61.6 in **6**), as well as an additional acetyl group in **6** (*δ*_H_ 2.07 s, *δ*_C_ 21.2, 171.3), indicating the acetylation of 14-OH of **17** towards **6**. To confirm our deduction, the acetylation of **17** (3 mg) was carried out using pyridine and Ac_2_O at room temperature for 24 h, yielding the acetate **6** (2 mg). Thus, compound **6** was determined as the 14-acetate of the known compound lobovarol H (**17**), namely xishaklyane F. 

Xishaklyanes G and H (**7** and **8**) were initially obtained as a mixture, which were further separated by NP-HPLC (n-hexane/isopropanol (90:10), 0.9 mL/min) (**7**: *t*_R_ = 6.4 min; **8**: *t*_R_ = 5.6 min). They showed the same pseudo molecular ion peak at *m/z* 303.2329 ([M − H]^−^, calcd. 303.2330) in the HRESIMS spectrum, and owned the same molecular formula of C_20_H_32_O_2_, indicating compound **7** to be isomeric with **8**. Detailed analysis of their NMR data suggested an epimeric relationship between **7** and **8**. The ^1^H and ^13^C NMR data of **7** and **8** (Table 2 and Table 3) showed difference at C-15 and its neighbouring carbons (e.g., C-3, C-5 and C-14), indicating **7** and **8** may have the opposite configuration of 15-OH. For the planar structure of both compounds, taking **7** for an example, its ^1^H and ^13^C NMR and HSQC resonances, as well as its coupling constants of the connected protons, indicated the presence of one monosubstituted terminal double bond [*δ*_H_ 5.81 (dd, *J* = 17.8, 10.5 Hz), 4.91 (d, *J* = 17.8 Hz), 4.90 (d, *J* = 10.5 Hz), *δ*_C_ 110.1 (t), *δ*_C_ 150.3 (s)], two disubstituted terminal olefinic bond [*δ*_H_ 4.82 (s), *δ*_H_ 4.58 (s), *δ*_C_ 112.4 (t), *δ*_C_ 147.7 (s); *δ*_H_ 4.99 (s), *δ*_H_ 5.15 (s), *δ*_C_ 109.0 (t), *δ*_C_ 156.0 (s)], and two monosubstituted double bond [*δ*_H_ 5.66 (dd, *J* = 15.6, 6.5 Hz), *δ*_H_ 5.90 (d, *J* = 15.6 Hz), *δ*_C_ 128.2 (d), *δ*_C_ 139.7 (d)]. The above olefinic bonds accounted for four degrees of unsaturation, so the remaining one degree should be ascribed to a ring in the molecule. Further analysis of ^1^H-^1^H COSY spectrum of compound **7** revealed three structural fragments **a–c**. These fragments were connected with well resolved HMBC correlations from H_3_-7 to C-1/C-2/C-6/C-8, from H_3_-12 to C-2/C-10/C-11, from H_3_-19/H_3_-20 to C-17/C-18, and from H_2_-14 to C-4/C-13/C-15 (Figure 2). Consequently, the planar structure of **7** was identified as shown in Figure 1, featuring a lobane-type diterpenoid skeleton. The determination of the relative configurations (RCs) of **7** and **8** were highly challenging, because of the chiral carbon on the side chain, whose RCs cannot be elucidated by only a NOESY experiment. The *E* geometry of Δ^16,17^ was deduced by the ^1^H-^1^H coupling constants (*J* = 15.6 Hz). NOE correlations of H-4/H-3*β*/H-2 revealed that these protons were disposed at the same side of the molecule and randomly assigned as *β*-oriented. The correlation of H_3_-7/H-3*α* revealed that these protons and proton-bearing groups were positioned at the other side of the molecule and were thus *α*-directed (Figure 2). The ACs of **7** and **8** were determined by the TDDFT-ECD calculation. As shown in Figure 4, the Boltzmann-averaged ECD spectrum of (1*R*,2*R*,4*S*,15*R*,16*E*)-**7** matched to the experimental ECD spectrum of **7**, while the Boltzmann-averaged ECD spectrum of (1*R*,2*R*,4*S*,15*S*,16*E*)-**8** matched to the experimental ECD spectrum of **8**. Consequently, the absolute configuration of **7** was determined to be 1*R*,2*R*,4*S*,15*R*,16*E*. The absolute configuration of **8** was determined to be 1*R*,2*R*,4*S*,15*S*,16*E*.

Xishaklyane I (**9**) was obtained as an optically active colourless oil. Its molecular formula of C_20_H_30_O was determined from the molecular ion peak at *m/z* 286.2288 ([M]^+^, calcd. 286.2291) in the HREIMS spectrum, indicating six degrees of unsaturation. Under detailed diagnostic 2D NMR spectra, as well as coupling constants of the connected protons (Table 2 and Table 3), compound **9** owned the same skeleton as the previously mentioned compounds. The major differences between them mainly happened at the C-14 position. An ether bridge between C-14 and C-16 formed a furan ring, which can be further verified by HMBC correlation from H-16 to C-14. As for the relative stereochemistry of **9**, the relative configurations at C-1, C-2, and C-4 were suggested to be the same as those of **1–8**, which was supported by the similar NOE relationships observed in the NOESY spectrum (Figure 2). Therefore, there are only two possibilities for the RC of **9** ((1R*, 2R*, 4S*, 16R*)-**9** and (1R*, 2R*, 4S*, 16S*)-**9**). Thus, the QM-NMR calculation and DP4+ analyses [27,28,29] of the ^13^C NMR parameter on the two possible candidate diastereoisomers were performed. Finally, the experimentally observed ^13^C NMR data for **9** gave its best match for the 1R*, 2R*, 4S*, 16R* isomer (**9a**, see the details in Supplementary Materials), with a 99.83% probability. Like the AC of fuscol, the absolute configuration of **9** was determined to be 1*R*,2*R*,4*S*,13*Z*,16*R*. 

The molecular formula C_20_H_30_O_2_ of xishaklyane J (**10**) was deduced by the HRESIMS pseudo molecular ion peak at *m/z* 303.2315 ([M + H]^+^ calcd. 303.2319), implying six degrees of unsaturation. The ^1^H and ^13^C NMR data of **10** (Table 2 and Table 3) were closely reminiscent of those of the co-occurrent 17,18-epoxyloba-8,10,13(15)-trien-16-ol (**18**). The only difference between them was the presence of a terminal alkene (C-13/C-14) and an epoxide (C-15/C-16) in **10** instead of a trisubstituted double bond (C-13/C-15) and a hydroxyl (C-16) in **18**. The detailed 2D NMR analysis shown in Figure 2 confirmed its planar structure. To further confirm the structure and RC of **10**, the QM-NMR calculation and DP4+ analyses were used. Finally, the experimentally observed NMR data for **10** gave its best match for the 1R*, 2R*, 4S*, 16S*, 17R*, 18R* isomer (**10d**, see the details in Supplementary Materials), with a 100.00% probability. Like the AC of fuscol, the absolute configuration of **10** was determined to be 1*R*,2*R*,4*S*,16*S*,17*R*,18*R*. 

**Figure 5:**
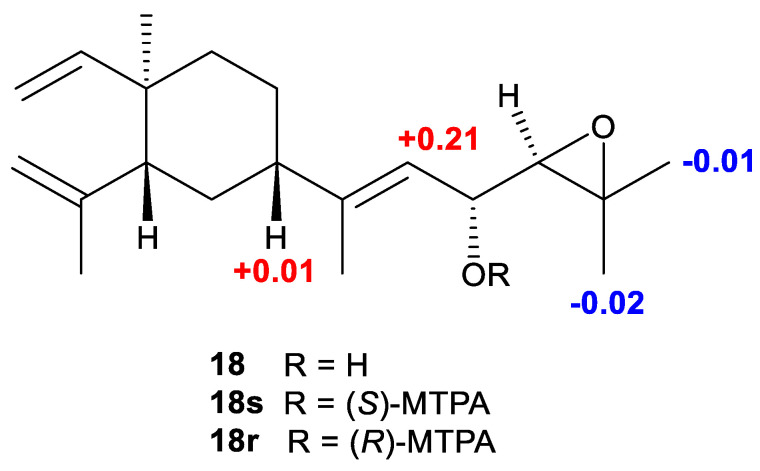
Δ*δ* values (*δ_S_*–*δ_R_*) (ppm) for (*S*)- and (*R*)-MTPA esters of compound **18**.

Biogenetically, **10** was believed to be derived from compound **18** by an acid induced electron delivery from 16-OH to first generate the 15,16-epoxyl and then promote the double bond migration towards the terminal olefin. The AC of the known compound 17,18-epoxyloba-8,10,13(15)-trien-16-ol (**18**) has not been defined. To obtain its absolute configuration at C-16, two aliquots of compound **18** were treated with (*R*)- and (*S*)-α-methoxy-α-trifluoromethylphenyl acetyl (MTPA) chlorides to obtain the (*S*)- and (*R*)-esters, respectively. Analysis of *Δδ^SR^* values (*δ_S_*–*δ_R_*) observed for the signals of the protons close to 16-OH indicated the *R* configuration at this carbon (Figure 5). 

**Figure 6:**
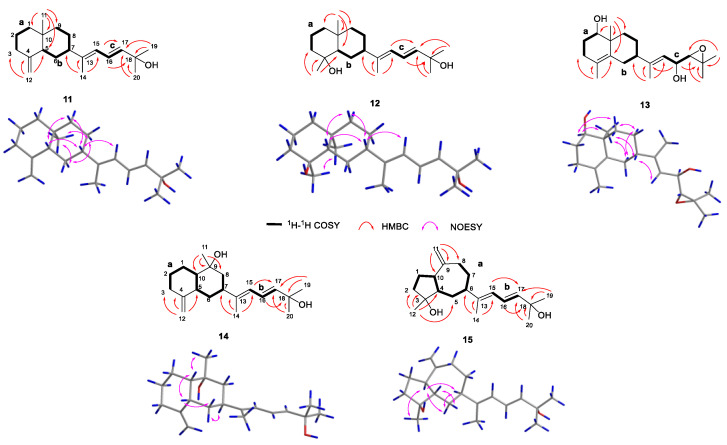
^1^H-^1^H COSY, key HMBC, and NOESY correlations of compounds **11–15**.

Xishaklyane K (**11**) was obtained as an optically active colourless oil. Its molecular formula was deduced to be C_20_H_32_O on the basis of the HREIMS (molecular ion peak at *m/z* 288.2443 ([M]^+^, calcd. 288.2448)). Careful analysis of the 1D NMR spectra of **11** (Table 4 and Table 5) showed a close similarity with those of co-occurring **16**, indicating **11** as also being a same side carbon chain at C-7. Further analysis of its 1D and 2D NMR spectra revealed that the main difference was between C-1 and C-12. The ^1^H-^1^H COSY correlation of H_2_-2/H_2_-3 formed a six-membered ring, which can also be deduced from the HMBC correlation from H_2_-3 to C-1. Therefore, the planar structure was identified, as shown in Figure 1, as a prenyleudesmane type diterpene. The remaining task was to determine the structure and RC of **11**. The *E* geometry of Δ^16,17^ was deduced by the ^1^H-^1^H coupling constants (*J* = 15.3 Hz) and the *E* geometry of Δ^13,15^ was deduced by the NOE correlations between H-15 and H-7 (Figure 6). The relative configuration of chiral centres C-10, C-5, and C-7 in compound **11** was the same as those compounds mentioned above. To determine the AC of **11**, a TDDFT-ECD calculation was performed. As shown in Figure 4, the Boltzmann-averaged ECD spectrum of (5*S*,7*R*,10*S*)-**11** matched to the experimental ECD spectrum of **11**. Consequently, the AC of **11** was determined to be 5*S*,7*R*,10*S*,13*E*,16*E*.

Xishaklyane L (**12**) was obtained as an optically active colourless oil. From the molecular ion peak at *m/z* 306.2553 ([M]^+^, calcd. 306.2553) in the HREIMS spectrum, a molecular formula of C_20_H_32_O_2_ was established. The structural features of **12** were reminiscent of the known compound lobovarol K [20]. Compound **12** was methylated at the hydroxyl of C-18 to form lobovarol K. The same NOE correlations (Figure 6) meant that the structure of **12** was tentatively determined to be the same as lobovarol K.

Xishaklyane M (**13**) has a molecular formula C_20_H_32_O_3_, as displayed from the ion peak in the HREIMS (*m*/*z* 320.2347 [M]^+^). The 1D and 2D NMR data of **13** (Table 4 and Table 5) further revealed that it was a diterpene possessing the same double rings with identical substitutions as that of the eudesmane derivative. The NOE correlations between H_3_-11 and H-6*α*/H-8*α*, H-6*α* and H-8*α*, H-1, and H-9*α*, and between H-7 and H-9*β*, suggested the *β*-orientation of H-7, and the *α*-orientation of H_3_-11 and H-1, respectively (Figure 6). The side chain was established by detailed 1D and 2D NMR data of **13**, indicating a hydroxyl at C-16 and an epoxide at C-17/C-18, which is the same as the known compound **18**. Comparing their NMR data, the absolute configuration of **13** was tentatively determined to be 1*R*,4*Z*,7*S*,10S,13*E*,16*R*,17*R*. 

Xishaklyane N (**14**) displayed a molecular formula of C_20_H_32_O_2_ as established by the HREIMS ion peak at *m/z* 304.2400 ([M]^+^ calcd for 304.2397). The ^1^H and ^13^C NMR data of **14** (Table 4 and Table 5) were very similar with **11**. Detailed analysis of those spectra of **14,** and comparing with those of **11,** revealed that the main differences between them happened at C-9 to C-11 segments. The HMBC correlations from H_3_-11 to three carbons (C-8/C-9/C-10) instead of four carbons (C-5/C-8/C-9/C-10) in **11** indicated that a methyl was displaced at C-9. As for the stereochemistry of compound **14**, the chemical shifts of C-14 (*δ*_C_ 15.4, CH_3_) and *J* value of H-16 and H-17 (15.3 Hz) indicated the *E*-geometry for the 13,15- and 16,17-double bonds, respectively. The NOE correlations between H_3_-11 and H-10, H-5 and H-10, H-5 and H-6*β*, and between H-7 and H-6*α*, suggested the *cis*-decahydronaphthalene core structure, where H-7 and H-10 are axial orientation while H-5 and Me-9 are equatorial orientation (Figure 6). RC of **14**: 5S*, 7S*, 9R*, 10S*. As shown in Figure 4, the Boltzmann-averaged ECD spectrum of (5*S*,7*S*,9*R*,10*S*)-**14** matched to the experimental ECD spectrum of **14**. Consequently, the AC of **14** was determined to be 5*S*,7*S*,9*R*,10*S*,13*E*,16*E*.

Xishaklyane O (**15**) was also obtained as an optically active colourless oil. From the molecular ion peak at *m/z* 304.2402 ([M]^+^, calcd. 304.2397) in the HREIMS spectrum, a molecular formula of C_20_H_32_O_2_ was established, indicating five degrees of unsaturation. Detailed analysis of 1D and 2D NMR spectra (Table 4 and Table 5) revealed that it had the same side chain as compounds **11**, **12**, **14**, and **16**. Further analysis of ^1^H-^1^H COSY and the HMBC spectrum of **15** revealed a 5/7-fused carbon ring system (Figure 6). As for the stereochemistry of compound **15**, the *E* geometry of Δ^16,17^ was deduced by the ^1^H-^1^H coupling constants (*J* = 15.3 Hz), and the *E* geometry of Δ^13,15^ was deduced by the chemical shifts of C-14 (*δ*_C_ 14.9, CH_3_). The NOE correlations between H-5*β* and H-10/H-6, H-5*α* and H-4, H-10 and H-6, and between H_3_-12 and H-10, suggested the *α*-orientation of H-10, H-4, and H_3_-12, and the *β*-orientation of H-6, respectively. RC of **15**: 3R*, 4R*, 6S*, 10S*. As shown in Figure 4, the Boltzmann-averaged ECD spectrum of (3*R*,4*R*,6*S*,10*S*)-**15** matched to the experimental ECD spectrum of **15**. Consequently, the absolute configuration of **15** was determined to be 3*R*,4*R*,6*S*,10*S*,13*E*,16*E*. 

All compounds were screened for antibacterial activities on fish pathogenic bacteria. As shown in Table 6, some of them exhibited considerable antibacterial activities. Among them, compound **4** was the most effective one, with an MIC of 0.225 μg/mL against *Lactococcus garvieae*, whereas compound **11** showed the best antibacterial activity against *Streptococcus parauberis*, with an MIC of 0.9 μg/mL. 

## 3. Materials and Methods

### 3.1. General Experimental Procedures

Optical rotations were measured on a Perkin-Elmer 241MC polarimeter (PerkinElmer, Fremont, CA, USA). UV and CD spectra were recorded on a Jasco J-815 spectropolarimeter (JASCO, Tokyo, Japan) at ambient temperature using chromatographic grade CH_3_OH and CH_3_CN as solvents. IR spectra were recorded on a Nicolet 6700 spectrometer (Thermo Scientific, Waltham, MA, USA); peaks are reported in cm^−1^. The NMR spectra were measured at 300 K on Bruker Avance III 400, 500, or 600 MHz NMR spectrometers (Bruker Biospin AG, Fällanden, Germany). Chemical shifts are reported in parts per million (*δ*) in CDCl_3_ (*δ*_H_ reported referred to CHCl_3_ at 7.26 ppm; *δ*_C_ reported referred to CDCl_3_ at 77.16 ppm) and coupling constants (*J*) in Hz; assignments were supported by ^1^H-^1^H COSY, HSQC, HMBC, and NOESY experiments. HREIMS data were recorded on a Finnigan-MAT-95 mass spectrometer (Finnigan-MAT, San Jose, CA, USA). HRESIMS spectra were recorded on an Agilent G6520 Q-TOF mass spectrometer (Agilent, Santa Clara, CA, USA). Semi-preparative RP-HPLC was performed on an Agilent-1260 system (Agilent, Santa Clara, CA, USA) equipped with a DAD G1315D detector at 210 and 254 nm using XDB-C18 column (250 mm × 9.4 mm, 5 µm) by eluting with CH_3_OH-H_2_O or CH_3_CN-H_2_O system at 3 mL/min. NP-HPLC was performed on a Shimadzu LC-6A system (Shimadzu, Kyoto, Japan) equipped with a DAD SPD-M20A detector using CHIRALPAK^®^ IA or CHIRALPAK^®^ IC (250 mm × 4.6 mm, 5 µm, Daicel Corporation, Osaka, Japan) by eluting with n-hexane-isopropanol system at 1 mL/min. Commercial silica gel (100–200, 200–300, and 300–400 mesh; Qingdao Haiyang Chemical Group Co., Ltd., Qingdao, China) was used for column chromatography (CC). Precoated silica gel GF254 plates (Sinopharm Chemical Reagent Co., Shanghai, China) were used for analytical TLC. Spots were detected on TLC under UV light or by heating after spraying with anisaldehyde H_2_SO_4_ reagent. Sephadex LH-20 (Amersham Biosciences, Little Chalfont, UK) was also used for CC. All solvents used for column chromatography and HPLC were of analytical grade (Shanghai Chemical Reagents Co., Ltd., Shanghai, China) and chromatographic grade (Dikma Technologies Inc., Foothill Ranch, CA, USA), respectively. The antibiotics tetracycline, oxytetracycline hydrochloride, levofloxacin hydrochloride, and ampicillin sodium were purchased from Sigma-Aldrich^®^ (Darmstadt, Germany).

### 3.2. Animal Material

Specimens of the soft coral *Klyxum molle* were collected by scuba diving at a depth of −20 m in Xisha Islands, Hainan Province, China, in 2019, and were identified by Professor Xiu-Bao Li (Hainan University, Hainan, China). The biological material was frozen immediately after collection. A voucher specimen (19-XS-41) is available for inspection at the Shanghai Institute of Materia Medica, Chinese Academy of Sciences.

### 3.3. Extraction and Isolation

The frozen animals (868 g, dry weight) of *K. molle* were cut into pieces and extracted exhaustively with acetone at room temperature (3 × 3.0 L). The organic extract was evaporated to give a brown residue, which was then partitioned between Et_2_O and H_2_O. The upper layer was concentrated under reduced pressure to give a brown residue (43.3 g), which was fractioned by gradient silica gel (200–300 mesh) column chromatography (CC) (0~100% Et_2_O in petroleum ether (PE)), yielding 11 fractions (A–K). Fr. D was fractioned by Sephadex LH-20 (PE/CH_2_Cl_2_/MeOH, 2:1:1) to obtain three sub-fractions Fr. Da, Db, and Dc. The subfraction Dc was separated on a column of silica gel (10~20% Et_2_O in PE) to afford **16** (1.31 g) and **1** (0.26 g). Fr. C was fractioned by Sephadex LH-20 (PE/CH_2_Cl_2_/MeOH, 2:1:1) to obtain four sub-fractions, Fr. Ca, Cb, Cc and Cd. The subfraction Cd was separated on a column of silica gel (5~20% Et_2_O in PE) to afford mixtures Cd2 and Cd6. Cd2 was further purified by RP-HPLC (MeOH/H_2_O (90:10), 3.0 mL/min) to give **10** (3.9 mg, *t*_R_ = 11.0 min) and Cd2c (0.7 mg, *t*_R_ = 11.8 min). Cd6 was further purified by RP-HPLC (MeOH/H_2_O (95:5), 3.0 mL/min) to give **11** (1.1 mg, *t*_R_ = 8.0 min) and Cd6a (3.1 mg, *t*_R_ = 5.3 min). Cd2c was purified by NP-HPLC (n-hexane/isopropanol (99.2:0.8), 1.0 mL/min) to give **4** (0.5 mg, *t*_R_ = 6.5 min) and **5** (0.2 mg, *t*_R_ = 5.6 min). Cd6a was purified by NP-HPLC (n-hexane/isopropanol (99.7:0.3), 1.0 mL/min) to give **2** (1.4 mg, *t*_R_ = 4.6 min) and **3** (1.4 mg, *t*_R_ = 5.5 min). Fr. G was fractioned by Sephadex LH-20 (PE/CH_2_Cl_2_/MeOH, 2:1:1) to obtain three sub-fractions, Fr. Ga, Gb, and Gc. Gb was further purified by RP-HPLC (MeOH/H_2_O (85:15), 3.0 mL/min) to give **17** (0.9 mg, *t*_R_ = 8.3 min) and **18** (3.7 mg, *t*_R_ = 13.7 min). Gc was further purified by RP-HPLC (MeCN/H_2_O (60:40), 3.0 mL/min) to give **9** (0.8 mg, *t*_R_ = 10.4 min) and **13** (0.6 mg, *t*_R_ = 5.3 min). Fr. H was fractioned by Sephadex LH-20 (PE/CH_2_Cl_2_/MeOH, 2:1:1) to obtain three sub-fractions, Fr. Ha, Hb, and Hc. The subfraction Hc was separated on a column of silica gel (50~75% Et_2_O in PE) to afford **6** (15.0 mg) and mixtures Hc1 and Hc2. Hc2 was further purified by RP-HPLC (MeCN/H_2_O (70:30), 3.0 mL/min) to give **14** (2.0 mg, *t*_R_ = 9.3 min). Hc1 was further purified by RP-HPLC (MeCN/H_2_O (55:45), 2.0 mL/min) to give **12** (7.0 mg, *t*_R_ = 21.3 min), **15** (1.0 mg, *t*_R_ = 18.2 min), and Hc1d (1.3 mg, *t*_R_ = 23.6 min). Hc1d was purified by NP-HPLC (n-hexane/isopropanol (90:10), 0.9 mL/min) to give **7** (0.5 mg, *t*_R_ = 6.4 min) and **8** (0.7 mg, *t*_R_ = 5.6 min).

### 3.4. Spectroscopic Data of Compounds

Xishaklyane A (**1**): Colourless oil; [α${]}_{D}^{20}$ −43.1 (c 1.95, CHCl_3_); UV (MeCN) *λ*_max_ (log *ε*) 240 (3.31) nm; ECD (CH_3_CN) *λ*_max_ (Δ*ε*) 215 (−5.2) nm; IR (KBr) *ν*_max_ 3382, 2969, 2928, 2860, 1637, 1440, 1376, 1148,1005 cm^−1^; HRESIMS [M + H]^+^ *m/z* 289.2527 (calcd. for 289.2526, C_20_H_33_O).

Xishaklyane B (**2**): Colourless oil; [α${]}_{D}^{20}$ –7.4 (c 0.14, CHCl_3_); UV (MeCN) *λ*_max_ (log *ε*) 238 (3.23) nm; ECD (CH_3_CN) *λ*_max_ (Δ*ε*) 201 (−1.6) nm.

Xishaklyane C (**3**): Colourless oil; [α${]}_{D}^{20}$ +30.2 (c 0.14, CHCl_3_); UV (MeCN) *λ*_max_ (log *ε*) 238 (3.27) nm; ECD (CH_3_CN) λ_max_ (Δ*ε*) 232 (+2.3) nm.

Xishaklyane D (**4**) and Xishaklyane E (**5**): For **4**, Colourless oil; [α${]}_{D}^{20}$ −35.0 (c 0.05, CHCl_3_); UV (MeCN) *λ*_max_ (log *ε*) 240 (3.29) nm; ECD (CH_3_CN) *λ*_max_ (Δ*ε*) 240 (−3.4) nm; For **5**, Colourless oil; [α${]}_{D}^{20}$ +75.8 (c 0.02, CHCl_3_); UV (MeCN) *λ*_max_ (log *ε*) 240 (3.35) nm; ECD (CH_3_CN) *λ*_max_ (Δ*ε*) 244 (+3.8) nm; For mixture of **4** and **5**, IR (KBr) *ν*_max_ 3455, 2966, 2925, 2854, 1438, 1376, 1180, 1142, 1099, 1075,1029 cm^−1^; HREIMS [M]^+^ *m/z* 288.2454 (calcd. for 288.2448, C_20_H_32_O).

Xishaklyane F (**6**): Colourless oil; [α${]}_{D}^{20}$ +33.5 (c 0.09, CHCl_3_); UV (MeCN) *λ*_max_ (log *ε*) 239 (3.30) nm; ECD (CH_3_CN) *λ*_max_ (Δ*ε*) 234 (+2.9) nm; IR (KBr) *ν*_max_ 3451, 2968, 2925, 2854, 1735, 1377, 1260, 1230, 1075,1027 cm^−1^; HRESIMS [M + Na]^+^ *m/z* 369.2408 (calcd. for 369.2400, C_22_H_34_NaO_3_).

Xishaklyane G (**7**) and Xishaklyane H (**8**): For **7**, Colourless oil; [α${]}_{D}^{20}$ +25.3 (c 0.05, CHCl_3_); ECD (CH_3_CN) *λ*_max_ (Δ*ε*) 196 (−4.4) nm; For **8**, White solid; [α${]}_{D}^{20}$ +24.0 (c 0.07, CHCl_3_); ECD (CH_3_CN) *λ*_max_ (Δ*ε*) 204 (+5.6) nm; For mixture of **7** and **8**, IR (KBr) *ν*_max_ 3443, 2968, 2924, 2853, 1384, 1180, 1143, 1095, 1076, 1029 cm^−1^; HRESIMS [M − H]^−^ *m/z* 303.2329 (calcd. for 303.2330, C_20_H_31_O_2_).

Xishaklyane I (**9**): Colourless oil; [α${]}_{D}^{20}$ +67.9 (c 0.08, CHCl_3_); IR (KBr) *ν*_max_ 3451, 2967, 2925, 2854, 1444, 1374, 1180, 1059, 1029 cm^−1^; HREIMS [M]^+^ *m/z* 286.2288 (calcd. for 286.2291, C_20_H_30_O).

Xishaklyane J (**10**): Colourless oil; [α${]}_{D}^{20}$ +2.8 (c 0.09, CHCl_3_); IR (KBr) *ν*_max_ 3454, 2965, 2926, 2856, 1643, 1456,1379, 1075, 1029 cm^−1^; HRESIMS [M + H]^+^ *m/z* 303.2315 (calcd. for 303.2319, C_20_H_31_O_2_).

Xishaklyane K (**11**): Colourless oil; [α${]}_{D}^{20}$ −20.0 (c 0.11, CHCl_3_); UV (MeCN) *λ*_max_ (log *ε*) 240 (3.34) nm; ECD (CH_3_CN) *λ*_max_ (Δ*ε*) 200 (+2.0) nm; IR (KBr) *ν*_max_ 3451, 2869, 2925, 2852, 1442, 1386, 1180, 1143, 1075, 1030 cm^−1^; HREIMS [M]^+^ *m/z* 288.2443 (calcd. for 288.2448, C_20_H_32_O).

Xishaklyane L (**12**): Colourless oil; [α${]}_{D}^{20}$ +25.7 (c 0.70, CHCl_3_); UV (MeCN) *λ*_max_ (log *ε*) 240 (3.33) nm; ECD (CH_3_CN) *λ*_max_ (Δ*ε*) 242 (+2.1) nm; IR (KBr) *ν*_max_ 3385, 2970, 2926, 2864, 1456, 1384, 1143, 1105 cm^−1^; HREIMS [M]^+^ *m/z* 306.2553 (calcd. for 306.2553, C_20_H_34_O_2_).

Xishaklyane M (**13**): Colourless oil; [α${]}_{D}^{20}$ −13.3 (c 0.06, CHCl_3_); IR (KBr) *ν*_max_ 3450, 2963, 2925, 2854, 1436, 1378, 1180, 1075, 1028 cm^−1^; HREIMS [M]^+^ *m/z* 320.2347 (calcd. for 320.2346, C_20_H_32_O_3_).

Xishaklyane N (**14**): Colourless oil; [α${]}_{D}^{20}$ −15.5 (c 0.20, CHCl_3_); UV (MeCN) *λ*_max_ (log *ε*) 239 (3.34) nm; ECD (CH_3_CN) *λ*_max_ (Δ*ε*) 210 (−1.9) nm; IR (KBr) *ν*_max_ 3450, 2963, 2925, 2857, 1386, 1180, 1143, 1095, 1075, 1028 cm^−1^; HREIMS [M]^+^ *m/z* 304.2400 (calcd. for 304.2397, C_20_H_32_O_2_).

Xishaklyane O (**15**): Colourless oil; [α${]}_{D}^{20}$ −16.8 (c 0.10, CHCl_3_); UV (MeCN) *λ*_max_ (log *ε*) 240 (3.38) nm; ECD (CH_3_CN) *λ*_max_ (Δ*ε*) 197 (+4.2) nm; IR (KBr) *ν*_max_ 3450, 2959, 2923, 2852, 1384, 1180, 1143, 1129, 1099, 1075, 1029 cm^−1^; HREIMS [M]^+^ *m/z* 304.2402 (calcd. for 304.2397, C_20_H_32_O_2_).

### 3.5. Esterification of **18** with MTPA Chlorides

Compound **18** (2.0 mg) was dissolved in dry pyridine (1000 μL), and the solution was transferred into two NMR tubes (500 μL each), treated with (*R*)-(−)-2-methoxy-2-(trifluoromethyl) phenylacetyl chloride ((*R*)-(−)-MTPA-Cl) (20 μL) and (*S*)-(+)-2-methoxy-2-(trifluoromethyl) phenylacetyl chloride ((*S*)-(+)-MTPA-Cl) (20 μL), respectively. They were carefully shaken and then monitored immediately by ^1^H NMR. The reaction was found to be completed in 30 min. Then the solutions were evaporated in vacuo and the residue was purified by silica gel CC (10% Et_2_O in PE) to obtain the *S*-MTPA ester **18s**, and *R*-MTPA ester **18r**, respectively. For (*S*)-MTPA ester of **18** (**18s**), Selected ^1^H NMR (CDCl_3_, 400 MHz): δ_H_ 5.812 (1H, dd, *J* = 17.6, 10.7 Hz, H-8), 5.626 (1H, t, *J* = 9.2 Hz, H-16), 5.359 (1H, d, *J* = 9.8 Hz, H-15), 4.917 (1H, d, *J* = 15.9 Hz, H-9α), 4.912 (1H, d, *J* = 12.4 Hz, H-9β), 4.842 (1H, s, H-11α), 4.585 (1H, s, H-11β), 2.997 (1H, d, *J* = 8.4 Hz, H-17), 2.009 (1H, d, *J* = 12.2 Hz, H-4), 1.827 (3H, s, Me-14), 1.713 (3H, s, Me-12), 1.338 (3H, s, Me-20), 1.323 (3H, s, Me-19), 1.013 (3H, s, Me-7). For (*R*)-MTPA ester of **18** (**18r**), Selected ^1^H NMR (CDCl_3_, 400 MHz): δ_H_ 5.807 (1H, dd, *J* = 17.6, 10.6 Hz, H-8), 5.591 (1H, t, *J* = 9.2 Hz, H-16), 5.148 (1H, d, *J* = 10.1 Hz, H-15), 4.911 (1H, d, *J* = 16.6 Hz, H-9α), 4.910 (1H, d, *J* = 11.5 Hz, H-9β), 4.848 (1H, s, H-11α), 4.579 (1H, s, H-11β), 2.996 (1H, d, *J* = 8.6 Hz, H-17), 1.995 (1H, d, *J* = 11.9 Hz, H-4), 1.856 (3H, s, Me-14), 1.713 (3H, s, Me-12), 1.358 (3H, s, Me-20), 1.331 (3H, s, Me-19), 0.997 (3H, s, Me-7).

### 3.6. QM-NMR Calculational Section

For the QM-NMR calculations of compounds, conformational search was performed by using the torsional sampling (MCMM) approach and OPLS_2005 force field within an energy window of 21 kJ/mol. The following DFT calculations were performed using Gaussian 09, and Conformers above 1% population were reoptimized at the B3LYP/6-311G(d, p) level of theory. Magnetic shielding constants (σ) were calculated by means of the gauge including atomic orbitals (GIAO) method at the mPW1PW91/6-31+G(d, p) level of theory, as recommended for DP4+ analysis.

### 3.7. TDDFT-ECD Calculational Section

For the time-dependent density functional theory/electronic circular dichroism (TDDFT-ECD) calculations of compounds, conformational searches were performed following the general protocols previously described for QM-NMR calculation. Conformers above 1% population were used for re-optimizations and the following TDDFT-ECD calculations, which were performed using Gaussian 09 at the B3LYP/6-311G(d, p) level of theory with an IEFPCM solvent model for acetonitrile. Finally, the SpecDis 1.62 software was used to obtain the calculated ECD spectrum and visualize the results.

### 3.8. Antibacterial Assays

Five pathogenic bacteria, namely *Streptococcus parauberis* KSP28, *Streptococcus parauberis* SPOF3K, *Lactococcus garvieae* MP5245, *Aeromonas salmonicida* AS42, and *Photobacterium damselae* FP2244, were provided by the National Fisheries Research and Development Institute, Korea. The MIC values for all antimicrobial agents were measured by 96-well micro-dilution method. Mueller–Hinton II broth (cation-adjusted, BD 212322) was used for MIC value determination. Generally, compounds were dissolved with DMSO to 20 mM as stock solutions. All samples were diluted with culture broth to 500 μM as the initial concentration. Further 1:2 serial dilutions were performed through the addition of culture broth in order to reach concentrations ranging from 500 μM to 0.24 μM. A volume of 100 μL of each dilution was distributed in 96-well plates, as well as sterile controls, growth controls (containing culture broth plus DMSO, without compounds), and positive controls (containing culture broth plus positive control antibiotics). Each test and growth control wells were inoculated with 5 μL of an exponential-phase bacterial suspension (about 10^5^ CFU/well). The 96-well plates were incubated at 37 °C for 24 h. MIC vales of these compounds were defined as the lowest concentration needed to inhibit the bacterial growth completely. The units of the MIC values were transferred from “μM” to “μg/mL” according to compound’s molecular weight. All MIC values were interpreted according to recommendations of the Clinical and Laboratory Standards Institute (CLSI).

## 4. Conclusions

This detailed chemical investigation on the South China Sea soft coral *K. molle* yielded fifteen new diterpenes, namely xishaklyanes A-O (**1–15**), as well as three related known analogues (**16**–**18**). Among them, the absolute configuration of compound **18** was determined by the modified Mosher’s method. In addition, the stereochemistry of the new compounds was determined by QM-NMR and TDDFT-ECD, or chemical connections. In antibacterial activities on fish pathogenic bacteria, compound **4** exhibited the best activity, with an MIC of 0.225 μg/mL against *Lactococcus garvieae*. Further study should be conducted on the accumulation of the most effective compounds for in-depth antibacterial research.

## Figures and Tables

**Table 1:** The ^1^H NMR data (600 MHz, *δ*_H_ in ppm, *J* in Hz) for compounds **1**–**5** in CDCl_3_.

No.	1	2	3	4	5
	*δ*_H_ Mult. (*J* Hz)	*δ*_H_ Mult. (*J* Hz)	*δ*_H_ Mult. (*J* Hz)	*δ*_H_ Mult. (*J* Hz)	*δ*_H_ Mult. (*J* Hz)
2	2.08, dd (12.9, 3.3)	1.94, dd (11.8, 3.2)	1.94, dd (11.8, 3.2)	1.97, dd (11.8, 3.3)	1.97, dd (11.8, 3.3)
3a	1.68, m	1.43, m	1.43, m	1.50, m	1.50, m
3b	1.74, m	1.59, m	1.59, m	1.62, m	1.62, m
4	2.70, tt (12.1, 3.8)	1.43, m	1.43, m	1.50, m	1.50, m
5a	1.38, m	1.44, m	1.44, m	1.33, m	1.33, m
5b	1.59, m	1.63, m	1.63, m	1.45, m	1.45, m
6a	1.44, m	1.44, m	1.44, m	1.46, m	1.46, m
6b	1.55, m	1.44, m	1.44, m	1.46, m	1.46, m
7	1.02, s	0.97, s	0.97, s	0.97, s	0.97, s
8	5.83, dd (17.2, 11.3)	5.80, dd (15.3, 10.9)	5.80, dd (15.3, 10.9)	5.80, dd (15.3, 10.9)	5.80, dd (15.3, 10.9)
9a	4.90, d (11.3)	4.88, d (10.9)	4.88, d (10.9)	4.88, d (10.9)	4.88, d (10.9)
9b	4.90, d (17.2)	4.89, d (15.3)	4.89, d (15.3)	4.89, d (15.3)	4.89, d (15.3)
11a	4.59, s	4.58, s	4.58, s	4.58, s	4.58, s
11b	4.81, s	4.81, s	4.81, s	4.81, s	4.81, s
12	1.71, s	1.70, s	1.70, s	1.70, s	1.70, s
14	1.76, s	1.29, s	1.29, s	1.35, s	1.35, s
15	5.77, d (10.9)	5.65, d (15.3)	5.65, d (15.3)	5.31, d (11.9)	5.31, d (11.9)
16	6.50, dd (15.2, 10.9)	6.43, dd (15.3, 10.9)	6.43, dd (15.3, 10.9)	6.19, t (11.9)	6.19, t (11.9)
17	5.70, d (15.2)	5.84, d (10.9)	5.84, d (10.9)	6.63, d (11.9)	6.63, d (11.9)
19	1.34, s	1.78, s	1.78, s	1.74, s	1.74, s
20	1.34, s	1.78, s	1.78, s	1.81, s	1.81, s

Chemical shifts (ppm) refer to CHCl_3_ (*δ*_H_ 7.26). Assignments were deduced by analysis of 1D and 2D NMR spectra.

**Table 2:** The ^13^C NMR data (125 MHz, *δ*_C_ in ppm) for compounds **1–10** in CDCl_3_.

No.	1	2	3	4	5	6	7	8	9	10
	*δ*_C_ Mult.	*δ*_C_ Mult.	*δ*_C_ Mult.	*δ*_C_ Mult.	*δ*_C_ Mult.	*δ*_C_ Mult.	*δ*_C_ Mult.	*δ*_C_ Mult.	*δ*_C_ Mult.	*δ*_C_ Mult.
1	39.7, C	39.9, C	39.9, C	39.9, C	39.9, C	39.9, C	39.9, C	39.9, C	39.9, C	39.8, C
2	52.6, CH	52.8, CH	52.8, CH	52.7, CH	52.8, CH	52.9, CH	53.0, CH	53.0, CH	52.6, CH	52.8, CH
3	31.9, CH_2_	28.2, CH_2_	28.5, CH_2_	28.1, CH_2_	28.5, CH_2_	33.3, CH_2_	34.3, CH_2_	34.7, CH_2_	33.1, CH_2_	33.9, CH_2_
4	40.5, CH	48.8, CH	48.9, CH	49.5, CH	49.6, CH	41.1, CH	40.9, CH	41.0, CH	37.2, CH	41.1, CH
5	26.0, CH_2_	22.7, CH_2_	22.4, CH_2_	22.6, CH_2_	22.2, CH_2_	27.2, CH_2_	28.7, CH_2_	28.2, CH_2_	27.2, CH_2_	27.1, CH_2_
6	39.8, CH_2_	40.0, CH_2_	39.9, CH_2_	40.0, CH_2_	40.0, CH_2_	40.0, CH_2_	40.2, CH_2_	40.1, CH_2_	39.7, CH_2_	39.8, CH_2_
7	16.8, CH_3_	16.7, CH_3_	16.7, CH_3_	16.7, CH_3_	16.7, CH_3_	16.8, CH_3_	16.8, CH_3_	16.8, CH_3_	16.7, CH_3_	16.7, CH_3_
8	150.4, CH	150.4, CH	150.4, CH	150.5, CH	150.5, CH	150.2, CH	150.3, CH	150.3, CH	150.1, CH	150.1, CH
9	110.1, CH_2_	110.0, CH_2_	110.0, CH_2_	110.0, CH_2_	110.0, CH_2_	110.2, CH_2_	110.1, CH_2_	110.1, CH_2_	110.2, CH_2_	110.3, CH_2_
10	147.7, C	148.1, C	148.0, C	148.1, C	148.0, C	147.6, C	147.6, C	147.6, C	147.5, C	147.4, C
11	112.3, CH_2_	112.2, CH_2_	112.2, CH_2_	112.2, CH_2_	112.2, CH_2_	112.4, CH_2_	112.3, CH_2_	112.4, CH_2_	112.4, CH_2_	112.4, CH_2_
12	25.0, CH_3_	24.9, CH_3_	24.9, CH_3_	24.8, CH_3_	24.9, CH_3_	24.9, CH_3_	24.9, CH_3_	24.9, CH_3_	24.9, CH_3_	25.0, CH_3_
13	143.1, C	75.0, C	75.1, C	76.7, C	76.7, C	140.2, C	156.0, C	156.0, C	145.7, C	149.2, C
14	20.2, CH_3_	26.2, CH_3_	26.2, CH_3_	27.4, CH_3_	27.5, CH_3_	61.6, CH_2_	109.1, CH_2_	108.9, CH_2_	75.3, CH_2_	109.4, CH_2_
15	124.6, CH	135.3, CH	135.2, CH	133.5, CH	133.4, CH	128.4, CH	75.0, CH	74.9, CH	120.9, CH	56.0, CH
16	122.0, CH	124.4, CH	124.4, CH	125.5, CH	125.5, CH	122.0, CH	128.1, CH	128.2, CH	83.1, CH	58.5, CH
17	139.3, CH	124.8, CH	124.8, CH	121.4, CH	121.5, CH	143.0, CH	139.6, CH	139.6, CH	125.7, CH	63.0, CH
18	71.1, C	137.0, C	137.0, C	137.3, C	137.3, C	71.1, C	70.8, C	70.8, C	135.5, C	58.6, C
19	30.2, CH_3_	18.5, CH_3_	18.5, CH_3_	17.8, CH_3_	17.8, CH_3_	29.9, CH_3_	29.9, CH_3_	29.9, CH_3_	18.2, CH_3_	19.7, CH_3_
20	30.2, CH_3_	26.0, CH_3_	26.1, CH_3_	26.7, CH_3_	26.7, CH_3_	29.9, CH_3_	30.0, CH_3_	29.9, CH_3_	26.0, CH_3_	24.7, CH_3_
21						171.3, C				
22						21.2, CH_3_				

Chemical shifts (ppm) refer to CHCl_3_ (*δ*_C_ 77.2). Assignments were deduced by analysis of 1D and 2D NMR spectra.

**Table 3:** The ^1^H NMR data (600 MHz, *δ*_H_ in ppm, *J* in Hz) for compounds **6**–**10** in CDCl_3_.

No.	6	7	8	9	10
	*δ*_H_ Mult. (*J* Hz)	*δ*_H_ Mult. (*J* Hz)	*δ*_H_ Mult. (*J* Hz)	*δ*_H_ Mult. (*J* Hz)	*δ*_H_ Mult. (*J* Hz)
2	2.02, dd (10.7, 5.4)	1.99, dd (10.7, 5.4)	1.99, dd (10.7, 5.4)	2.01, dd (12.7, 3.4)	2.02, dd (12.8, 3.2)
3a	1.57, m	1.57, m	1.57, m	1.61, m	1.53, m
3b	1.57, m	1.57, m	1.57, m	1.61, m	1.60, m
4	2.11, m	1.99, m	1.99, m	2.10, t (11.9)	1.99, m
5a	1.48, m	1.47, m	1.47, m	1.44, m	1.50, m
5b	1.63, m	1.62, m	1.62, m	1.68, m	1.62, m
6a	1.48, m	1.45, m	1.45, m	1.46, m	1.48, m
6b	1.51, m	1.51, m	1.51, m	1.51, m	1.51, m
7	1.01, s	1.02, s	1.02, s	1.01, s	1.01, s
8	5.82, dd (17.6, 10.6)	5.81, dd (17.8, 10.5)	5.81, dd (17.8, 10.5)	5.81, dd (17.8, 10.5)	5.81, dd (17.8, 10.5)
9a	4.90, d (10.6)	4.90, d (10.5)	4.90, d (10.5)	4.90, d (10.5)	4.90, d (10.5)
9b	4.90, d (17.6)	4.91, d (17.8)	4.91, d (17.8)	4.90, d (17.8)	4.92, d (17.8)
11a	4.58, s	4.58, s	4.58, s	4.58, s	4.57, s
11b	4.82, s	4.82, s	4.82, s	4.83, s	4.83, s
12	1.70, s	1.70, s	1.70, s	1.71, s	1.71, s
14a	4.75, d (3.3)	4.99, s	4.99, s	4.54, dddd (11.9, 3.4, 2.3, 0.9)	4.95, s
14b		5.15, s	5.15, s	4.64, dddd (11.8, 5.3, 2.1, 0.9)	5.09, s
15	6.10, d (11.0)	4.62, m	4.62, m	5.31, m	3.31, d (2.2)
16	6.55, dd (15.2, 11.0)	5.66, dd (15.6, 6.5)	5.66, dd (15.6, 6.5)	5.50, dtd (8.9, 3.7, 1.8)	2.73, dd (5.9, 2.2)
17	5.89, d (15.2)	5.90, d (15.6)	5.90, d (15.6)	5.15, dt (8.9, 1.4)	2.63, d (5.9)
19	1.35, s	1.33, s	1.33, s	1.73, s	1.35, s
20	1.35, s	1.33, s	1.33, s	1.73, s	1.39, s
22	2.07, s				

Chemical shifts (ppm) refer to CHCl_3_ (*δ*_H_ 7.26). Assignments were deduced by analysis of 1D and 2D NMR spectra.

**Table 4:** The ^1^H NMR data (600 MHz, *δ*_H_ in ppm, *J* in Hz) for compounds **11–15** in CDCl_3_.

No.	11	12	13	14	15
	*δ*_H_ Mult. (*J* Hz)	*δ*_H_ Mult. (*J* Hz)	*δ*_H_ Mult. (*J* Hz)	*δ*_H_ Mult. (*J* Hz)	*δ*_H_ Mult. (*J* Hz)
1a	1.28, m	1.10, m	3.49, dd (9.8, 5.9)	1.52, m	1.72, m
1b	1.44, m	1.39, m		1.57, m	1.83, m
2a	1.59, m	1.55, m	1.53, m	1.55, m	1.72, m
2b	1.64, m		1.70, m	1.82, m	1.76, m
3a	2.00, m	1.37, m	1.97, m	2.30, m	
3b	2.31, m	1.79, m	2.17, m	2.39, m	
4					1.79, m
5a	1.82, d (12.4)	1.25, m		3.10, ddd (8.2, 3.2, 0)	1.52, m
5b					1.83, m
6a	1.34, m	1.50, m	1.84, m	1.71, m	2.44, m
6b	1.52, m	1.50, m	2.52, dt (12.7, 2.1)	1.86, m	
7a	2.00, m	1.99, tt (9.7, 4.1)	1.84, m	2.30, m	1.64, m
7b					1.66, m
8a	1.50, m	1.24, m	1.53, m	1.72, m	2.12, ddd (14.3, 9.7, 4.0)
8b	1.51, m	1.82, m	1.70, m		2.49, ddd (14.3, 6.7, 3.5)
9a	1.28, m	1.21, m1.44, m	1.21, td (13.2, 3.4)		
9b	1.51, m		2.04, t (3.4)		
10				2.02, ddd (12.6, 10.7, 3.2)	2.27, m
11a	0.73, s	0.89, s	1.03, s	1.15, s	4.70, s
11b					4.71. s
12a	4.42, q (1.8)	1.11, s	1.59, s	4.75, s	1.18, s
12b	4.70, q (1.8)			4.87, s	
14	1.80, s	1.79, s	1.73, s	1.77, s	1.75, s
15	5.88, d (10.8)	5.87, d (10.8)	5.33, d (8.8)	5.87, d (10.7)	5.90, d (10.7)
16	6.50, dd (15.3, 10.8)	6.48, dd (15.3, 10.8)	4.26, dd (8.8, 7.8)	6.46, dd (15.3, 10.7)	6.45, dd (15.3, 10.7)
17	5.75, d (15.3)	5.75, d (15.3)	2.84, d (7.8)	5.75, d (15.3)	5.75, d (15.3)
19	1.36, s	1.35, s	1.33, s	1.36, s	1.35, s
20	1.36, s	1.35, s	1.35, s	1.36, s	1.35, s

Chemical shifts (ppm) refer to CHCl_3_ (*δ*_H_ 7.26). Assignments were deduced by analysis of 1D and 2D NMR spectra.

**Table 5:** The ^13^C NMR data (125 MHz, *δ*_C_ in ppm) for compounds **11–15** in CDCl_3_.

No.	11	12	13	14	15
	*δ*_C_ Mult.	*δ*_C_ Mult.	*δ*_C_ Mult.	*δ*_C_ Mult.	*δ*_C_ Mult.
1	42.1, CH_2_	41.2, CH_2_	78.5, CH	29.0, CH_2_	26.4, CH_2_
2	23.6, CH_2_	20.3, CH_2_	27.3, CH_2_	30.0, CH_2_	40.8, CH_2_
3	37.0, CH_2_	43.5, CH_2_	32.1, CH_2_	34.8, CH_2_	81.1, C
4	151.1, C	72.4, C	124.4, C	151.5, C	52.7, CH
5	50.0, CH	55.0, CH	133.4, C	45.3, CH	31.5, CH_2_
6	29.4, CH_2_	26.7, CH_2_	30.8, CH_2_	26.4, CH_2_	45.6, CH
7	48.0, CH	48.5, CH	47.7, CH	44.0, CH	31.2, CH_2_
8	26.7, CH_2_	26.0, CH_2_	27.3, CH_2_	40.7, CH_2_	37.0, CH_2_
9	41.3, CH_2_	44.7, CH_2_	38.9, CH_2_	81.8, C	153.4, C
10	36.1, C	34.7, C	39.6, C	48.6, CH	48.4, CH
11	16.5, CH_3_	18.9, CH_3_	17.5, CH_3_	24.1, CH_3_	107.2, CH_2_
12	105.6, CH_2_	22.9, CH_3_	19.2, CH_3_	109.3, CH_2_	24.0, CH_3_
13	144.1, C	143.8, C	145.9, C	143.2, C	143.9, C
14	15.4, CH_3_	15.5, CH_3_	15.9, CH_3_	16.1, CH_3_	14.7, CH_3_
15	122.4, CH	122.5, CH	120.9, CH	122.8, CH	123.0, CH
16	123.4, CH	123.3, CH	67.9, CH	123.4, CH	123.2, CH
17	139.3, CH	139.3, CH	67.6, CH	139.5, CH	139.5, CH
18	71.1, C	71.1, C	60.0, C	71.1, C	71.1, C
19	30.1, CH_3_	30.1, CH_3_	19.7, CH_3_	30.1, CH_3_	30.1, CH_3_
20	30.1, CH_3_	30.1, CH_3_	25.1, CH_3_	30.1, CH_3_	30.1, CH_3_

Chemical shifts (ppm) refer to CHCl_3_ (*δ*_C_ 77.2). Assignments were deduced by analysis of 1D and 2D NMR spectra.

**Table 6:** Antibacterial activities on fish pathogenic bacteria of compounds **1**–**6**, **10,11**, **16**–**18**.

Compd.	MIC (μg/mL)				
	*Streptococcus parauberis*	*Lactococcus garvieae*	*Streptococcus* *parauberis*	*Photobacterium* *damselae*	*Aeromonas* *salmonicida*
	FP KSP28	MP5245	SP0F3K	FP2244	AS42
**1**	14.4	28.8	7.2	7.2	NA
**2**	7.2	NA	28.8	14.4	NA
**3**	7.2	NA	NA	14.4	28.8
**4**	7.2	0.225	NA	28.8	NA
**5**	7.2	28.8	NA	28.8	NA
**6**	17.3	NA	17.3	17.3	NA
**10**	15.1	NA	NA	NA	NA
**11**	0.9	14.4	NA	7.2	28.8
**16**	7.2	14.4	3.6	7.2	NA
**17**	NA	NA	NA	NA	NA
**18**	7.6	NA	NA	15.2	NA
Tetr	3.01	0.38	>24.05	0.02	6.11
Oxy	1.55	0.19	12.42	0.02	0.39
Lev	1.24	0.62	1.24	0.02	0.31
AMP	4.64	0.58	0.58	0.02	>18.57

Tetr = Tetracycline, Oxy = Oxytetracycline hydrochloride, Lev = Levofloxacin hydrochloride. and AMP = Ampicillin sodium, used as positive control; NA = not active at 30 μg/mL; significant (MIC ≤ 10 μg/mL), moderate (10 < MIC ≤ 100 μg/mL), and low or negligible (MIC > 100 μg/mL) [30,31].

## Data Availability

Data are contained within the article or Supplementary Materials.

## Supplementary Materials

The following supporting information can be downloaded at: https://www.mdpi.com/article/10.3390/md21060362/s1, Figures S1–S110: NMR, HRESIMS, HREIMS, UV, CD, and IR data of compounds **1**–**15**; Figures S111–S116: Structure of studied isomers of compound **9** and **10**, Averaged isotropic magnetic shielding constants (σ) of studied isomers and experimental ^1^H and ^13^C data of **9** and **10**, and the DP4+ results were obtained using experimental data of compounds **9** versus isomers **9a**–**9b** and **10** versus isomers **10a**–**10d**.
