# Supplementary material for: New Antibacterial Diterpenoids from the South China Sea Soft Coral Klyxum molle
Source: Mar Drugs. 2023 Jun 16;21(6):362. doi: 10.3390/md21060362 (PMC10304424; doi:10.3390/md21060362)
Supplement: Supplementary file 1 [file marinedrugs-21-00362-s001.zip › marinedrugs-2430236-supplementary.pdf]

Table of contents

Figure S1. ^{1}H NMR spectrum of **1** (600MHz, $CDCl_3$)
Figure S2. ^{13}C NMR spectrum of **1** (125 MHz, $CDCl_3$)
Figure S3. HSQC spectrum of **1** (600 MHz, $CDCl_3$)
Figure S4. HMBC spectrum of **1** (600 MHz, $CDCl_3$)
Figure S5. ^{1}H–^{1}H COSY spectrum of **1** (600 MHz, $CDCl_3$)
Figure S6. NOESY spectrum of **1** (600 MHz, $CDCl_3$)
Figure S7. HRESIMS spectrum of **1**
Figure S8. UV and CD spectrum of **1**
Figure S9. IR spectrum of **1**
Figure S10. ^{1}H NMR spectrum of **2** (600MHz, $CDCl_3$)
Figure S11. ^{13}C NMR spectrum of **2** (125 MHz, $CDCl_3$)
Figure S12. UV and CD spectrum of **2**
Figure S13. ^{1}H NMR spectrum of **3** (600MHz, $CDCl_3$)
Figure S14. ^{13}C NMR spectrum of **3** (125 MHz, $CDCl_3$)
Figure S15. UV and CD spectrum of **3**
Figure S16. ^{1}H NMR spectrum of **4** (600MHz, $CDCl_3$)
Figure S17. ^{13}C NMR spectrum of **4** (125 MHz, $CDCl_3$)
Figure S18. ^{1}H NMR spectrum of **5** (600MHz, $CDCl_3$)
Figure S19. ^{13}C NMR spectrum of **5** (125 MHz, $CDCl_3$)
Figure S20. ^{13}C NMR spectrum for mixture of **4** and **5** (125 MHz, $CDCl_3$)
Figure S21. HSQC spectrum for mixture of **4** and **5** (600 MHz, $CDCl_3$)
Figure S22. HMBC spectrum for mixture of **4** and **5** (600 MHz, $CDCl_3$)
Figure S23. ^{1}H–^{1}H COSY spectrum for mixture of **4** and **5** (600 MHz, $CDCl_3$)
Figure S24. NOESY spectrum for mixture of **4** and **5** (600 MHz, $CDCl_3$)
Figure S25. HREIMS spectrum for mixture of **4** and **5**
Figure S26. UV and CD spectrum of **4**
Figure S27. UV and CD spectrum of **5**
Figure S28. IR spectrum for mixture of **4** and **5**
Figure S29. ^{1}H NMR spectrum of **6** (600MHz, $CDCl_3$)
Figure S30. ^{13}C NMR spectrum of **6** (125 MHz, $CDCl_3$)
Figure S31. HSQC spectrum of **6** (600 MHz, $CDCl_3$)
Figure S32. HMBC spectrum of **6** (600 MHz, $CDCl_3$)
Figure S33. ^{1}H–^{1}H COSY spectrum of **6** (600 MHz, $CDCl_3$)
Figure S34. NOESY spectrum of **6** (600 MHz, $CDCl_3$)
Figure S35. HRESIMS spectrum of **6**
Figure S36. UV and CD spectrum of **6**
Figure S37. IR spectrum of **6**
Figure S38. ^{1}H NMR spectrum of **7** (600MHz, $CDCl_3$)
Figure S39. ^{13}C NMR spectrum of **7** (125 MHz, $CDCl_3$)
Figure S40. ^{1}H NMR spectrum of **8** (600MHz, $CDCl_3$)
Figure S41. ^{13}C NMR spectrum of **8** (125 MHz, $CDCl_3$)
Figure S42. ^{13}C NMR spectrum for mixture of **7** and **8** (125 MHz, $CDCl_3$)

Figure S43. HSQC spectrum for mixture of **7** and **8** (600 MHz, $CDCl_3$)
Figure S44. HMBC spectrum for mixture of **7** and **8** (600 MHz, $CDCl_3$)
Figure S45. $^1H–^1H$ COSY spectrum for mixture of **7** and **8** (600 MHz, $CDCl_3$)
Figure S46. NOESY spectrum for mixture of **7** and **8** (600 MHz, $CDCl_3$)
Figure S47. HRESIMS spectrum for mixture of **7** and **8**
Figure S48. UV and CD spectrum of **7**
Figure S49. UV and CD spectrum of **8**
Figure S50. IR spectrum for mixture of **7** and **8**
Figure S51. ^1H NMR spectrum of **9** (600MHz, $CDCl_3$)
Figure S52. ^{13}C NMR spectrum of **9** (125 MHz, $CDCl_3$)
Figure S53. HSQC spectrum of **9** (600 MHz, $CDCl_3$)
Figure S54. HMBC spectrum of **9** (600 MHz, $CDCl_3$)
Figure S55. $^1H–^1H$ COSY spectrum of **9** (600 MHz, $CDCl_3$)
Figure S56. NOESY spectrum of **9** (600 MHz, $CDCl_3$)
Figure S57. HREIMS spectrum of **9**
Figure S58. IR spectrum of **9**
Figure S59. ^1H NMR spectrum of **10** (600MHz, $CDCl_3$)
Figure S60. ^{13}C NMR spectrum of **10** (125 MHz, $CDCl_3$)
Figure S61. HSQC spectrum of **10** (600 MHz, $CDCl_3$)
Figure S62. HMBC spectrum of **10** (600 MHz, $CDCl_3$)
Figure S63. $^1H–^1H$ COSY spectrum of **10** (600 MHz, $CDCl_3$)
Figure S64. NOESY spectrum of **10** (600 MHz, $CDCl_3$)
Figure S65. HRESIMS spectrum of **10**
Figure S66. IR spectrum of **10**
Figure S67. ^1H NMR spectrum of **11** (600MHz, $CDCl_3$)
Figure S68. ^{13}C NMR spectrum of **11** (125 MHz, $CDCl_3$)
Figure S69. HSQC spectrum of **11** (600 MHz, $CDCl_3$)
Figure S70. HMBC spectrum of **11** (600 MHz, $CDCl_3$)
Figure S71. $^1H–^1H$ COSY spectrum of **11** (600 MHz, $CDCl_3$)
Figure S72. NOESY spectrum of **11** (600 MHz, $CDCl_3$)
Figure S73. HREIMS spectrum of **11**
Figure S74. UV and CD spectrum of **11**
Figure S75. IR spectrum of **11**
Figure S76. ^1H NMR spectrum of **12** (600MHz, $CDCl_3$)
Figure S77. ^{13}C NMR spectrum of **12** (125 MHz, $CDCl_3$)
Figure S78. HSQC spectrum of **12** (600 MHz, $CDCl_3$)
Figure S79. HMBC spectrum of **12** (600 MHz, $CDCl_3$)
Figure S80. $^1H–^1H$ COSY spectrum of **12** (600 MHz, $CDCl_3$)
Figure S81. NOESY spectrum of **12** (600 MHz, $CDCl_3$)
Figure S82. HREIMS spectrum of **12**
Figure S83. UV and CD spectrum of **12**
Figure S84. IR spectrum of **12**
Figure S85. ^1H NMR spectrum of **13** (600MHz, $CDCl_3$)
Figure S86. ^{13}C NMR spectrum of **13** (125 MHz, $CDCl_3$)

Figure S87. HSQC spectrum of **13** (600 MHz, $CDCl_3$)
Figure S88. HMBC spectrum of **13** (600 MHz, $CDCl_3$)
Figure S89. ^{1}H–^{1}H COSY spectrum of **13** (600 MHz, $CDCl_3$)
Figure S90. NOESY spectrum of **13** (600 MHz, $CDCl_3$)
Figure S91. HREIMS spectrum of **13**
Figure S92. IR spectrum of **13**
Figure S93. ^{1}H NMR spectrum of **14** (600MHz, $CDCl_3$)
Figure S94. ^{13}C NMR spectrum of **14** (125 MHz, $CDCl_3$)
Figure S95. HSQC spectrum of **14** (600 MHz, $CDCl_3$)
Figure S96. HMBC spectrum of **14** (600 MHz, $CDCl_3$)
Figure S97. ^{1}H–^{1}H COSY spectrum of **14** (600 MHz, $CDCl_3$)
Figure S98. NOESY spectrum of **14** (600 MHz, $CDCl_3$)
Figure S99. HREIMS spectrum of **14**
Figure S100. UV and CD spectrum of **14**
Figure S101. IR spectrum of **14**
Figure S102. ^{1}H NMR spectrum of **15** (600MHz, $CDCl_3$)
Figure S103. ^{13}C NMR spectrum of **15** (125 MHz, $CDCl_3$)
Figure S104. HSQC spectrum of **15** (600 MHz, $CDCl_3$)
Figure S105. HMBC spectrum of **15** (600 MHz, $CDCl_3$)
Figure S106. ^{1}H–^{1}H COSY spectrum of **15** (600 MHz, $CDCl_3$)
Figure S107. NOESY spectrum of **15** (600 MHz, $CDCl_3$)
Figure S108. HREIMS spectrum of **15**
Figure S109. UV and CD spectrum of **15**
Figure S110. IR spectrum of **15**
Figure S111. Structure of studied isomers of compound **9**
Figure S112. Averaged isotropic magnetic shielding constants (σ) of studied isomers and experimental ^{1}H and ^{13}C data of **9**
Figure S113. DP4+ results obtained using experimental ^{1}H and ^{13}C data of compound **9** versus isomers **9a** and **9b**
Figure S114. Structure of studied isomers of compound **10**
Figure S115. Averaged isotropic magnetic shielding constants (σ) of studied isomers and experimental ^{1}H and ^{13}C data of **10**
Figure S116. DP4+ results obtained using experimental ^{1}H and ^{13}C data of compound **10** versus isomers **10a**, **10b, 10c, 10d**

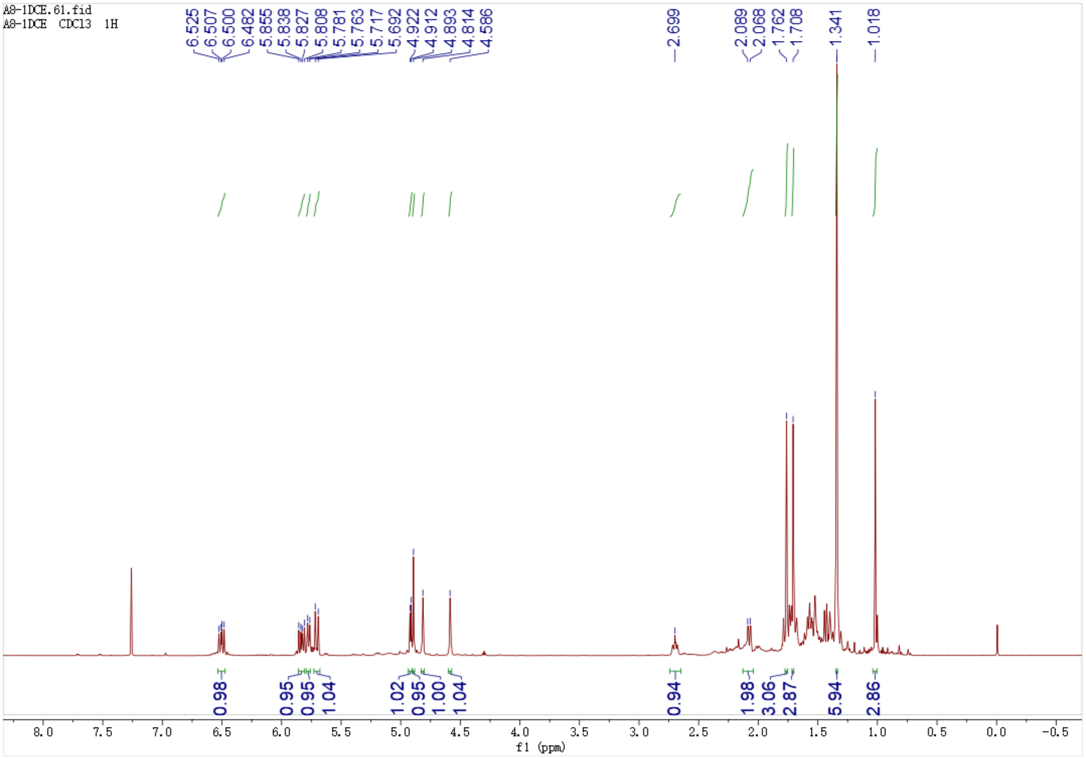

Figure S1. ^{1}H NMR spectrum of **1** (600MHz, $CDCl_3$)

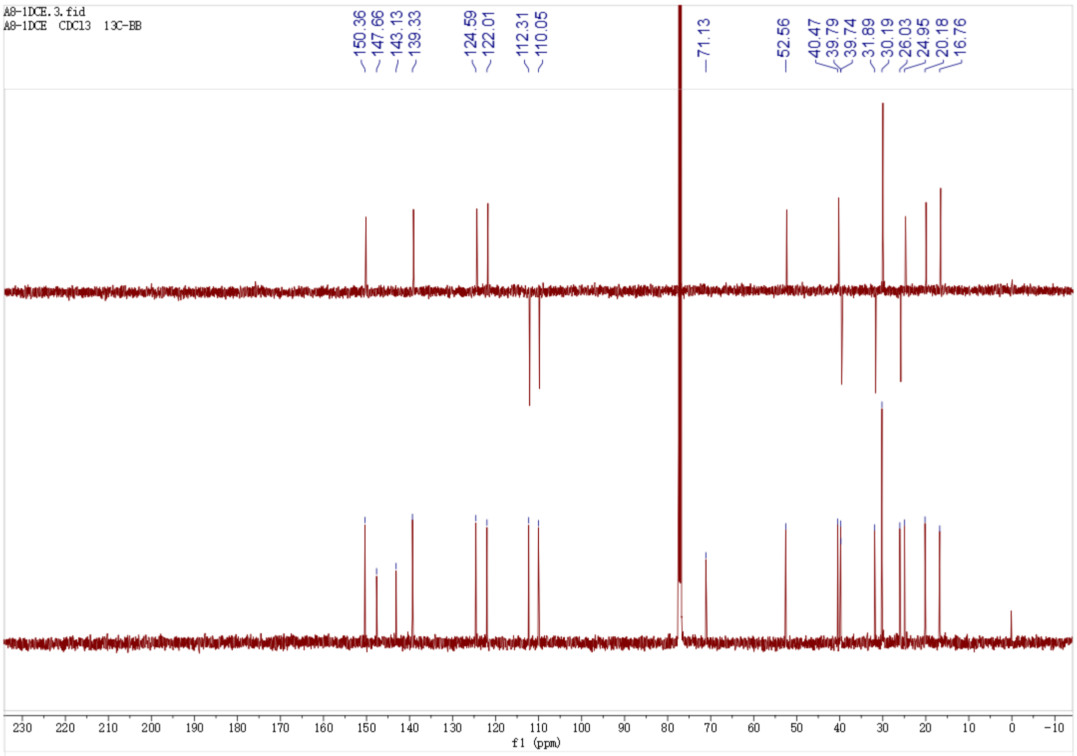

Figure S2. ^{13}C NMR spectrum of **1** (125 MHz, $CDCl_3$)

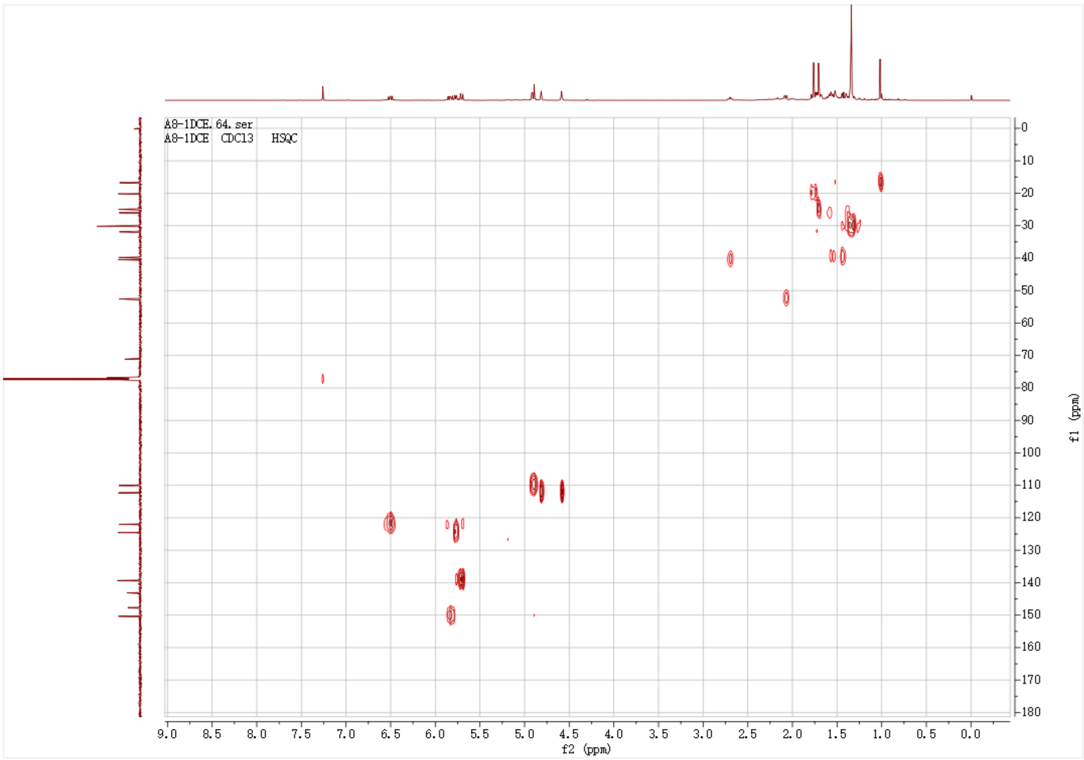

Figure S3. HSQC spectrum of **1** (600 MHz, $CDCl_3$)

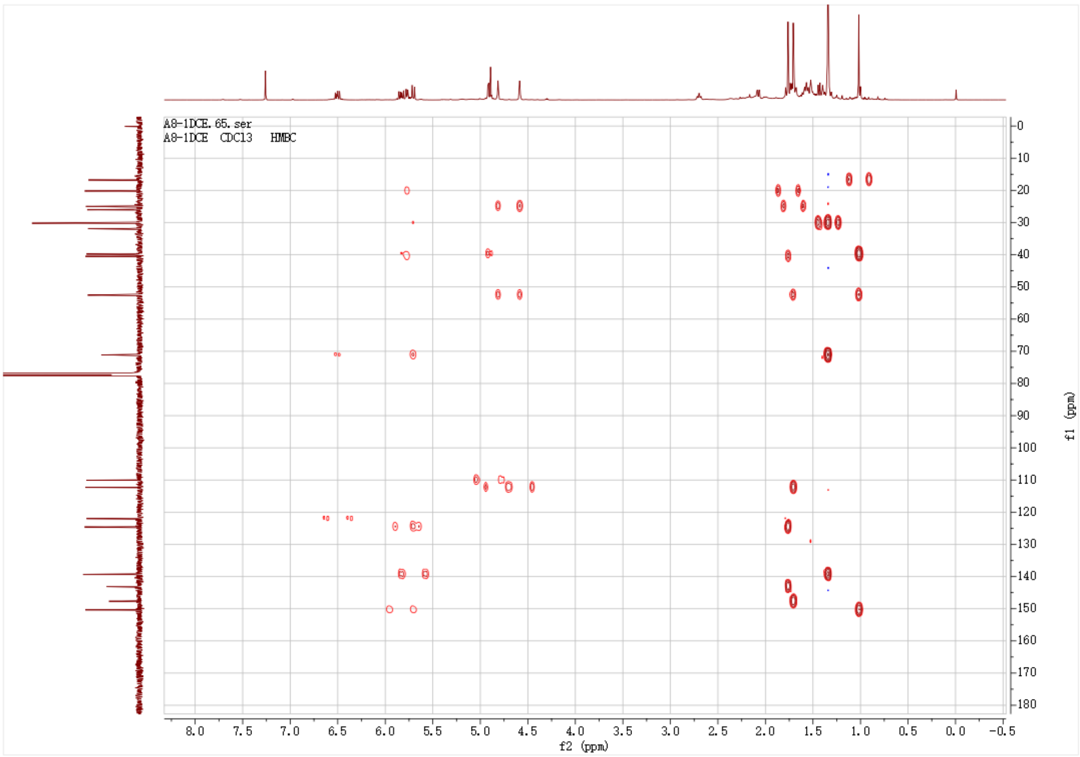

Figure S4. HMBC spectrum of **1** (600 MHz, $CDCl_3$)

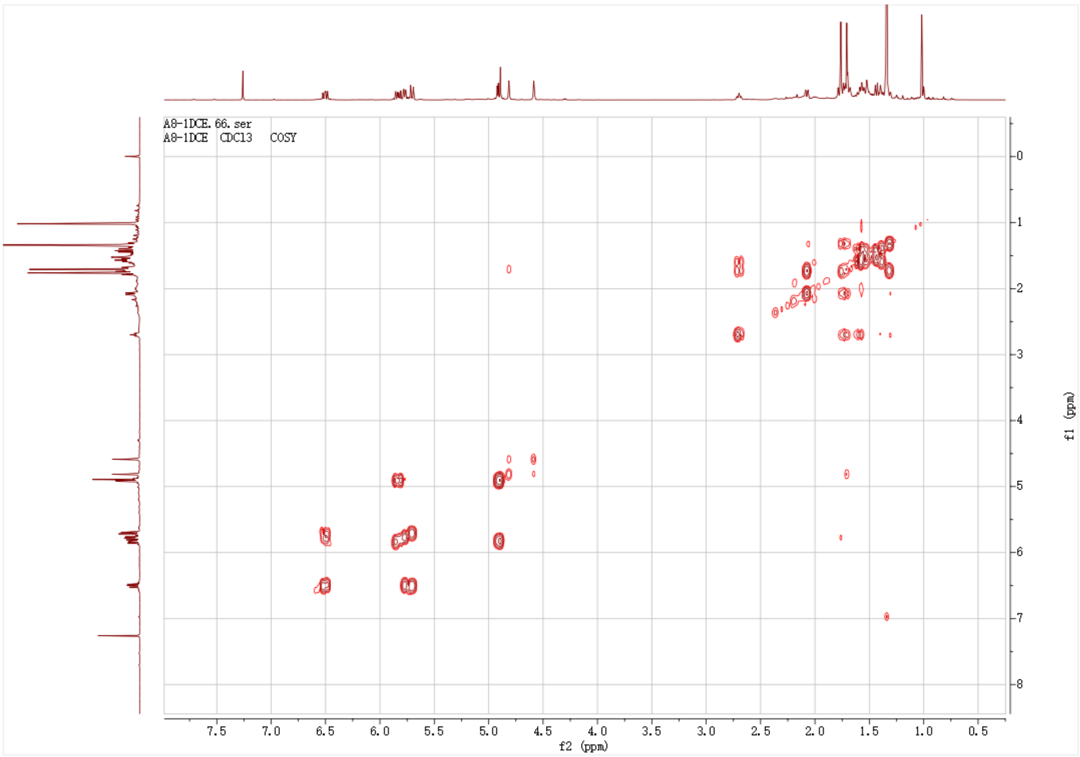

Figure S5. ^{1}H–^{1}H COSY spectrum of **1** (600 MHz, $CDCl_3$)

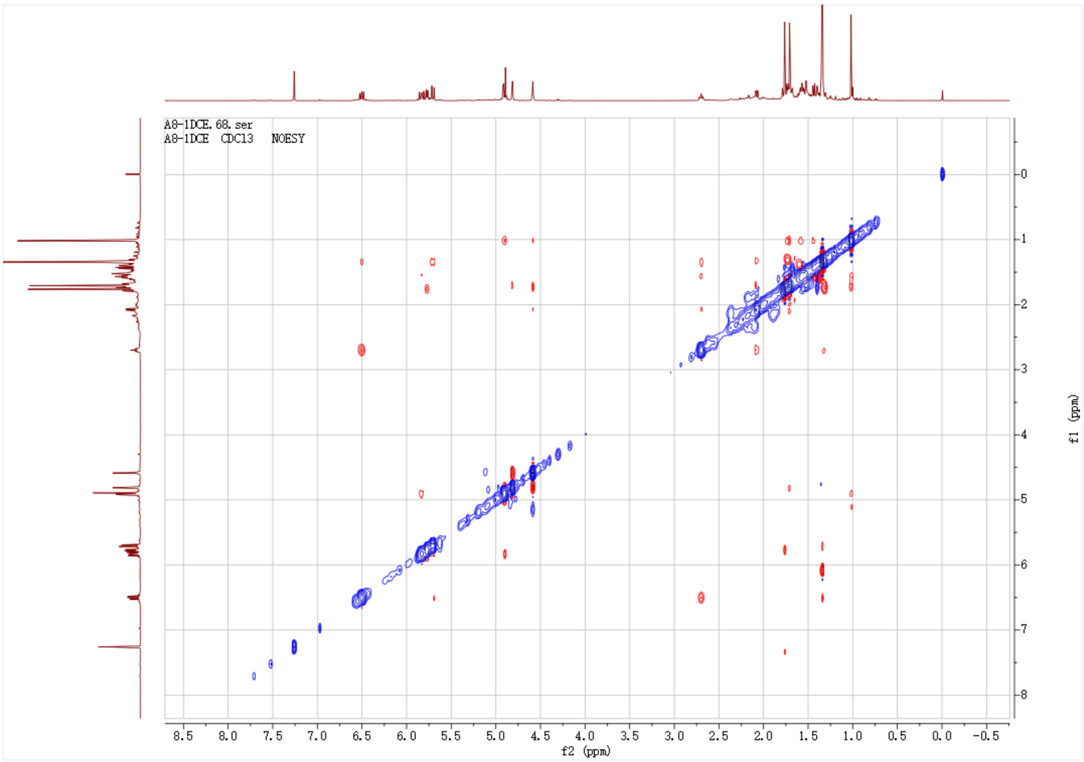

Figure S6. NOESY spectrum of **1** (600 MHz, $CDCl_3$)

Qualitative Analysis Report

**Data Filename**	ESIH202103095.d	**Sample Name**	A8-1DCE
**Sample ID**		**Position**	P2-A3
**Instrument Name**	Agilent G6520 Q-TOF	**Acq Method**	20160322_MS_ESIH_POS_1min.m
**Acquired Time**	6/18/2021 16:27:43	**IRM Calibration Status**	Success
**DA Method**	small molecular data analysis method.m	**Comment**	ESIH by zhuzhenyun

User Spectra

Fragmentor Voltage	Collision Energy	Ionization Mode
175	0	ESI

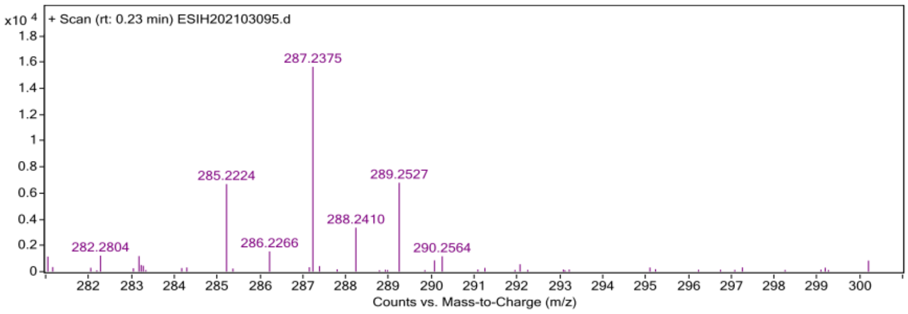

Formula Calculator Results

m/z	Calc m/z	Diff (mDa)	Diff (ppm)	Ion Formula	Ion
289.2527	289.2526	-0.07	-0.24	C20 H33 O	(M+H)+

--- End Of Report ---

Figure S7. HRESIMS spectrum of **1**

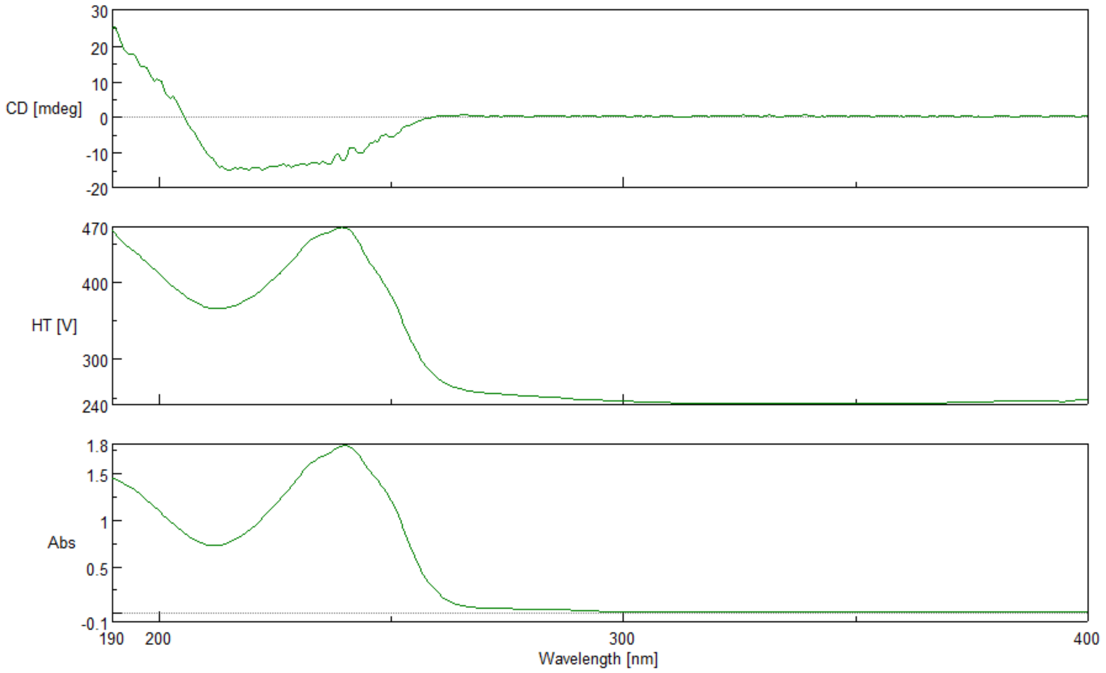

Figure S8. UV and CD spectrum of **1**

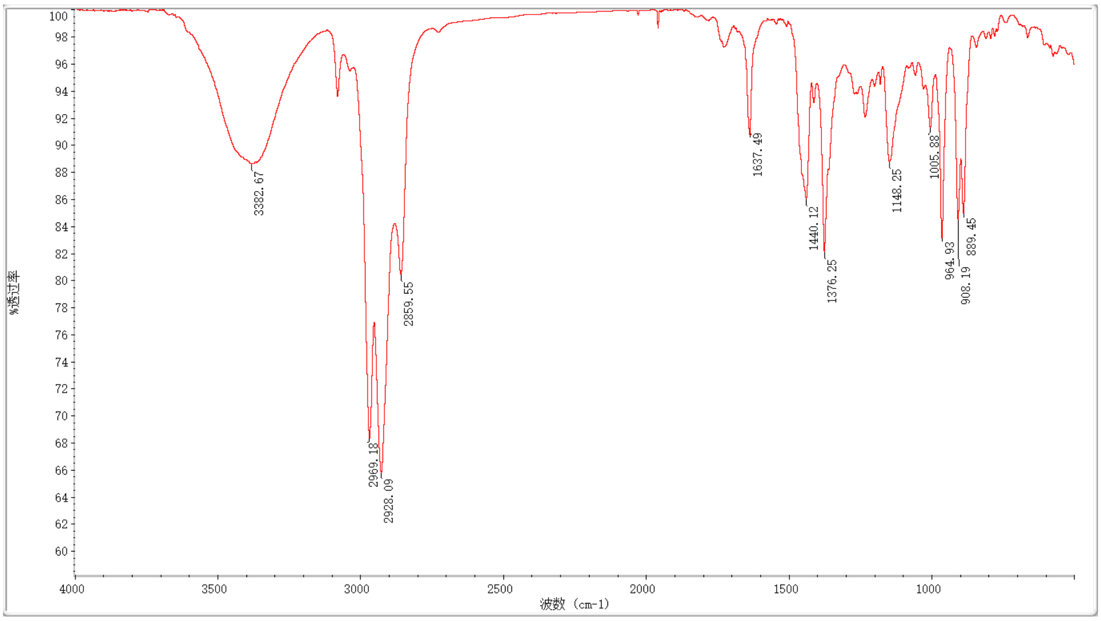

Figure S9. IR spectrum of **1**

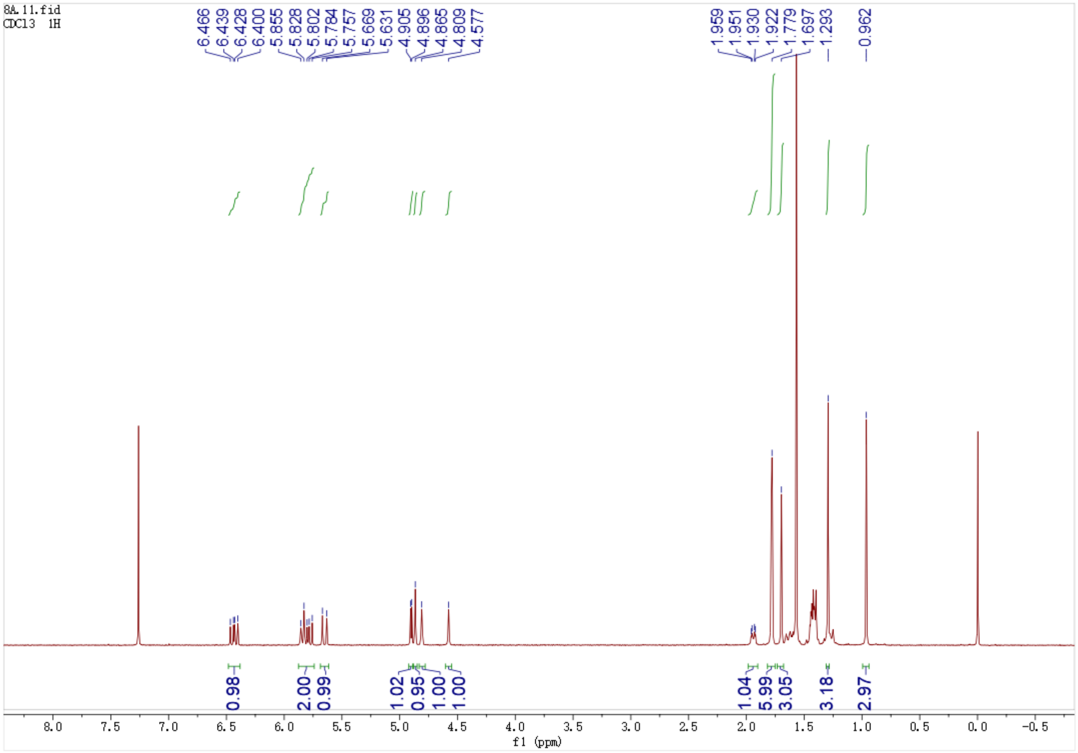

Figure S10. ^{1}H NMR spectrum of **2** (600MHz, $CDCl_3$)

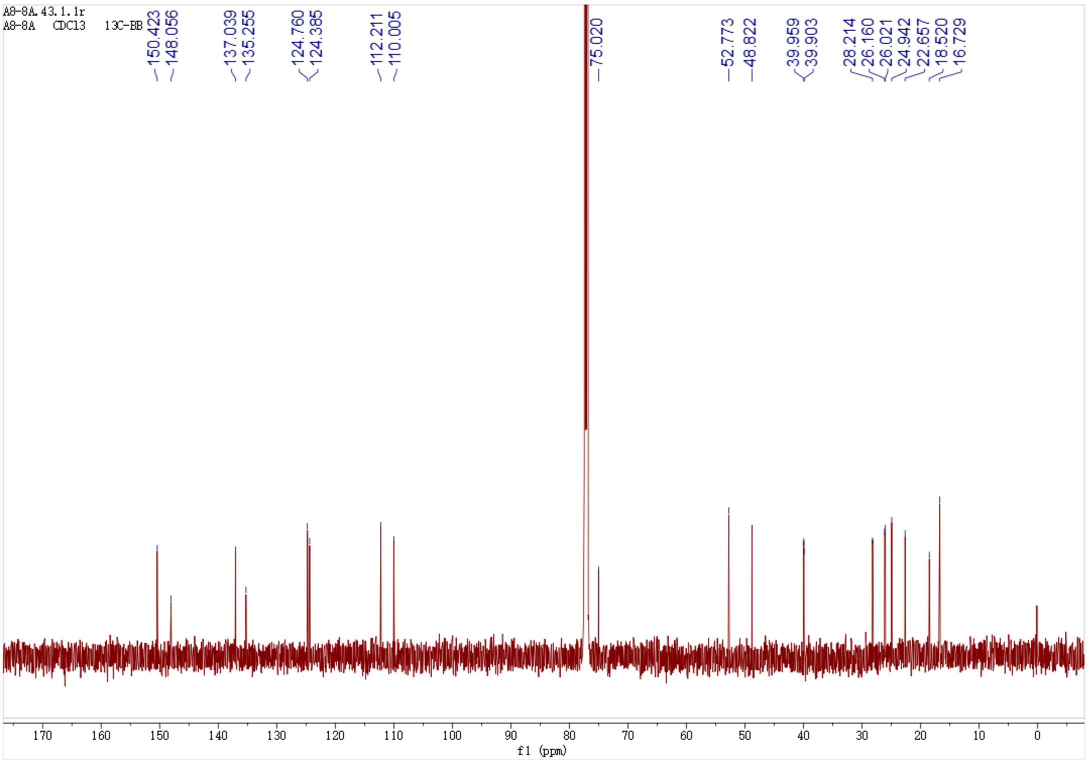

Figure S11. ^{13}C NMR spectrum of **2** (125 MHz, $CDCl_3$)

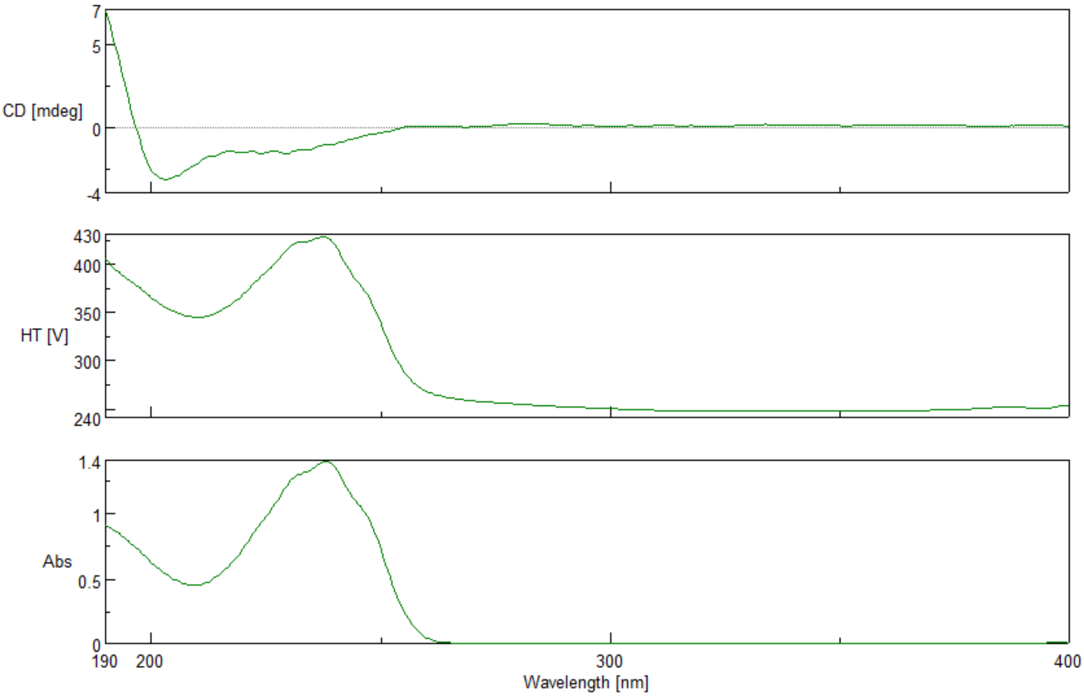

Figure S12. UV and CD spectrum of **2**

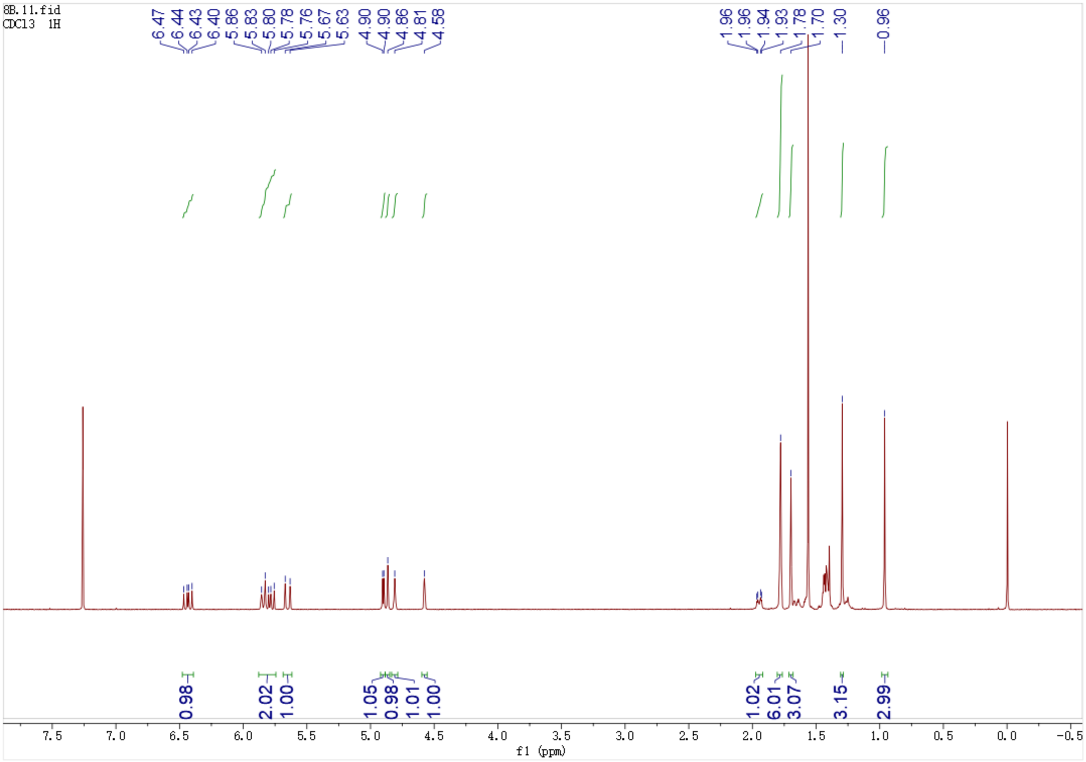

Figure S13. ^{1}H NMR spectrum of **3** (600MHz, CDCl3)

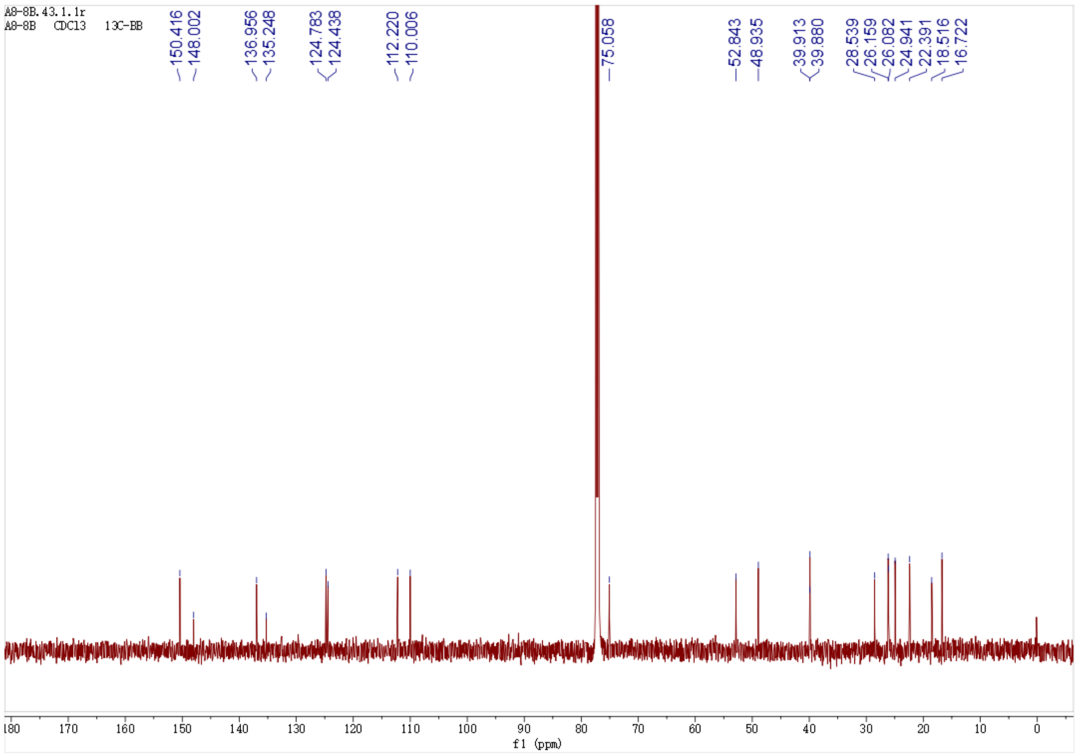

Figure S14. ^{13}C NMR spectrum of **3** (125 MHz, CDCl3)

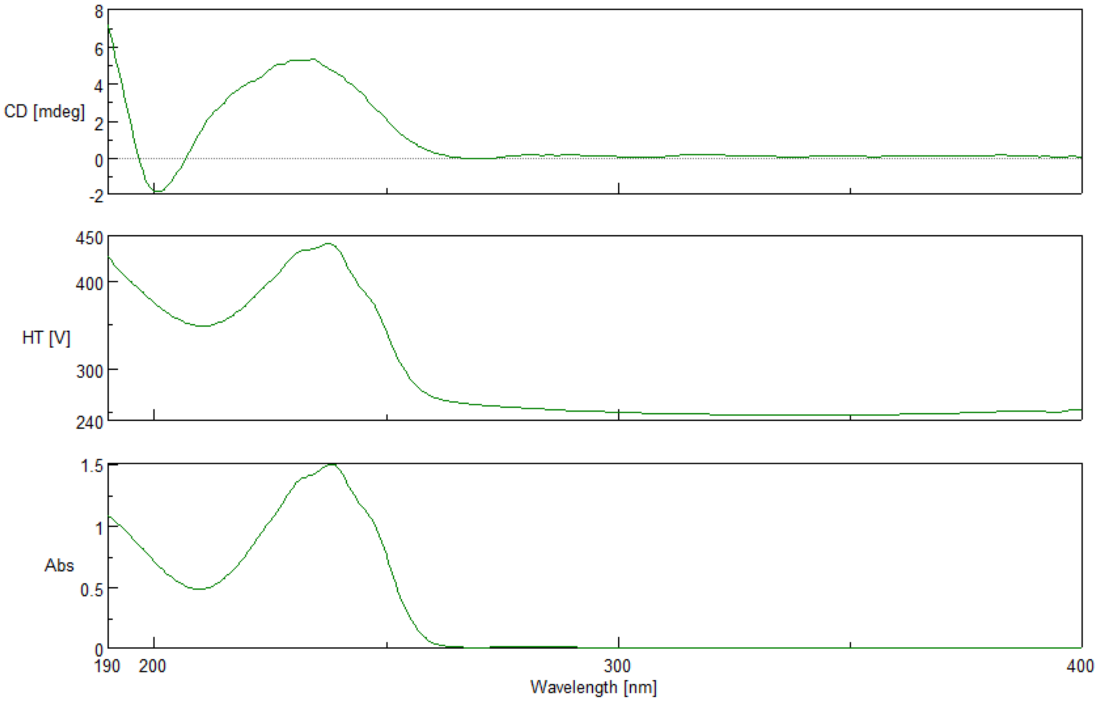

Figure S15. UV and CD spectrum of **3**

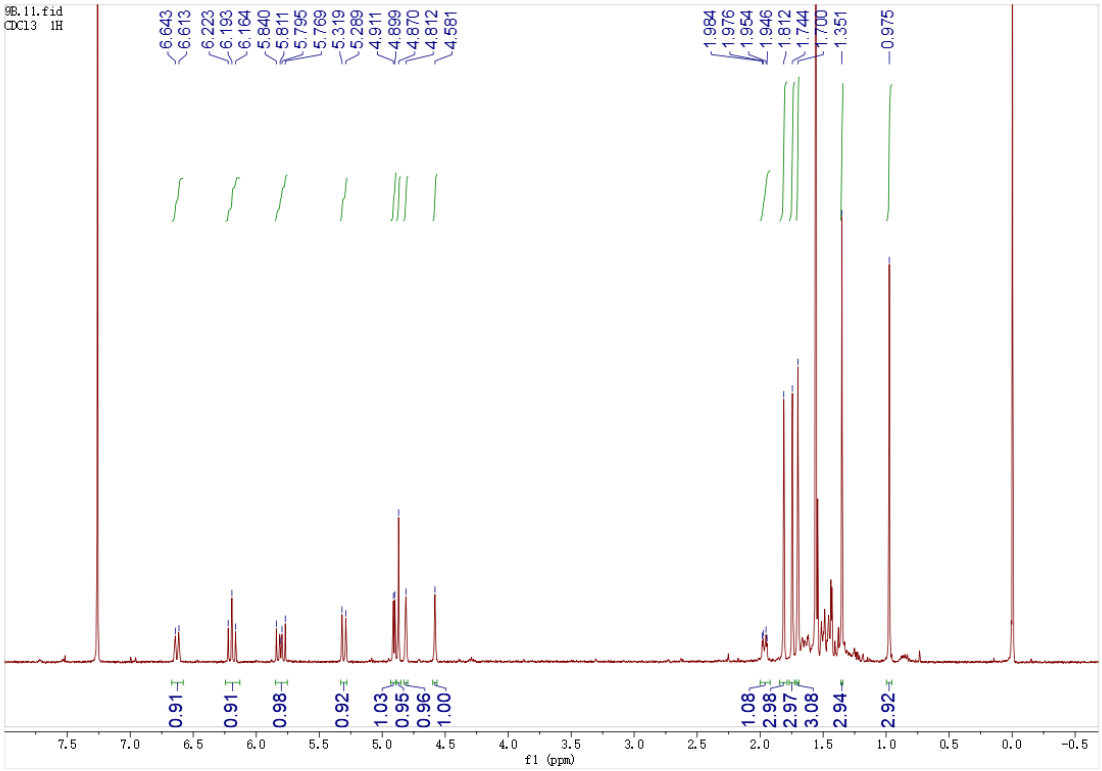

Figure S16. ¹H NMR spectrum of **4** (600MHz, $CDCl_3$)

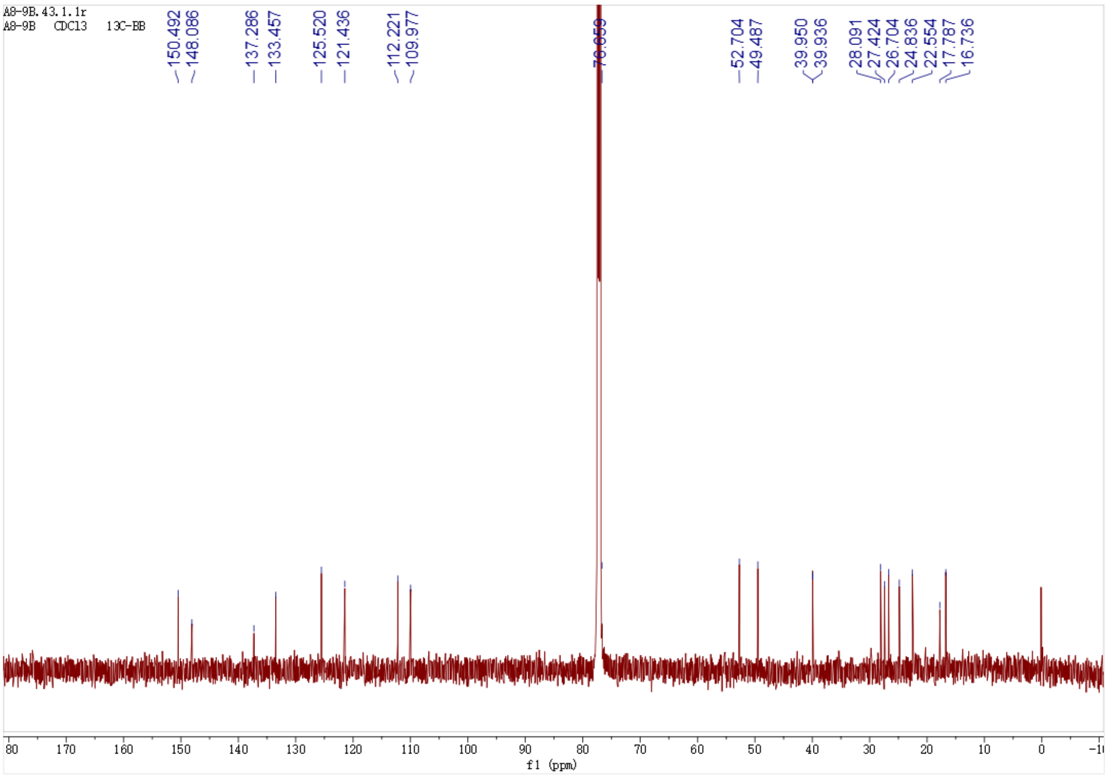

Figure S17. ^{13}C NMR spectrum of **4** (125 MHz, CDCl3)

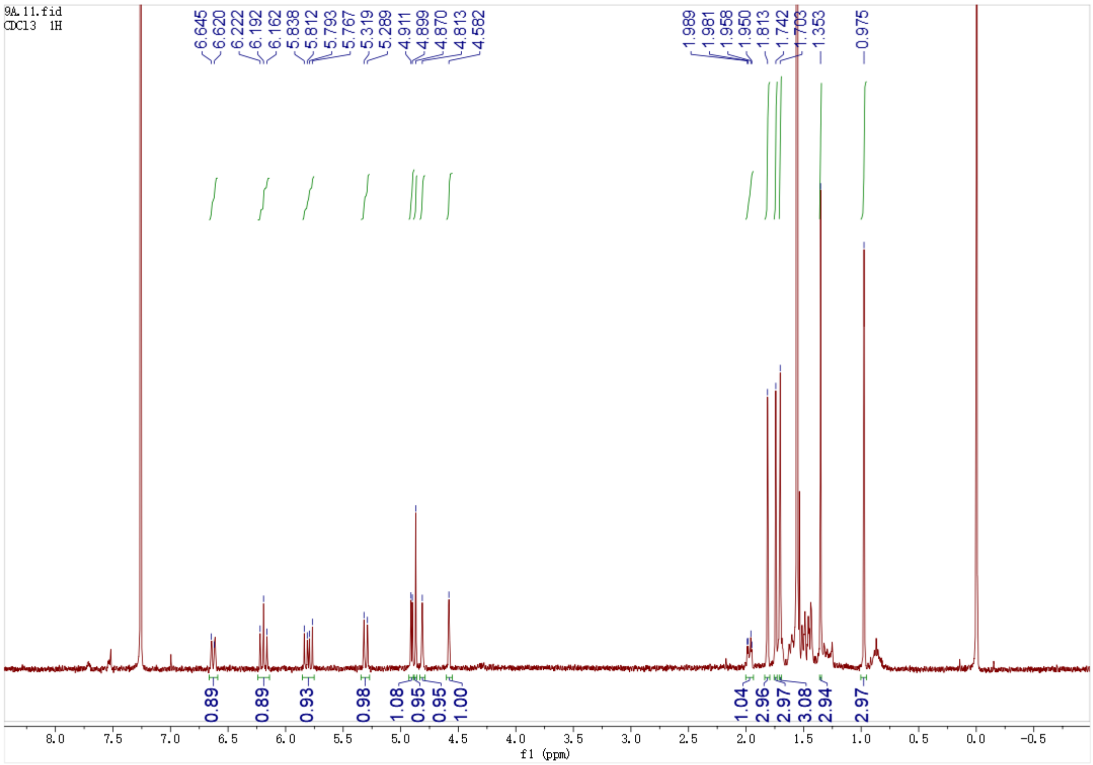

Figure S18. ^{1}H NMR spectrum of **5** (600MHz, CDCl3)

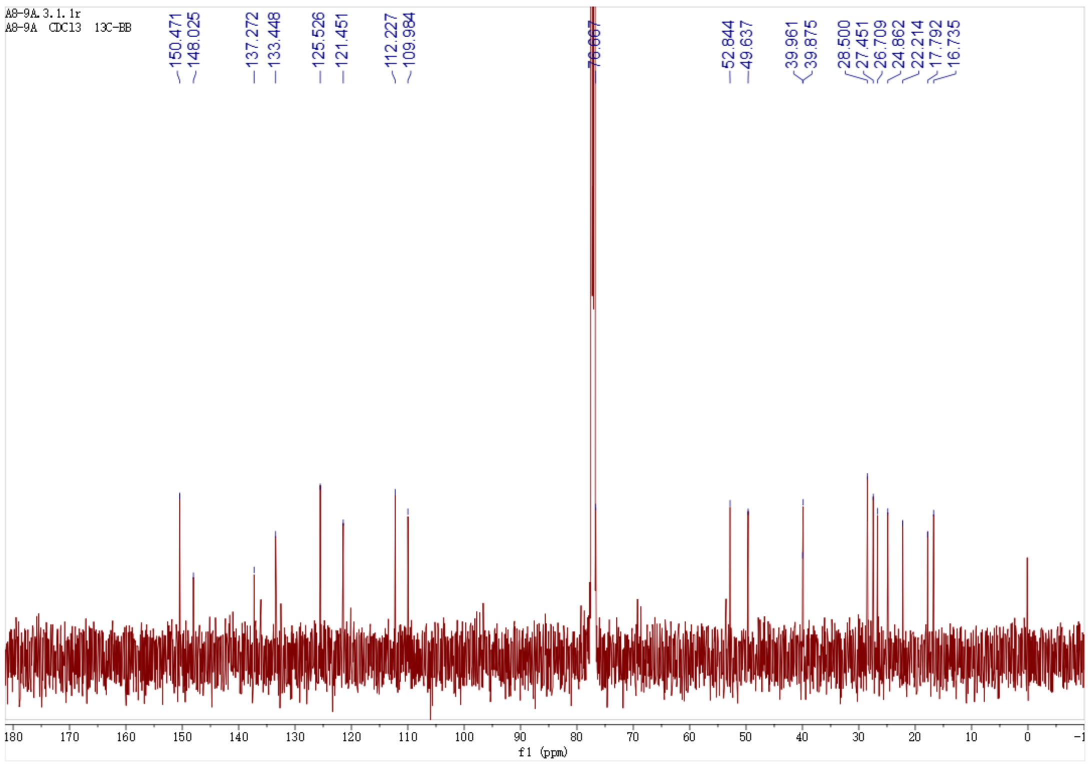

Figure S19. ^{13}C NMR spectrum of **5** (125 MHz, CDCl3)

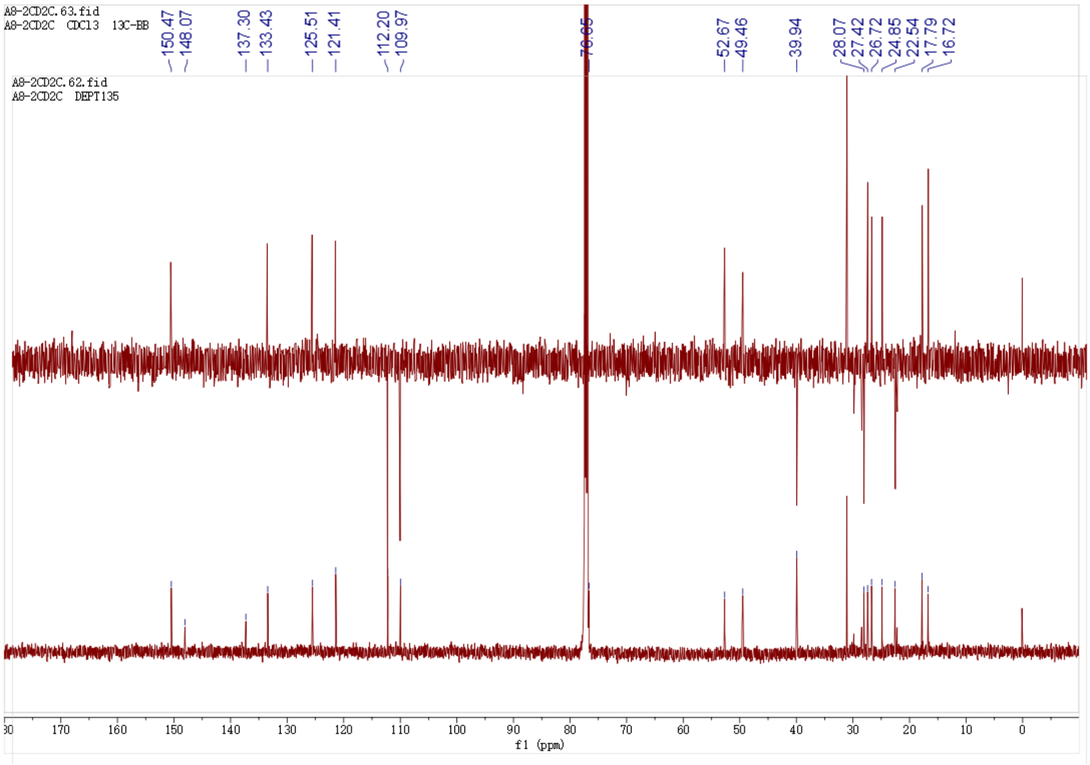

Figure S20. ^{13}C NMR spectrum for mixture of **4** and **5** (125 MHz, CDCl3)

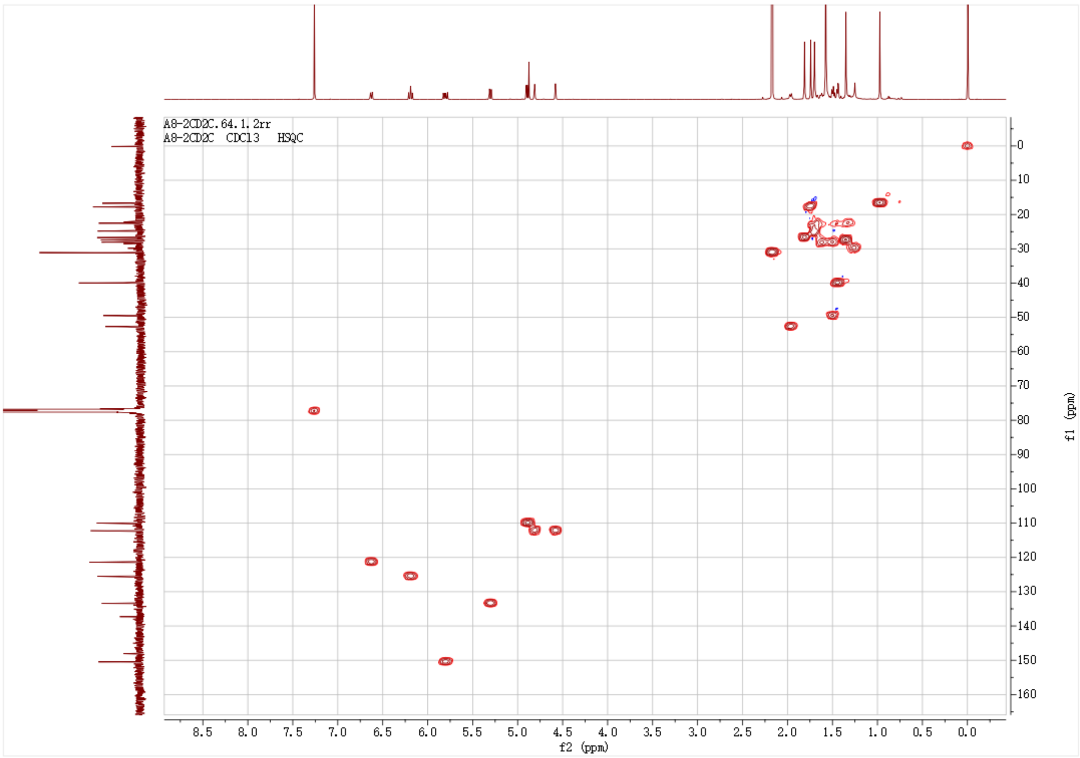

Figure S21. HSQC spectrum for mixture of **4** and **5** (600 MHz, $CDCl_3$)

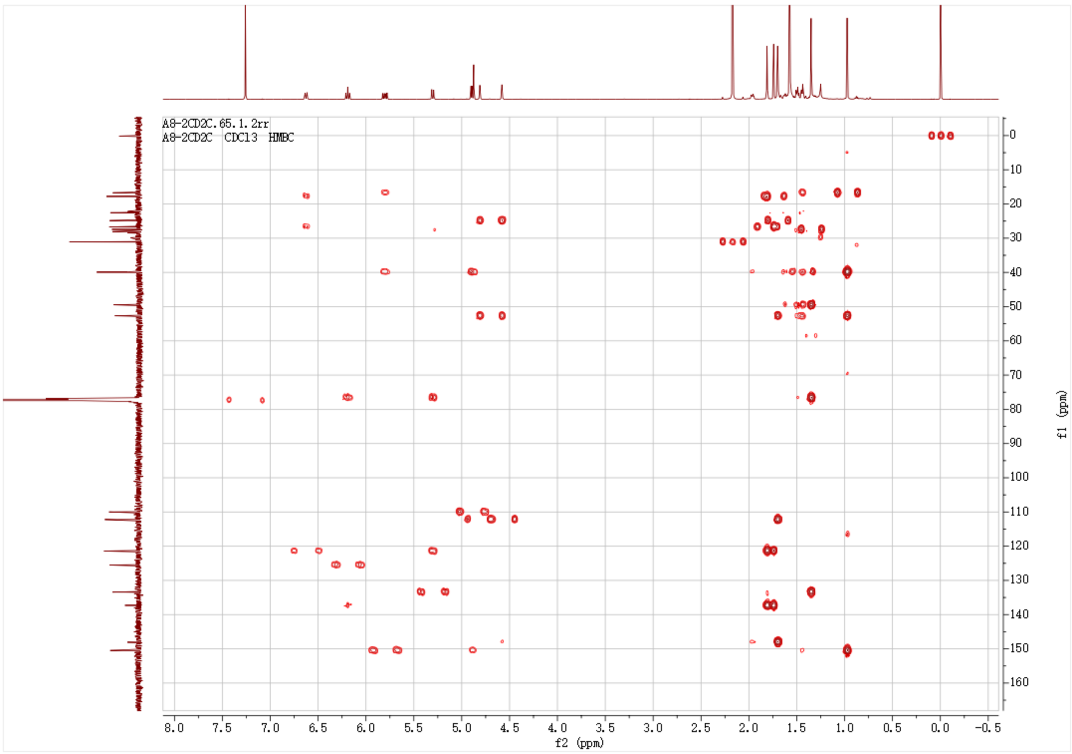

Figure S22. HMBC spectrum for mixture of **4** and **5** (600 MHz, $CDCl_3$)

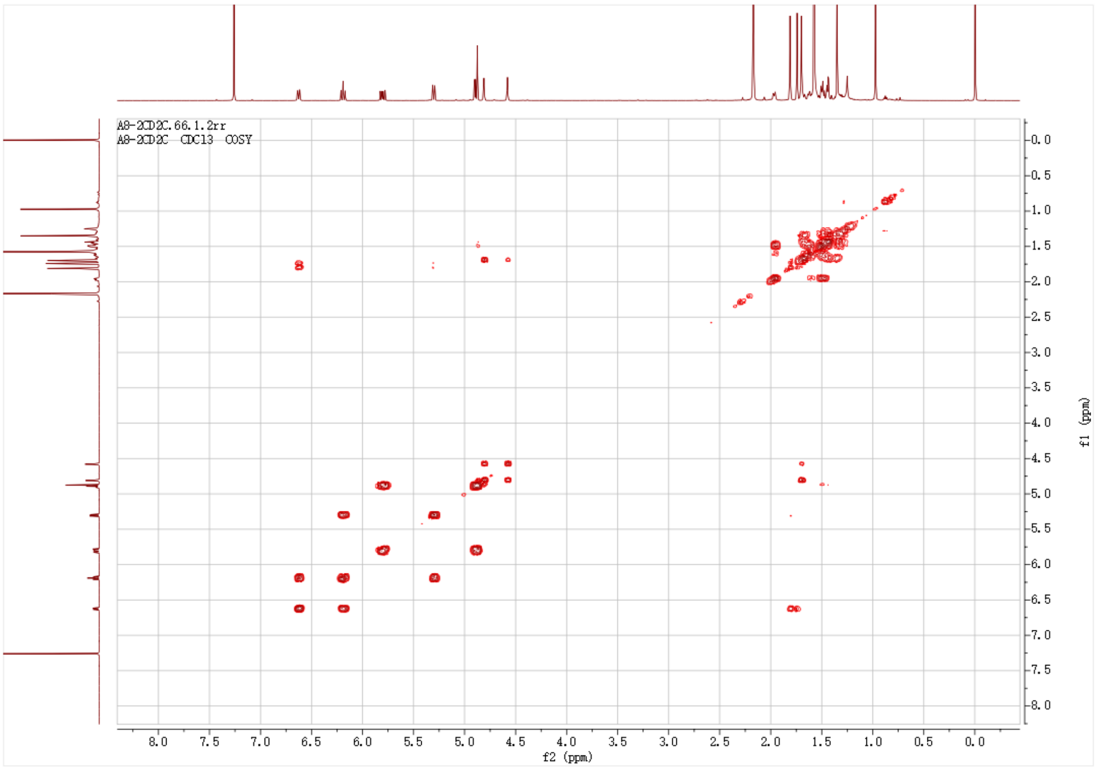

Figure S23. ^{1}H–^{1}H COSY spectrum for mixture of **4** and **5** (600 MHz, $CDCl_3$)

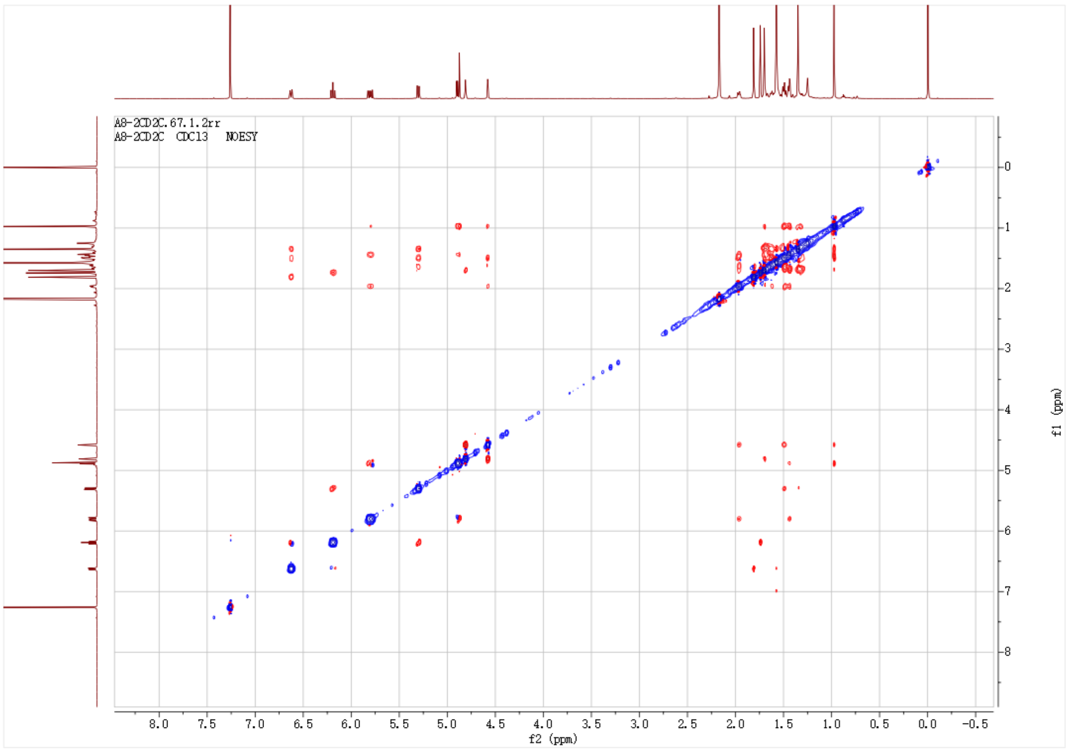

Figure S24. NOESY spectrum for mixture of **4** and **5** (600 MHz, $CDCl_3$)

D:\data\2021\EI202101585_A8-2CD2C -c1 9/13/2021 2:09:38 PM

EI202101585_A8-2CD2C -c1#6 RT: 1.04
T: + c EI Full ms [49.50-800.50]
m/z= 48-803

m/z	Intensity	Relative	Theo. Mass	Delta (mmu)	RDB equiv.	Composition
245.1899	203136.0	0.22	245.1900	-0.09	5.5	$C_{17}H_{25}O_{1}$
245.2261	242856.0	0.26	245.2264	-0.23	4.5	$C_{18}H_{29}$
246.1945	95360.0	0.10	246.1978	-3.31	5.0	$C_{17}H_{26}O_{1}$
255.2110	361052.0	0.39	255.2107	0.26	6.5	$C_{19}H_{27}$
256.2154	75098.0	0.08	256.2186	-3.13	6.0	$C_{19}H_{28}$
270.2346	581491.0	0.62	270.2342	0.44	6.0	$C_{20}H_{30}$
271.2387	171166.0	0.18	271.2420	-3.30	5.5	$C_{20}H_{31}$
273.2214	335740.0	0.36	273.2213	0.11	5.5	$C_{19}H_{29}O_{1}$
286.2296	77414.0	0.08	286.2291	0.47	6.0	$C_{20}H_{30}O_{1}$
288.2454	176988.0	0.19	288.2448	0.60	5.0	$C_{20}H_{32}O_{1}$

Figure S25. HREIMS spectrum for mixture of **4** and **5**

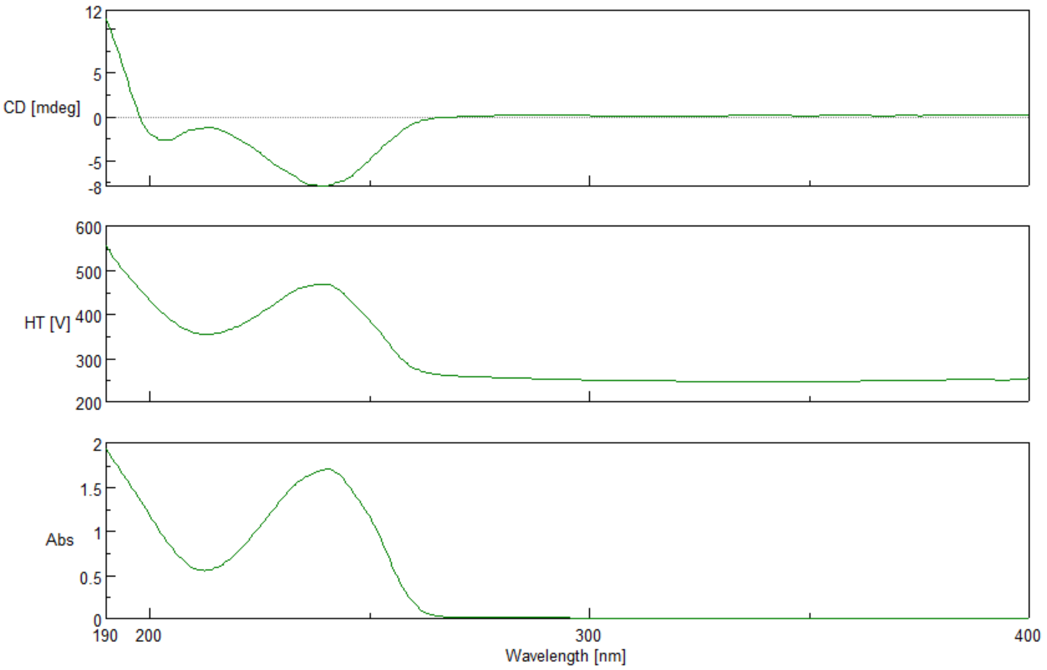

Figure S26. UV and CD spectrum of **4**

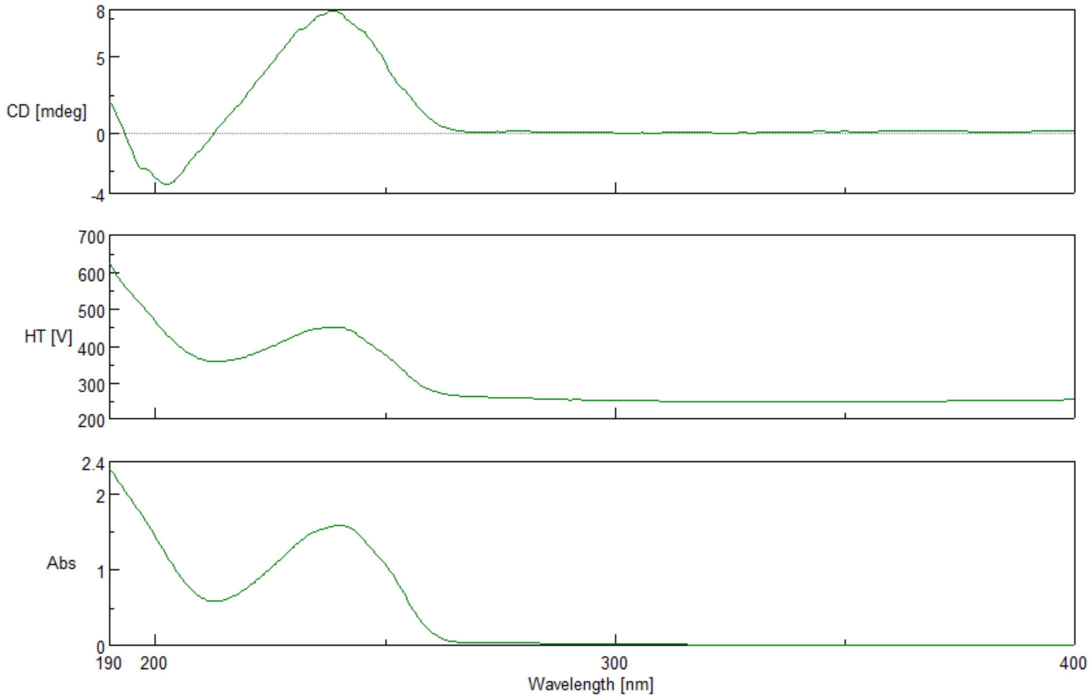

Figure S27. UV and CD spectrum of **5**

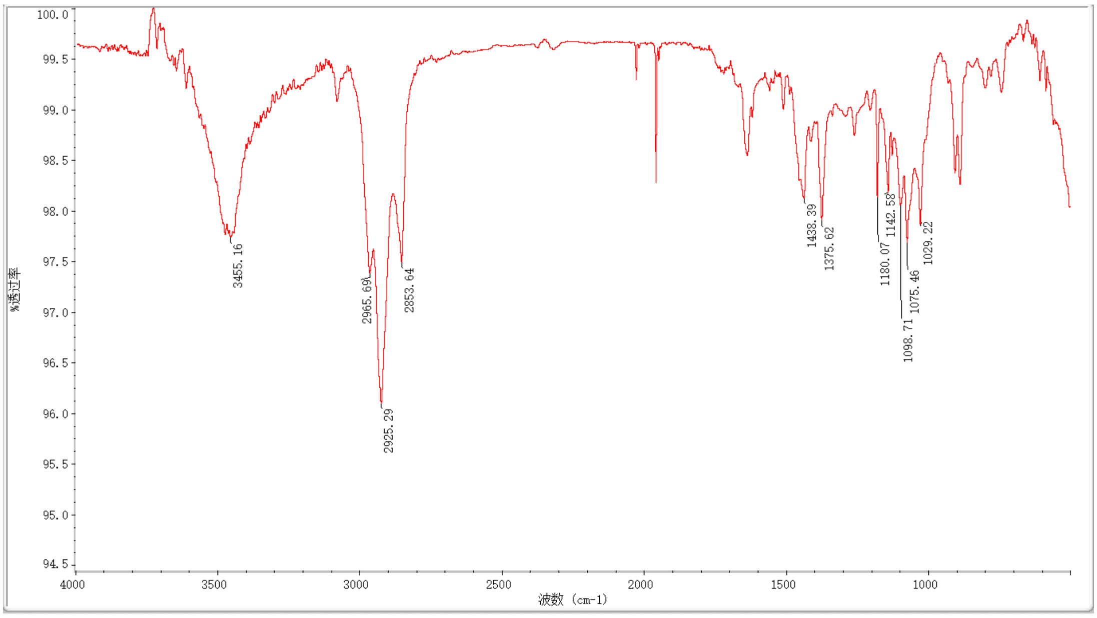

Figure S28. IR spectrum for mixture of **4** and **5**

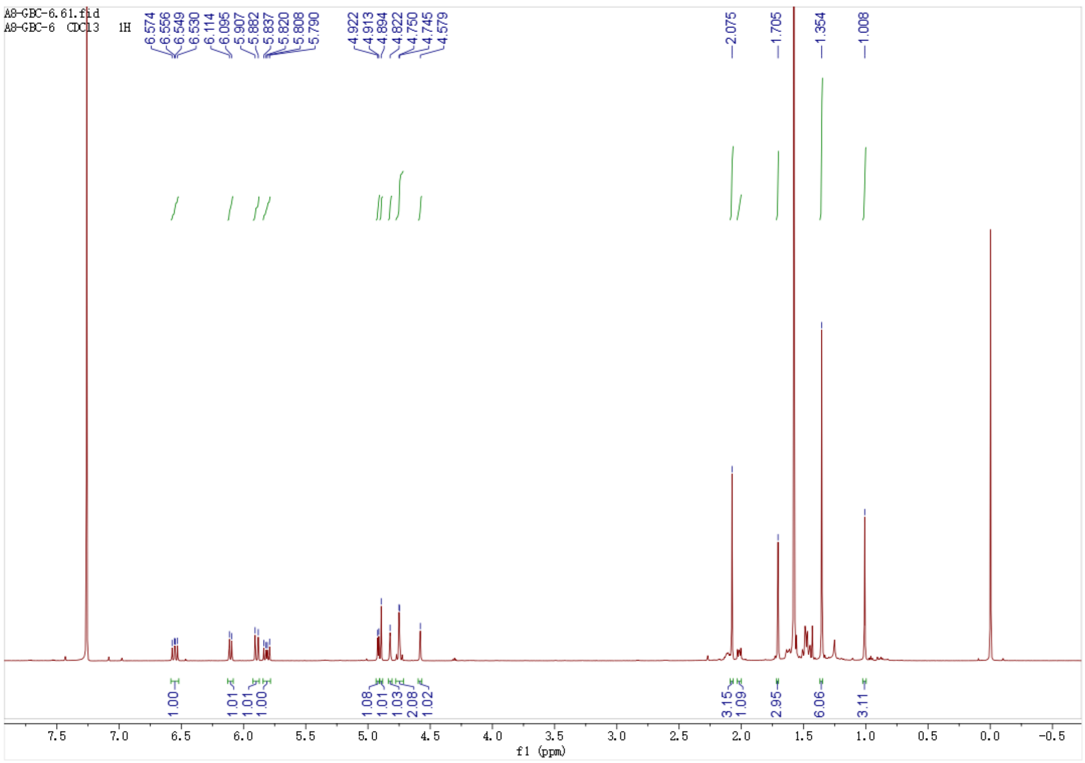

Figure S29. ^{1}H NMR spectrum of **6** (600MHz, $CDCl_3$)

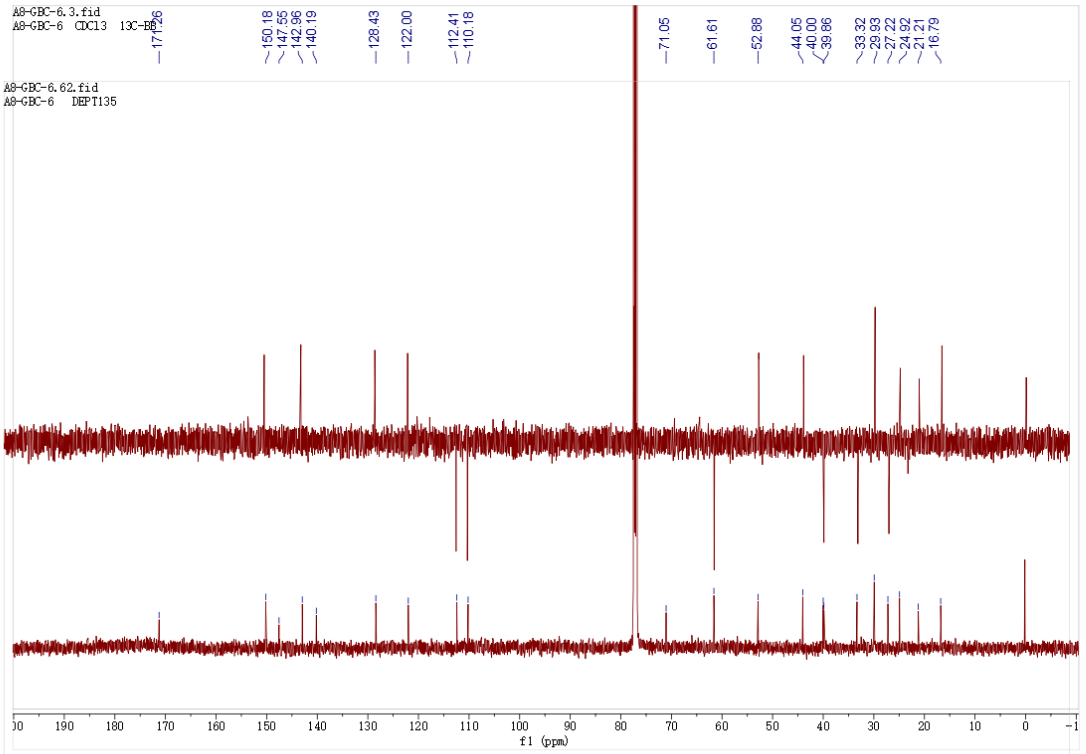

Figure S30. ^{13}C NMR spectrum of **6** (125 MHz, $CDCl_3$)

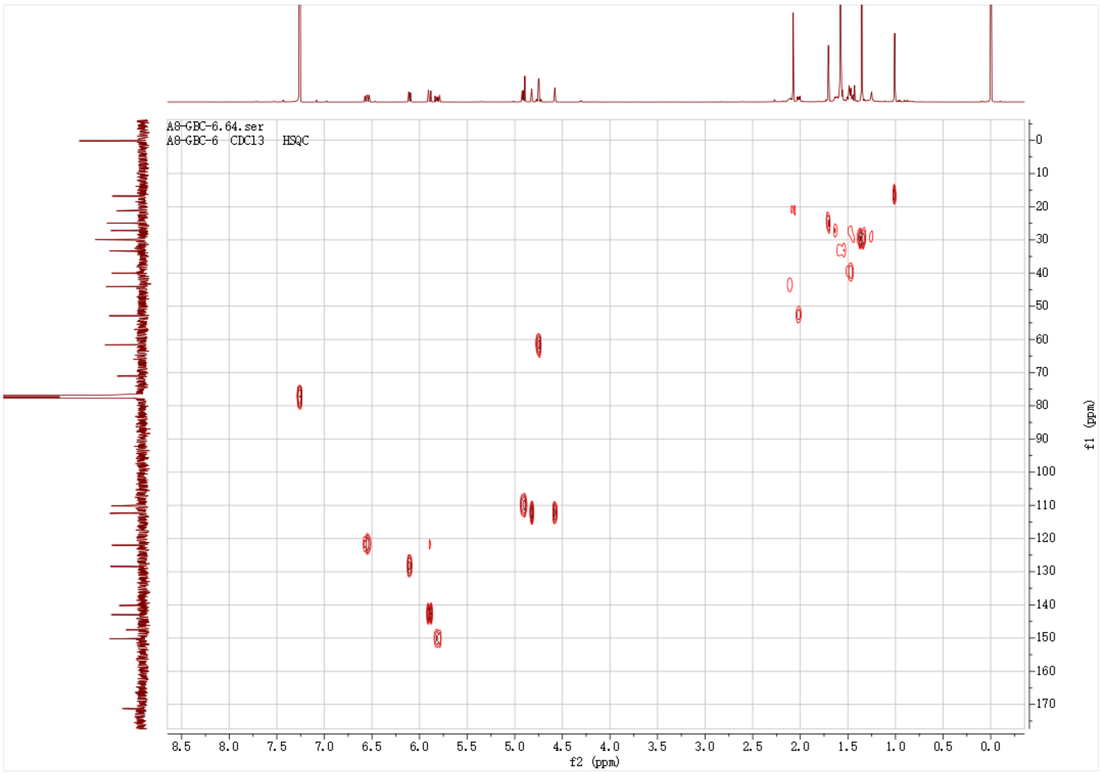

Figure S31. HSQC spectrum of **6** (600 MHz, $CDCl_3$)

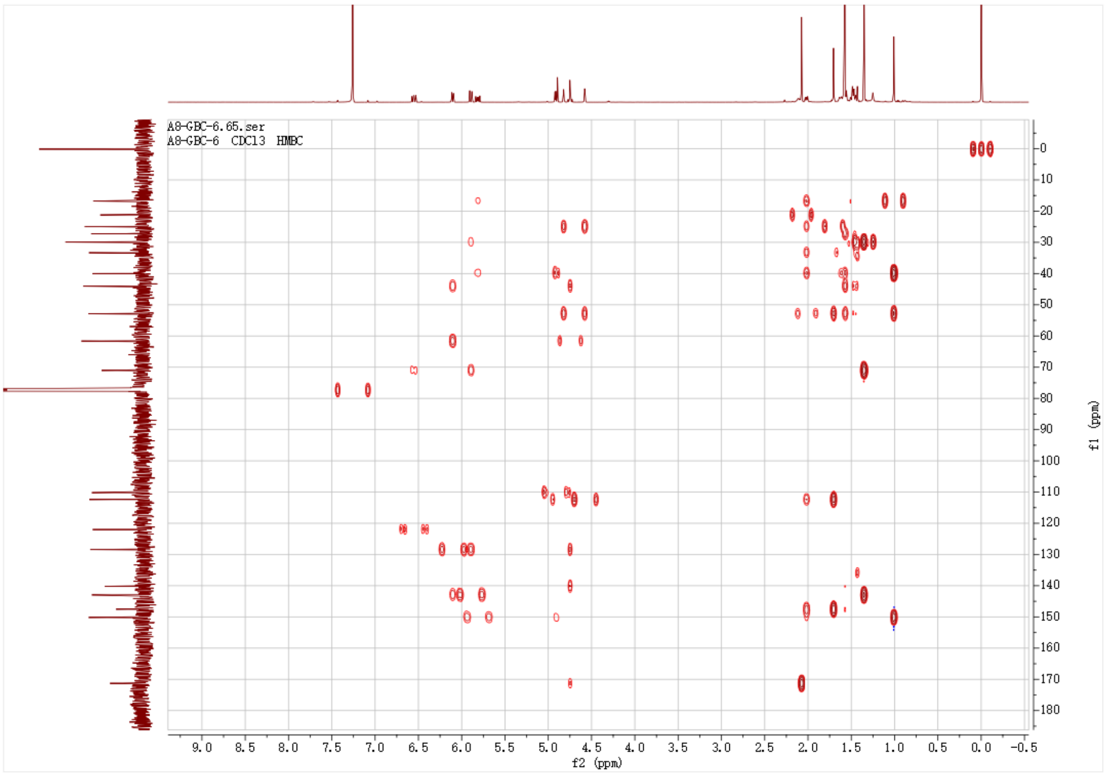

Figure S32. HMBC spectrum of **6** (600 MHz, $CDCl_3$)

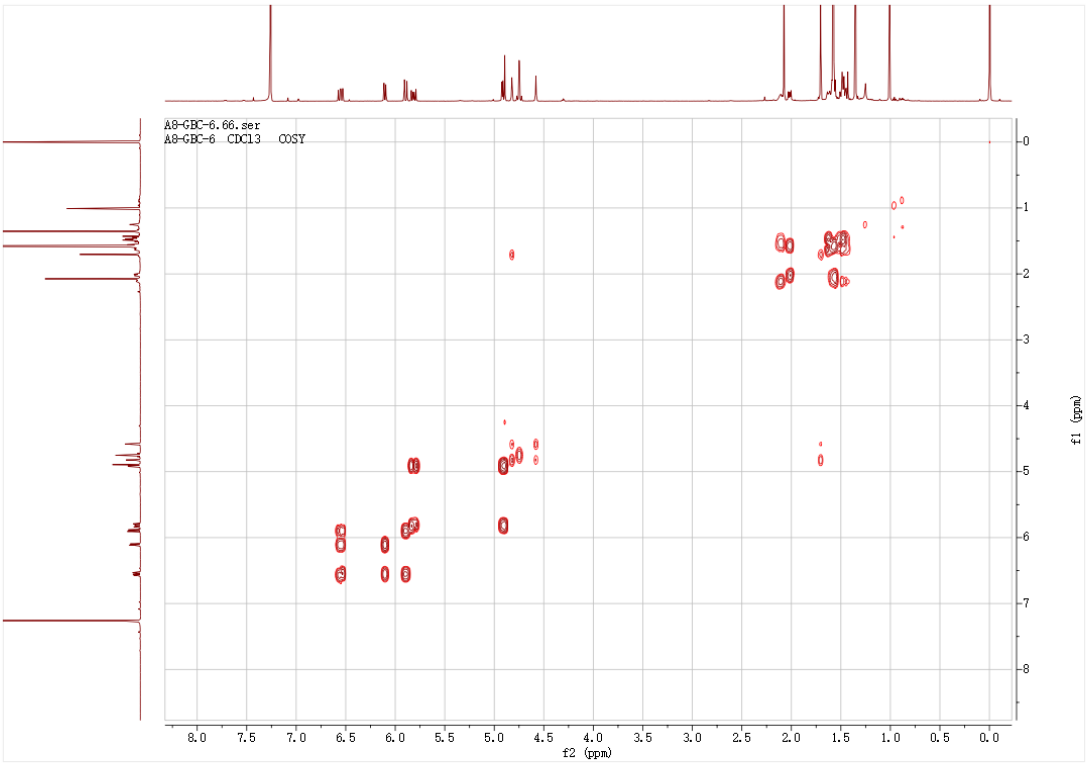

Figure S33. ^{1}H–^{1}H COSY spectrum of **6** (600 MHz, CDCl3)

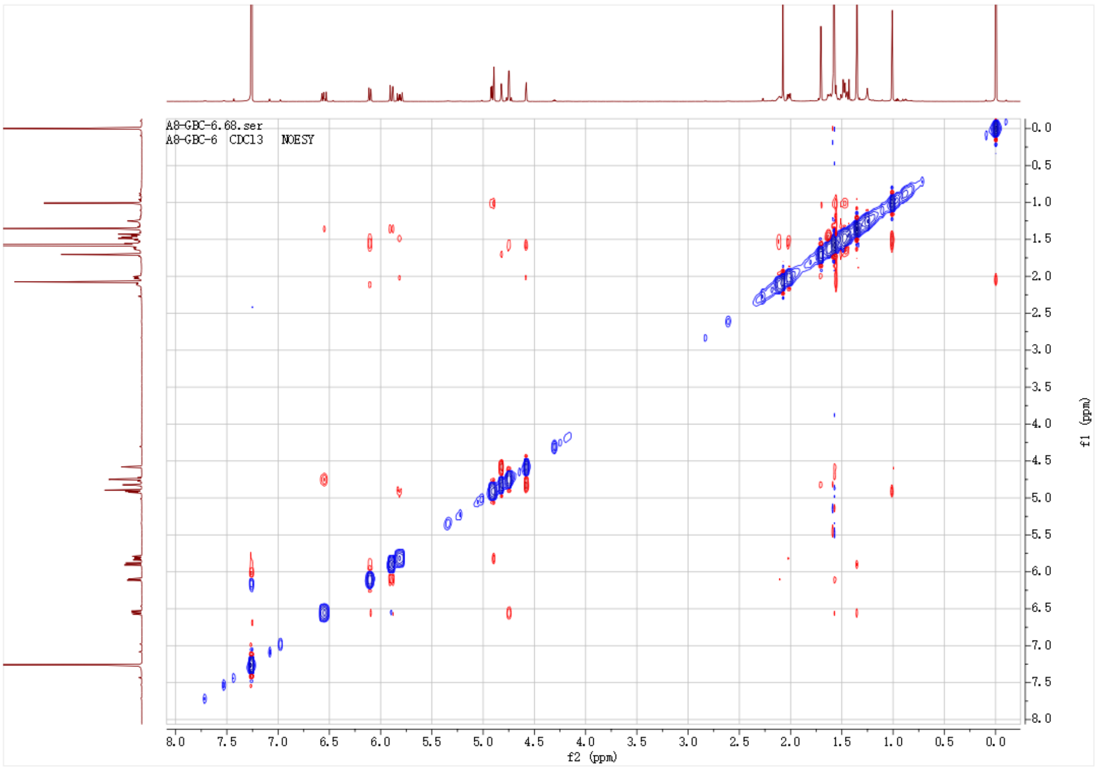

Figure S34. NOESY spectrum of **6** (600 MHz, CDCl3)

Qualitative Analysis Report

**Data Filename**	ESIH202103307.d	**Sample Name**	A8-GBC-6
**Sample ID**		**Position**	P1-C1
**Instrument Name**	Agilent G6520 Q-TOF	**Acq Method**	20160322_MS_ESIH_POS_1min.m
**Acquired Time**	7/2/2021 18:15:16	**IRM Calibration Status**	Success
**DA Method**	small molecular data analysis method.m	**Comment**	ESIH by ZZY

User Spectra

Fragmentor Voltage	Collision Energy	Ionization Mode
135	0	ESI

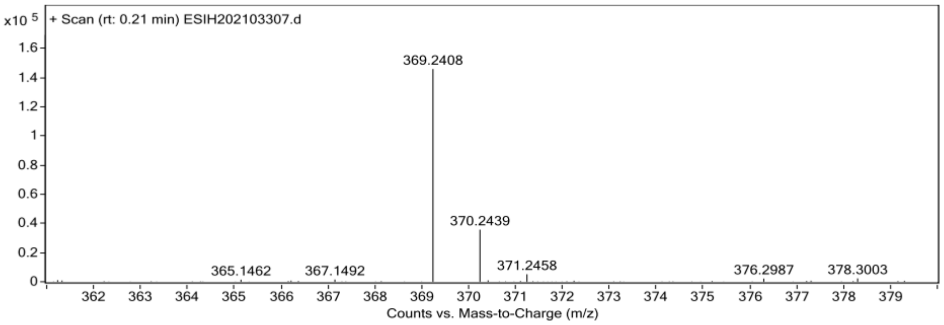

Formula Calculator Results

m/z	Calc m/z	Diff (mDa)	Diff (ppm)	Ion Formula	Ion
369.2408	369.24	-0.77	-2.09	C22 H34 Na O3	(M+Na)+

--- End Of Report ---

Figure S35. HRESIMS spectrum of **6**

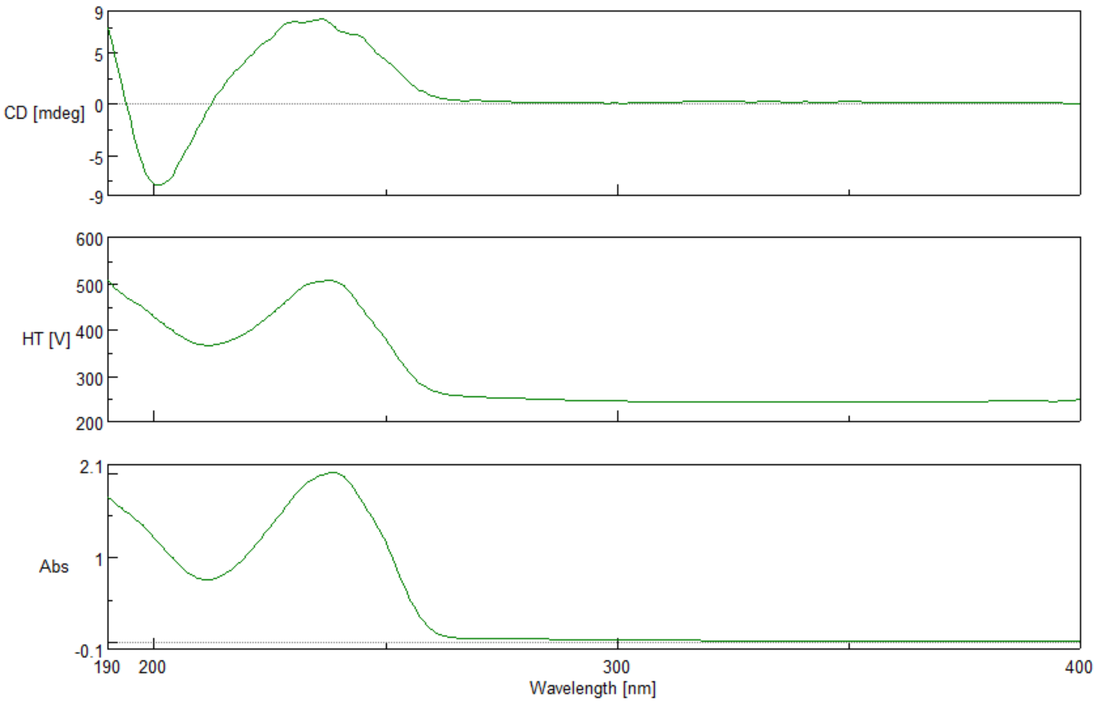

Figure S36. UV and CD spectrum of **6**

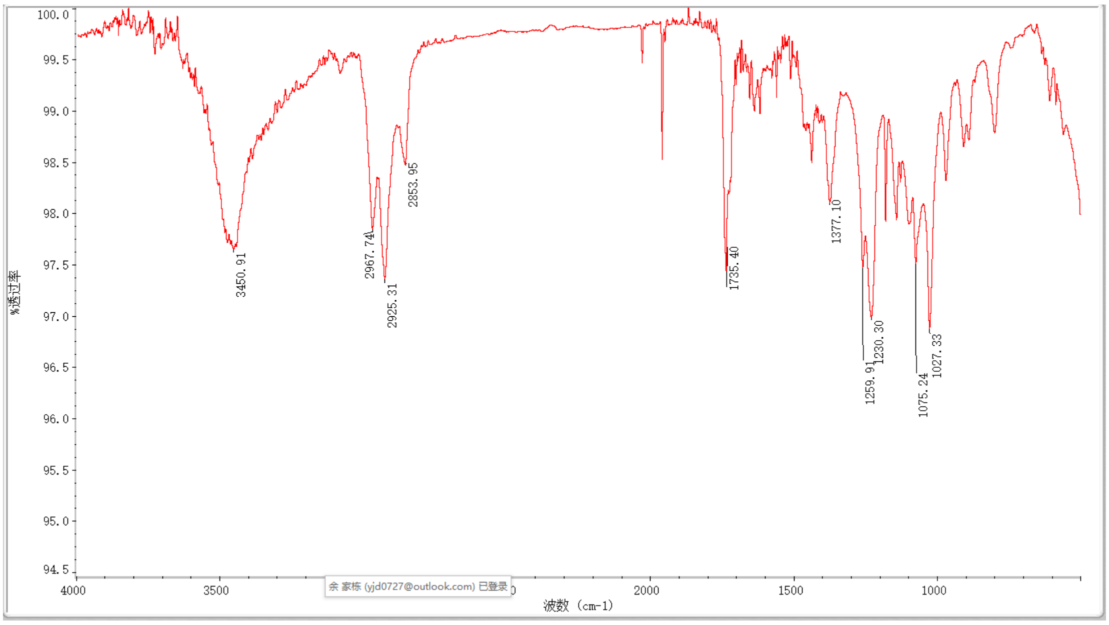

Figure S37. IR spectrum of **6**

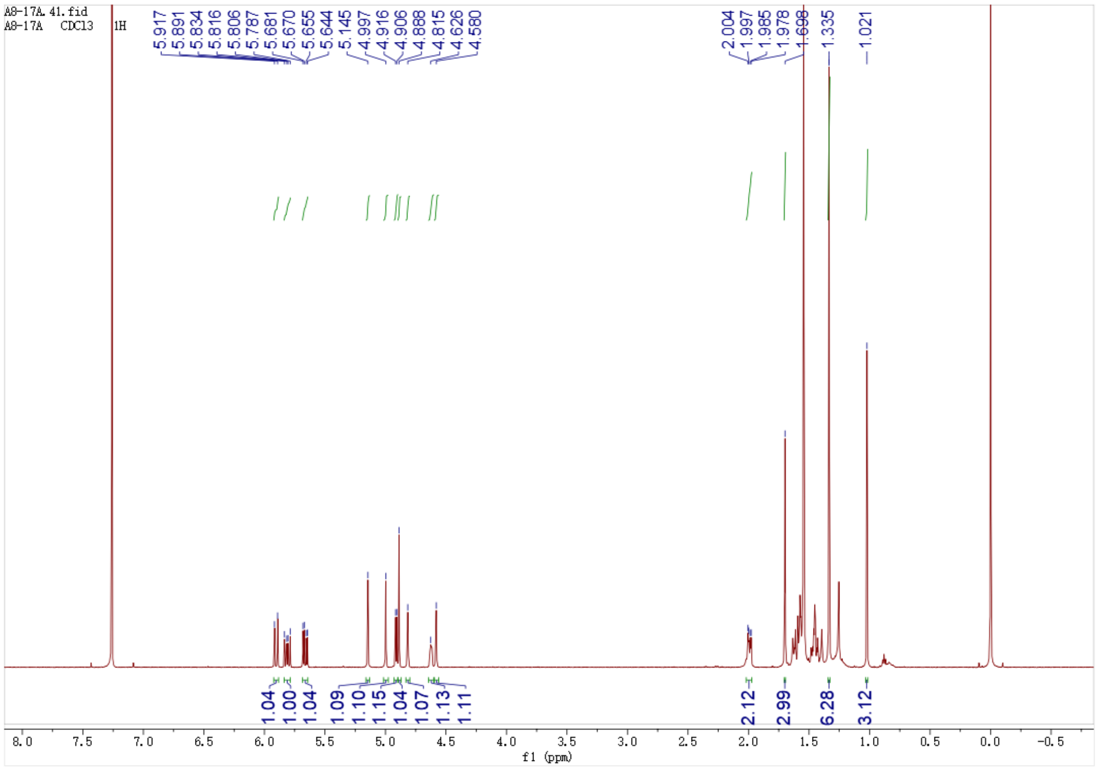

Figure S38. ^{1}H NMR spectrum of **7** (600MHz, $CDCl_3$)

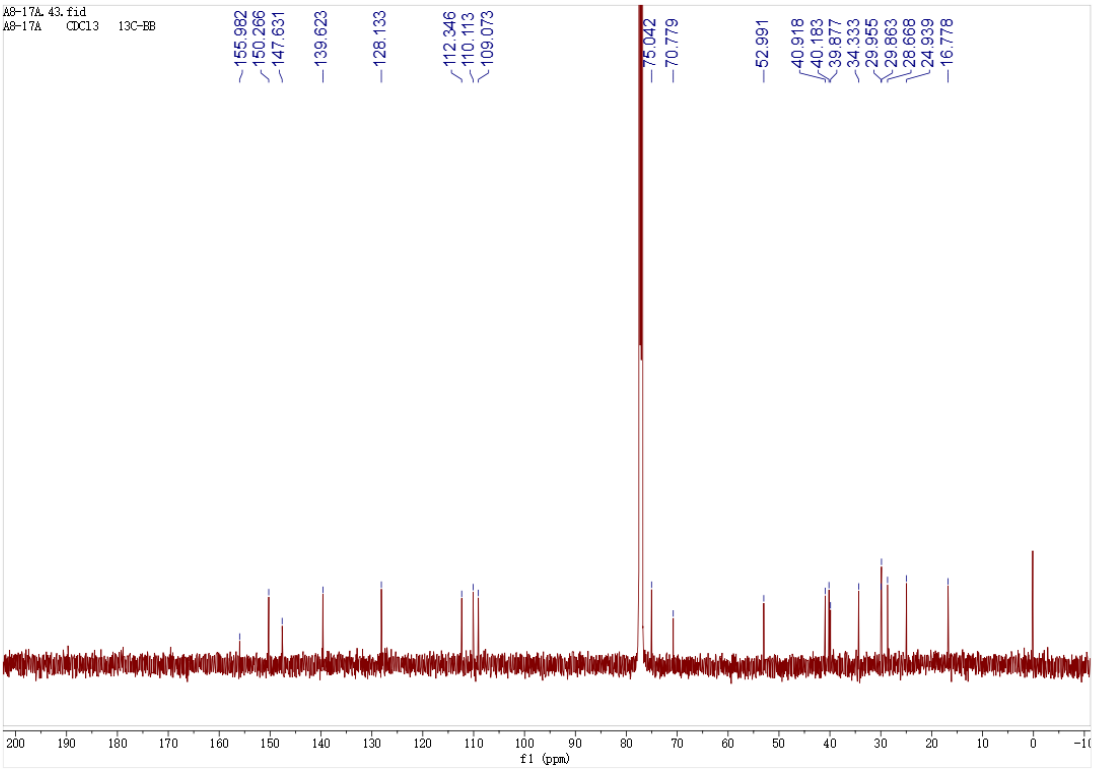

Figure S39. ^{13}C NMR spectrum of **7** (125 MHz, CDCl3)

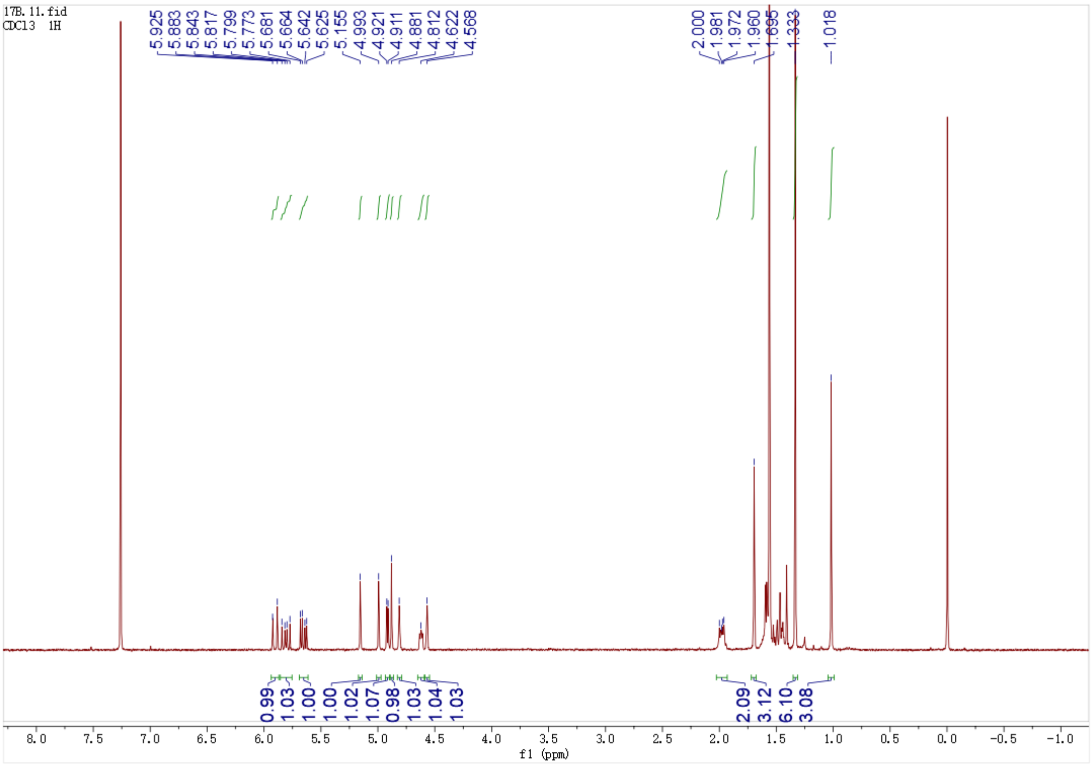

Figure S40. ^{1}H NMR spectrum of **8** (600MHz, CDCl3)

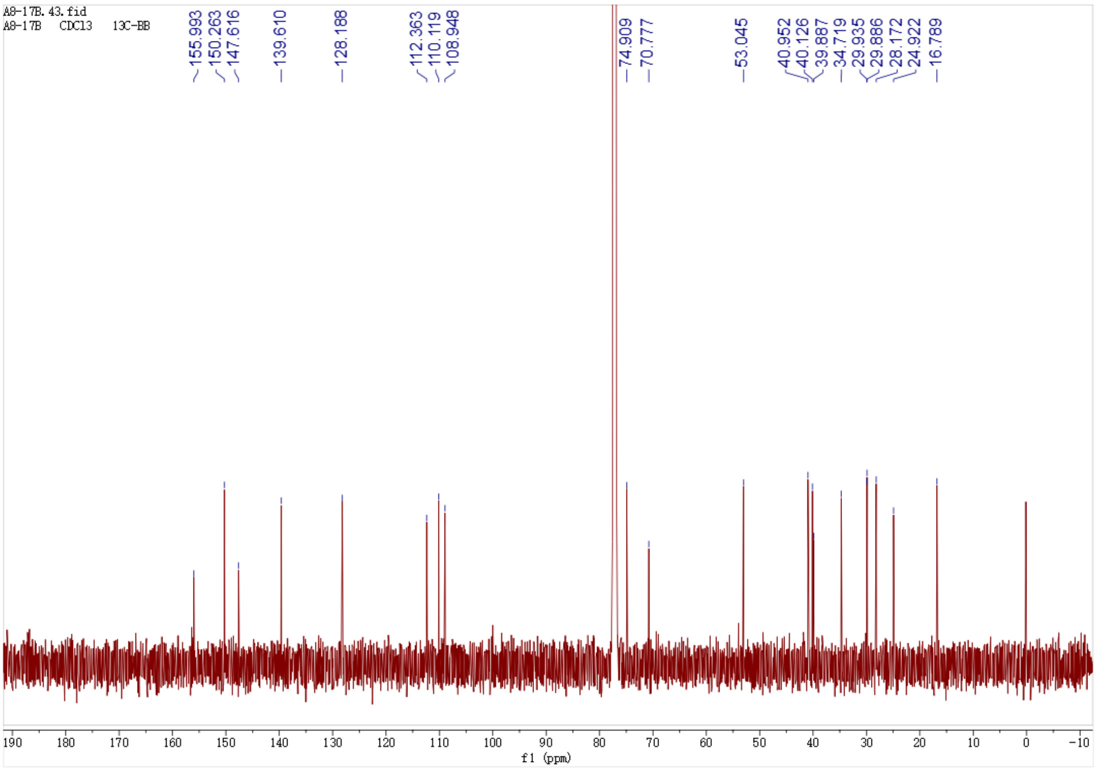

Figure S41. ^{13}C NMR spectrum of **8** (125 MHz, $CDCl_3$)

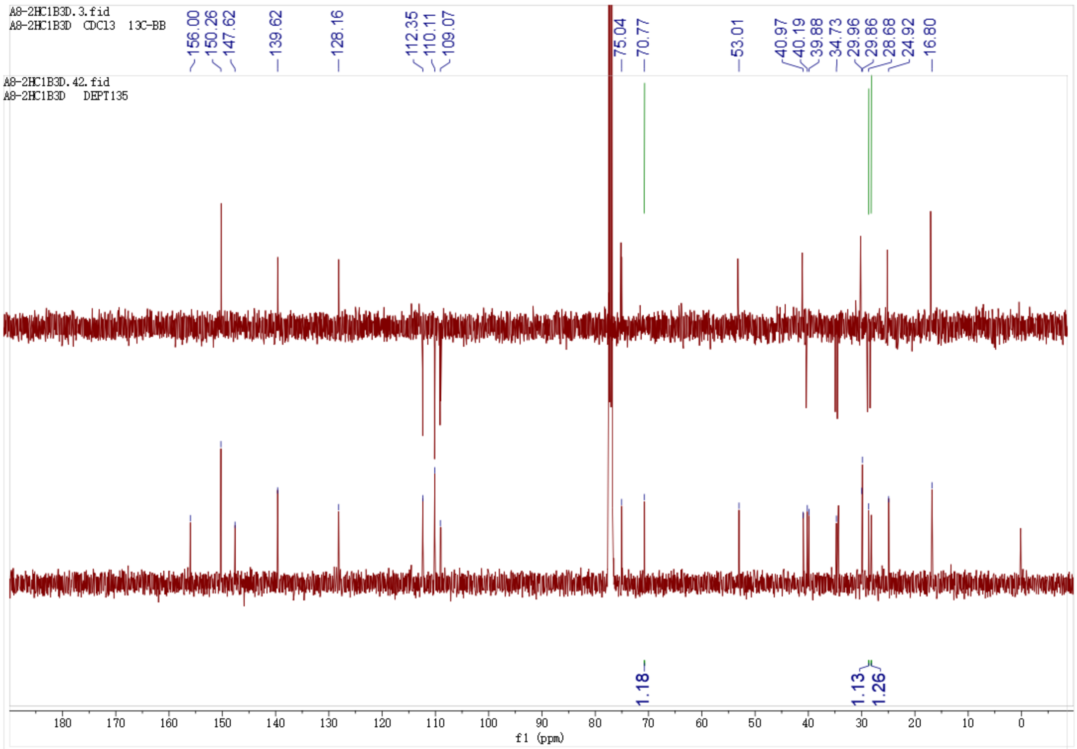

Figure S42. ^{13}C NMR spectrum for mixture of **7** and **8** (125 MHz, $CDCl_3$)

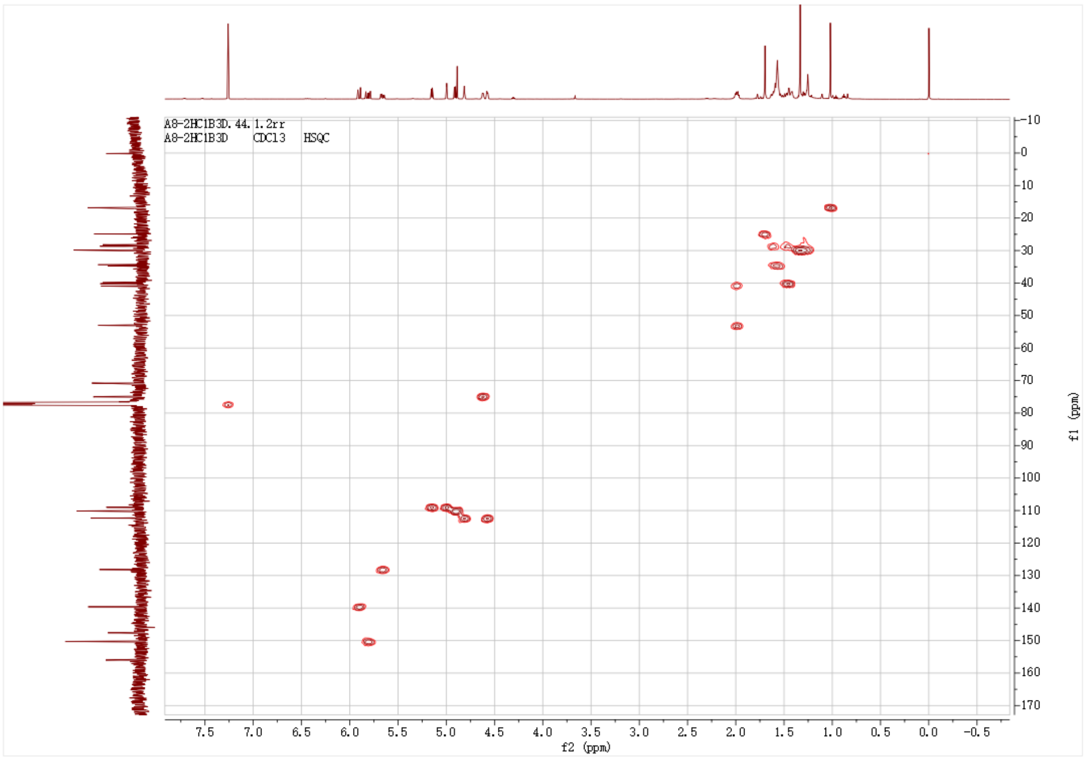

Figure S43. HSQC spectrum for mixture of **7** and **8** (600 MHz, $CDCl_3$)

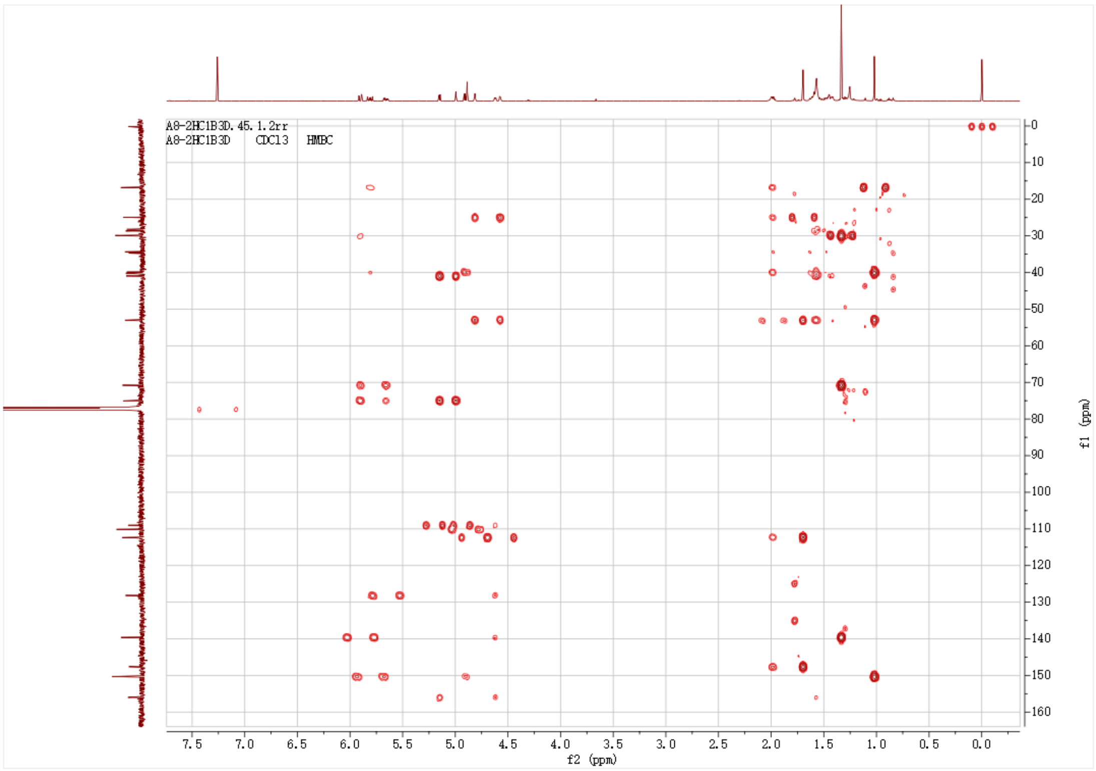

Figure S44. HMBC spectrum for mixture of **7** and **8** (600 MHz, $CDCl_3$)

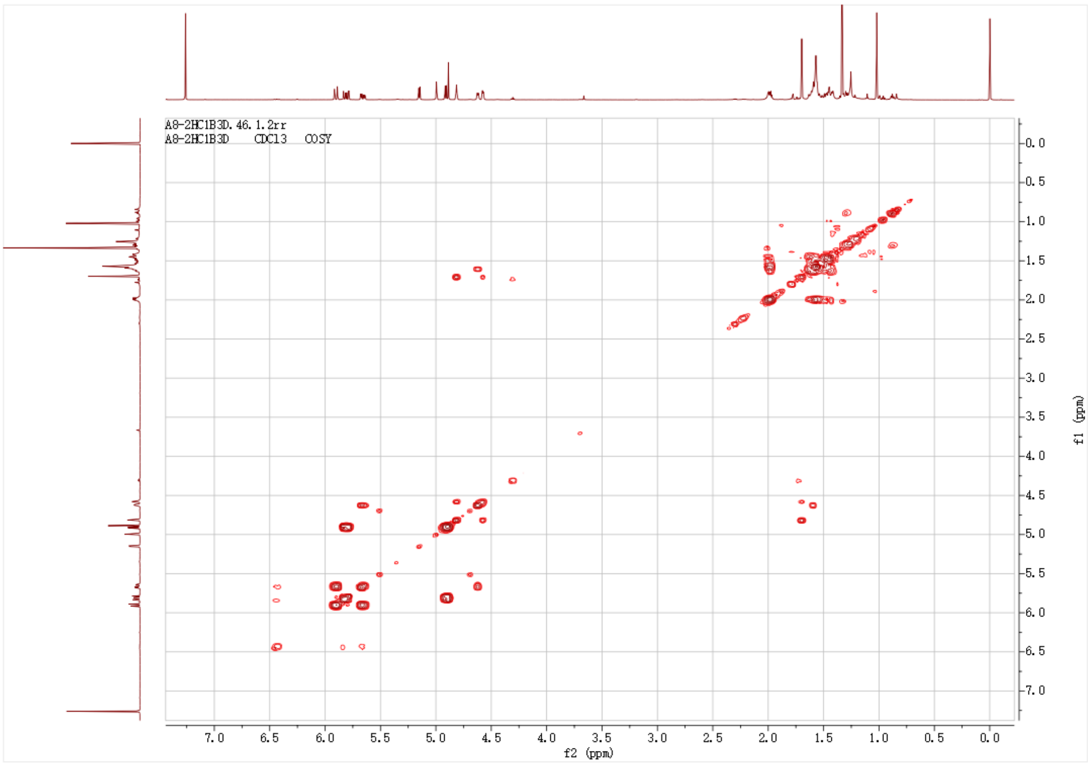

Figure S45. ¹H–¹H COSY spectrum for mixture of **7** and **8** (600 MHz, $CDCl_3$)

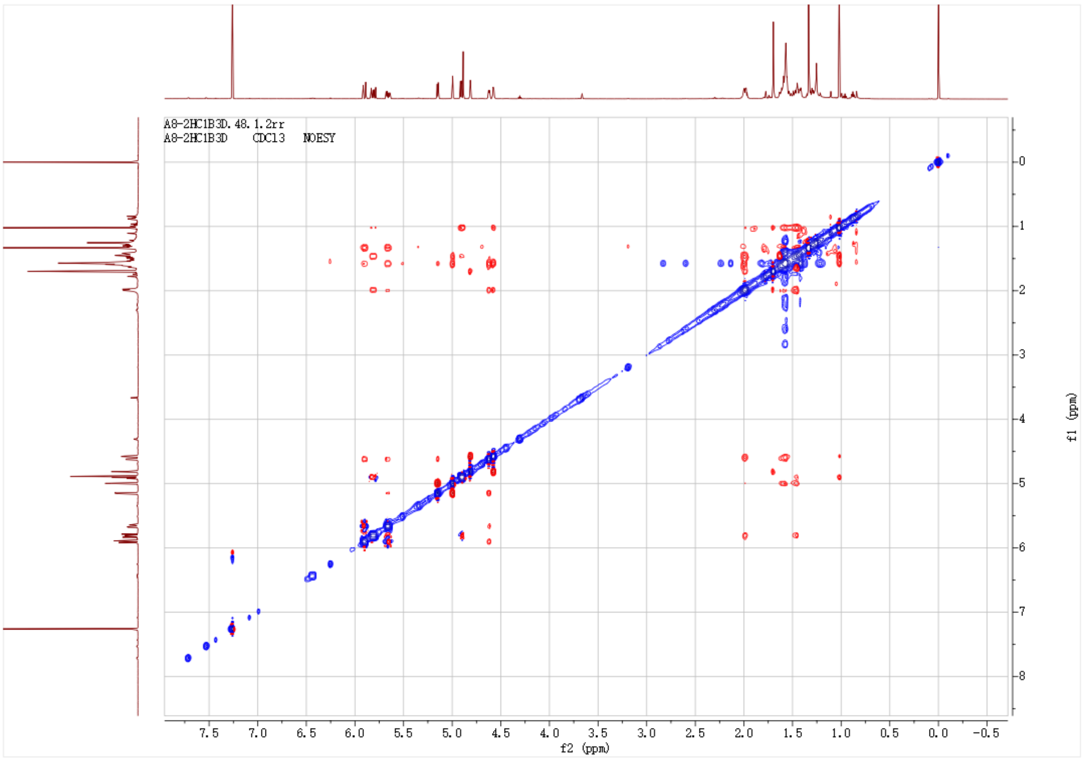

Figure S46. NOESY spectrum for mixture of **7** and **8** (600 MHz, $CDCl_3$)

Qualitative Analysis Report

**Data Filename**	ESIH202104715-1.d	**Sample Name**	A8-A8-2HC1B3D
**Sample ID**		**Position**	P1-B6
**Instrument Name**	Agilent G6520 Q-TOF	**Acq Method**	20160324_MS_ESIH_NEG_1min.m
**Acquired Time**	10/22/2021 19:29:36	**IRM Calibration Status**	Success
**DA Method**	small molecular data analysis method.m	**Comment**	ESIH by zhuzhenyun

User Spectra

Fragmentor Voltage	Collision Energy	Ionization Mode
175	0	ESI

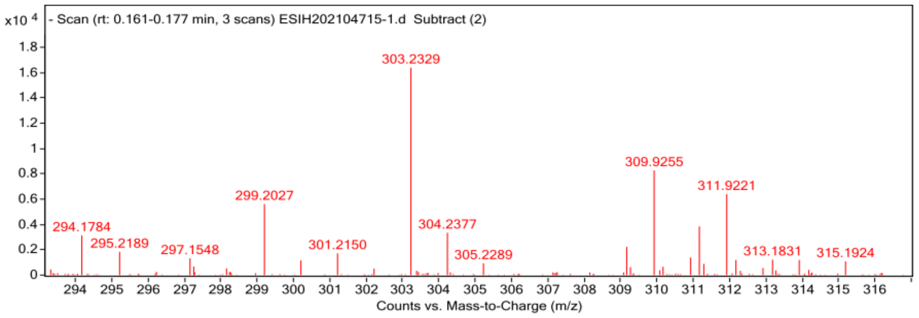

Formula Calculator Results

m/z	Calc m/z	Diff (mDa)	Diff (ppm)	Ion Formula	Ion
303.2329	303.233	0.01	0.02	C20 H31 O2	(M-H)-

--- End Of Report ---

Figure S47. HRESIMS spectrum for mixture of **7** and **8**

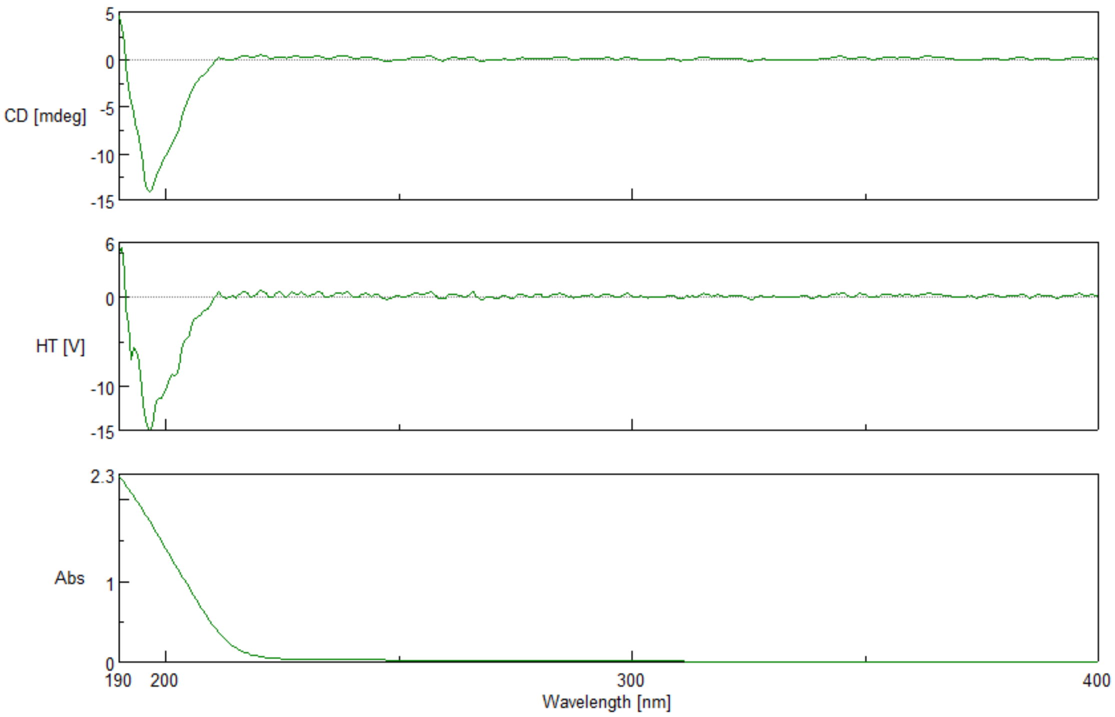

Figure S48. UV and CD spectrum of **7**

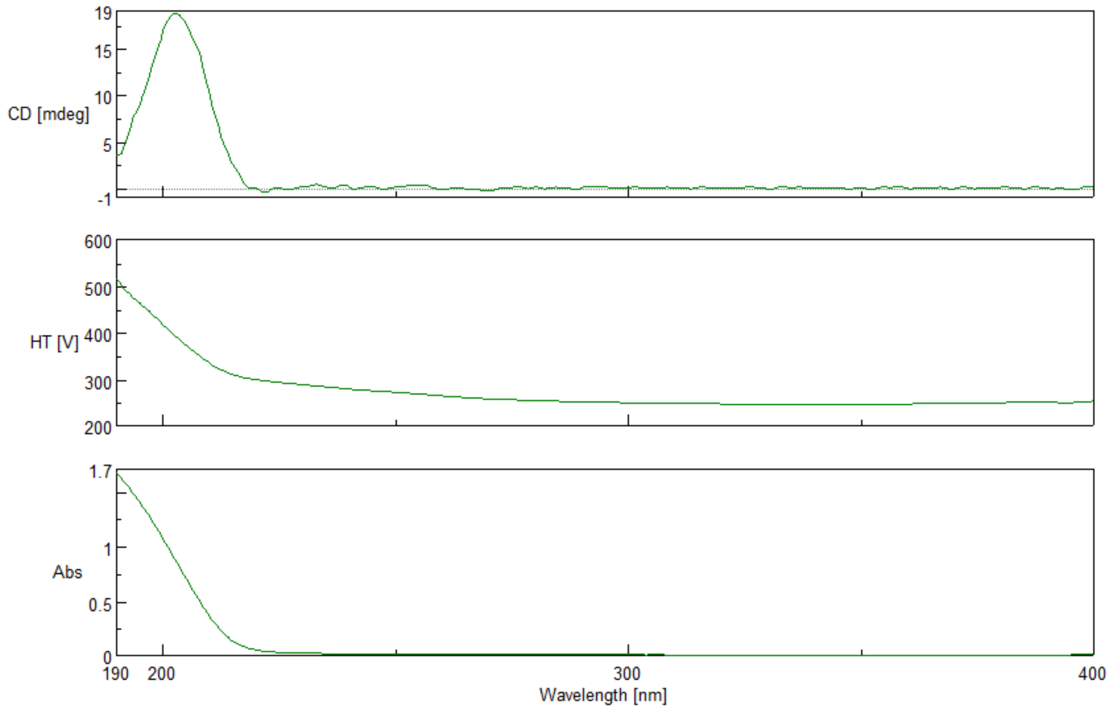

Figure S49. UV and CD spectrum of **8**

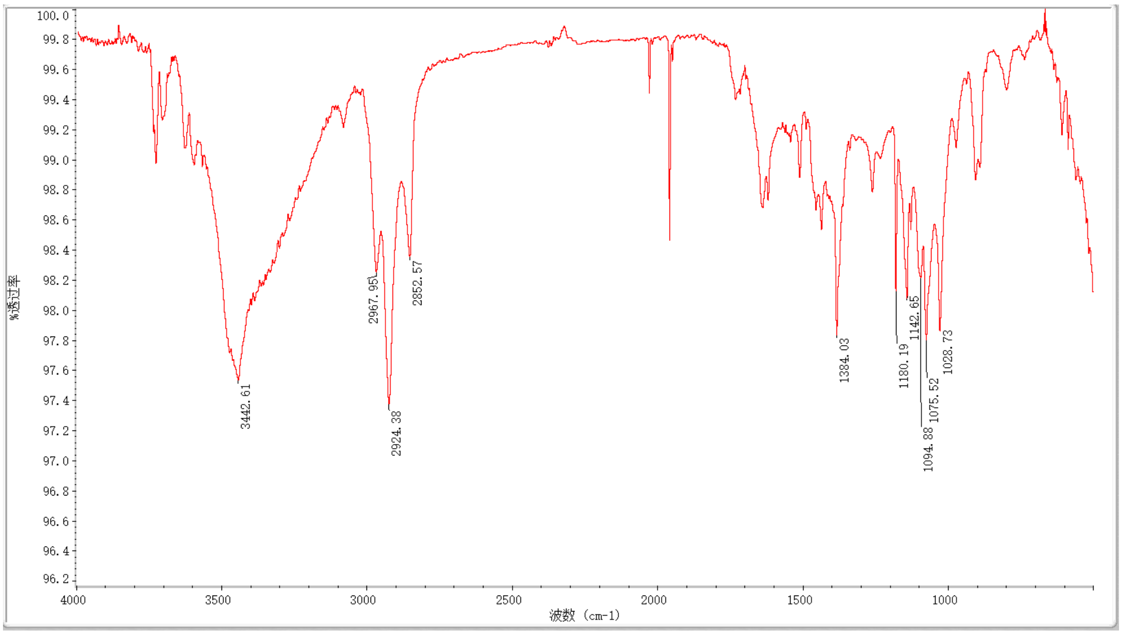

Figure S50. IR spectrum for mixture of **7** and **8**

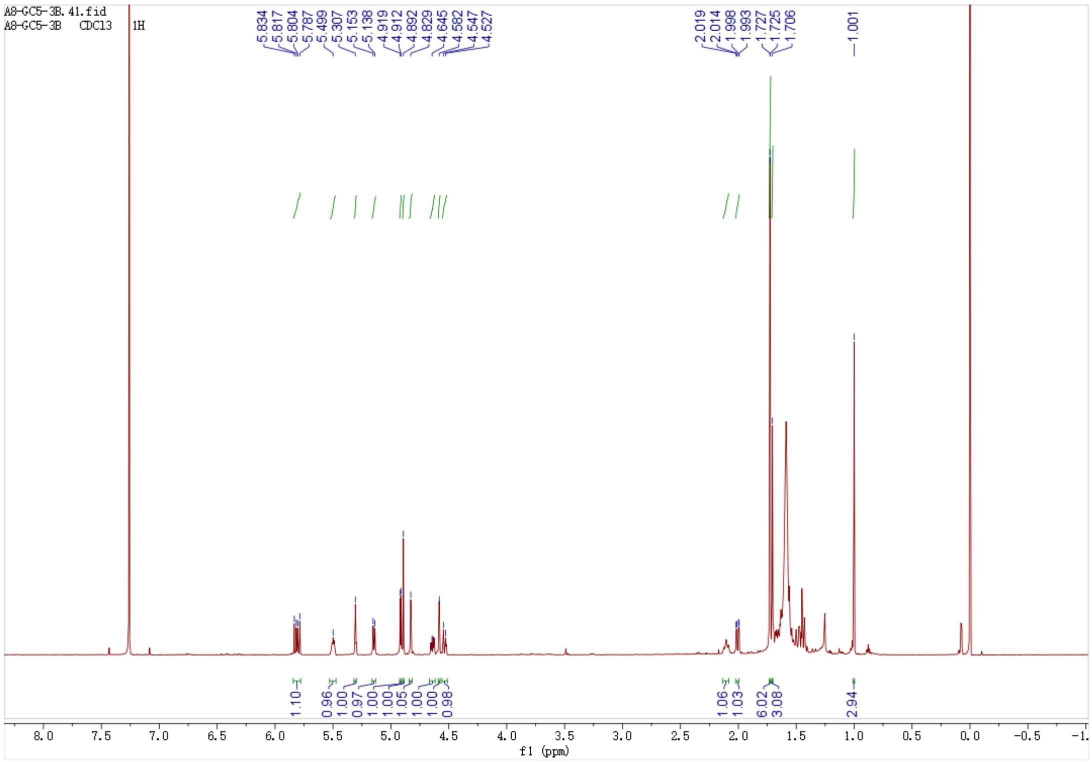

Figure S51. ^{1}H NMR spectrum of **9** (600MHz, CDCl3)

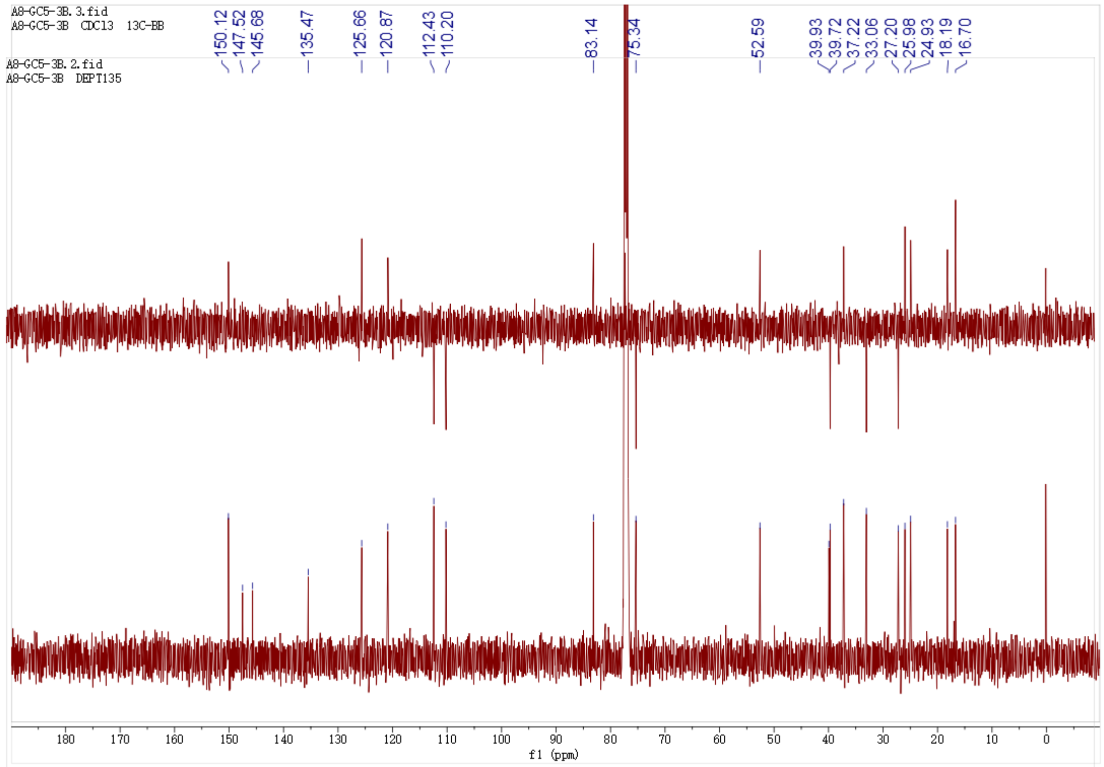

Figure S52. ^{13}C NMR spectrum of **9** (125 MHz, CDCl3)

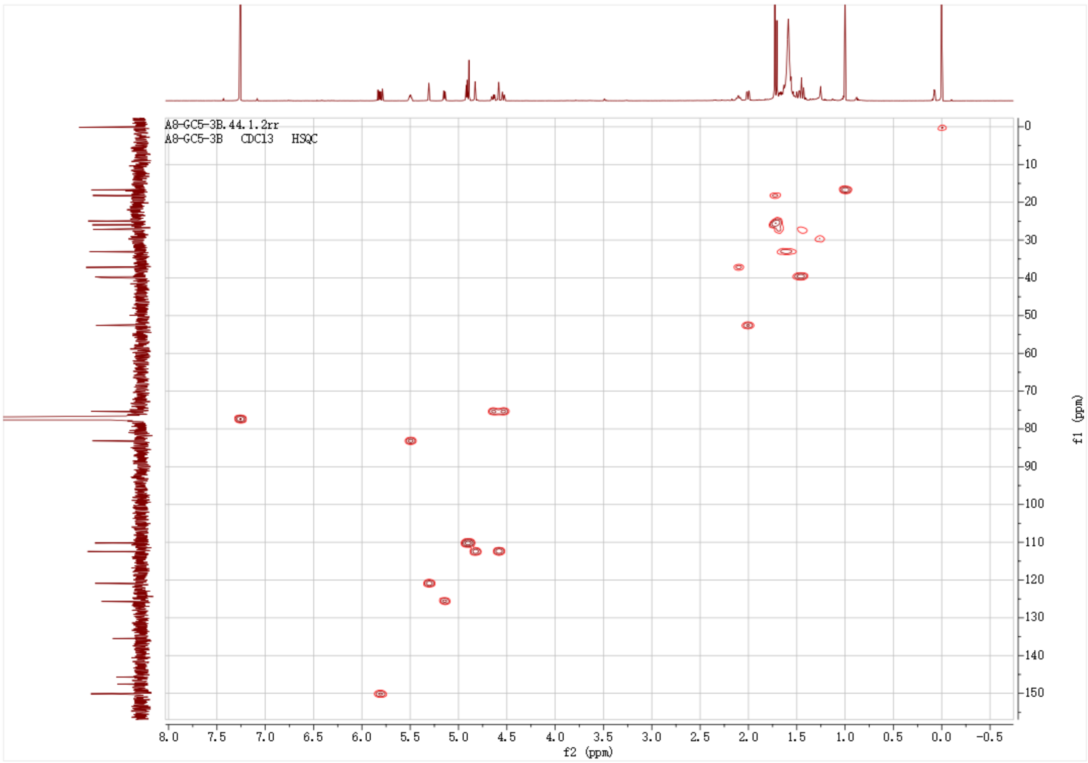

Figure S53. HSQC spectrum of **9** (600 MHz, CDCl3)

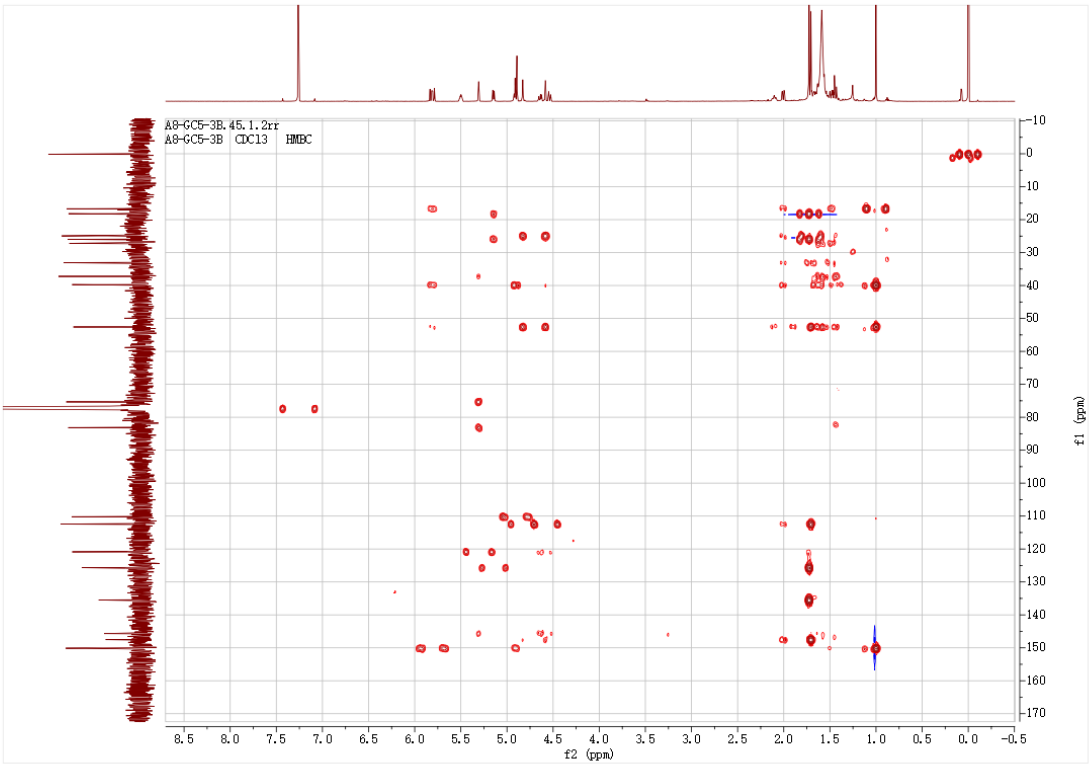

Figure S54. HMBC spectrum of **9** (600 MHz, CDCl3)

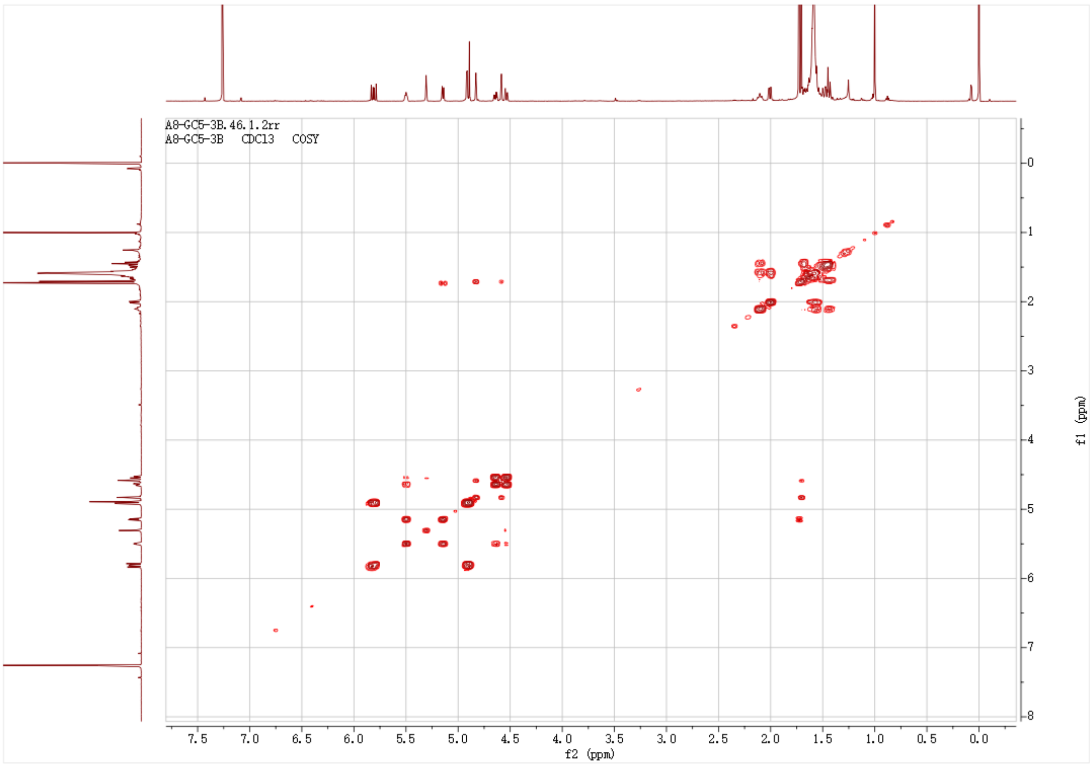

Figure S55. ^{1}H–^{1}H COSY spectrum of **9** (600 MHz, $CDCl_3$)

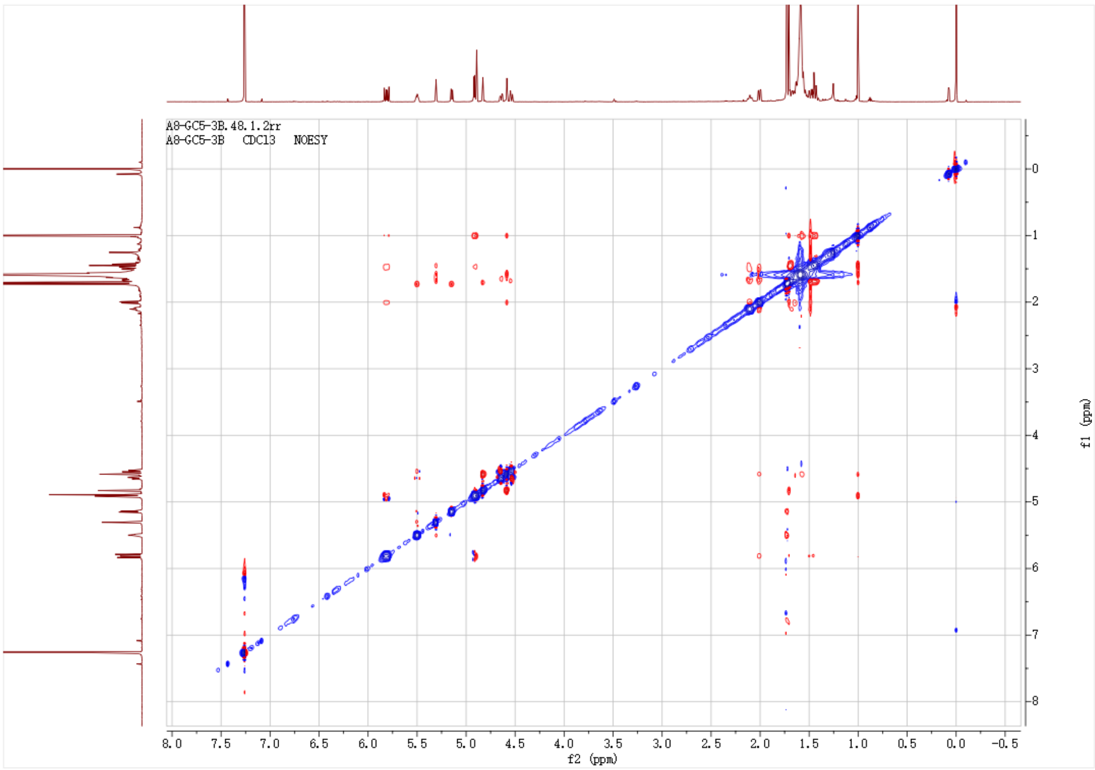

Figure S56. NOESY spectrum of **9** (600 MHz, $CDCl_3$)

D:\data\2021\EI202101751_A8-GC5-3B -c1 10/11/2021 3:20:55 PM

EI202101751_A8-GC5-3B -c1#5 RT: 0.83
T: + c EI Full ms [49.50-800.50]
m/z= 48-803

m/z	Intensity	Relative	Theo. Mass	Delta (mmu)	RDB equiv.	Composition
115.0559	1243035.0	1.70	115.0542	1.72	6.5	C_9H_7
117.0700	1692654.0	2.31	117.0699	0.16	5.5	C_9H_9
119.0858	4363781.0	5.95	119.0855	0.29	4.5	C_9H_{11}
120.0926	1004185.0	1.37	120.0934	-0.77	4.0	C_9H_{12}
121.0666	3271862.0	4.46	121.0648	1.85	4.5	$C_8H_9O_1$
121.1029	4869674.0	6.64	121.1012	1.72	3.5	C_9H_{13}
129.0727	1209233.0	1.65	129.0699	2.81	6.5	$C_{10}H_9$
131.0846	3008170.0	4.10	131.0855	-0.90	5.5	$C_{10}H_{11}$
163.1088	1405592.0	1.92	163.1117	-2.93	4.5	$C_{11}H_{15}O_1$
185.1328	961094.0	1.31	185.1325	0.28	6.5	$C_{14}H_{17}$
187.1125	1451815.0	1.98	187.1117	0.71	6.5	$C_{13}H_{15}O_1$
187.1487	2975158.0	4.06	187.1481	0.54	5.5	$C_{14}H_{19}$
189.1275	2455728.0	3.35	189.1274	0.07	5.5	$C_{13}H_{17}O_1$
190.1341	1046104.0	1.43	190.1352	-1.12	5.0	$C_{13}H_{18}O_1$
201.1271	4531159.0	6.18	201.1274	-0.33	6.5	$C_{14}H_{17}O_1$
201.1639	1104557.0	1.51	201.1638	0.12	5.5	$C_{15}H_{21}$
202.1334	1760286.0	2.40	202.1352	-1.77	6.0	$C_{14}H_{18}O_1$
203.1428	3368857.0	4.60	203.1430	-0.24	5.5	$C_{14}H_{19}O_1$
205.1586	1191392.0	1.63	205.1587	-0.08	4.5	$C_{14}H_{21}O_1$
215.1430	3745532.0	5.11	215.1430	-0.05	6.5	$C_{15}H_{19}O_1$
216.1487	1480633.0	2.02	216.1509	-2.19	6.0	$C_{15}H_{20}O_1$
217.1590	12308992.0	16.79	217.1587	0.29	5.5	$C_{15}H_{21}O_1$
229.1588	1458979.0	1.99	229.1587	0.11	6.5	$C_{16}H_{21}O_1$
243.1744	3528118.0	4.81	243.1743	0.04	6.5	$C_{17}H_{23}O_1$
257.1911	1261285.0	1.72	257.1900	1.14	6.5	$C_{18}H_{25}O_1$
271.2056	73304064.0	100.00	271.2056	-0.05	6.5	$C_{19}H_{27}O_1$
284.2138	979916.0	1.34	284.2135	0.30	7.0	$C_{20}H_{28}O_1$
285.2203	1427519.0	1.95	285.2213	-1.04	6.5	$C_{20}H_{29}O_1$
286.2288	2825975.0	3.86	286.2291	-0.27	6.0	$C_{20}H_{30}O_1$

Figure S57. HREIMS spectrum of **9**

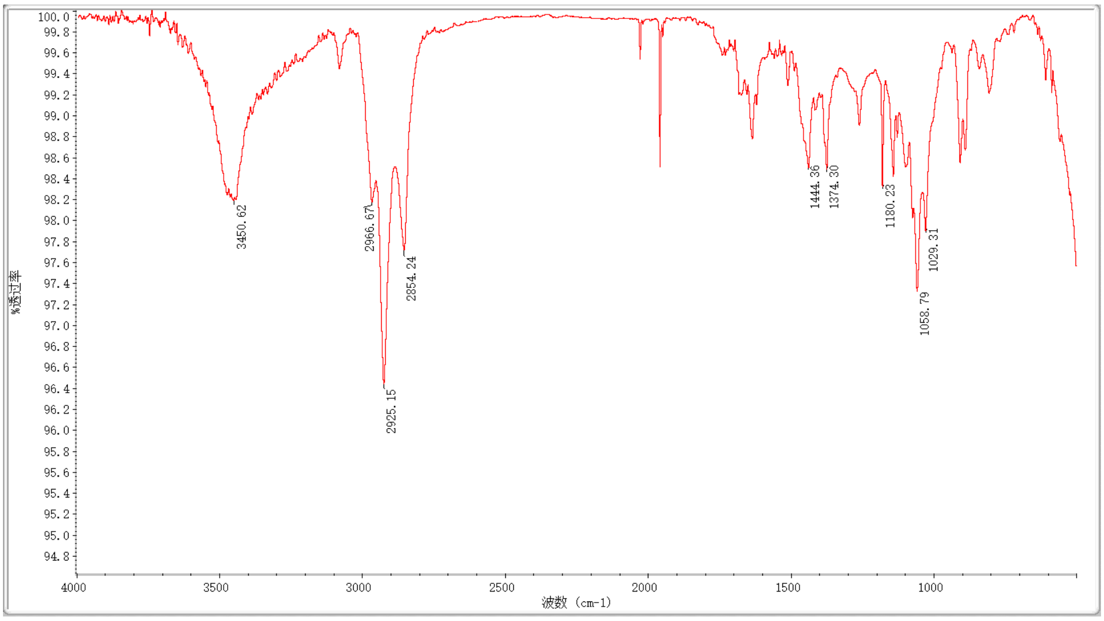

Figure S58. UV and CD spectrum of **9**

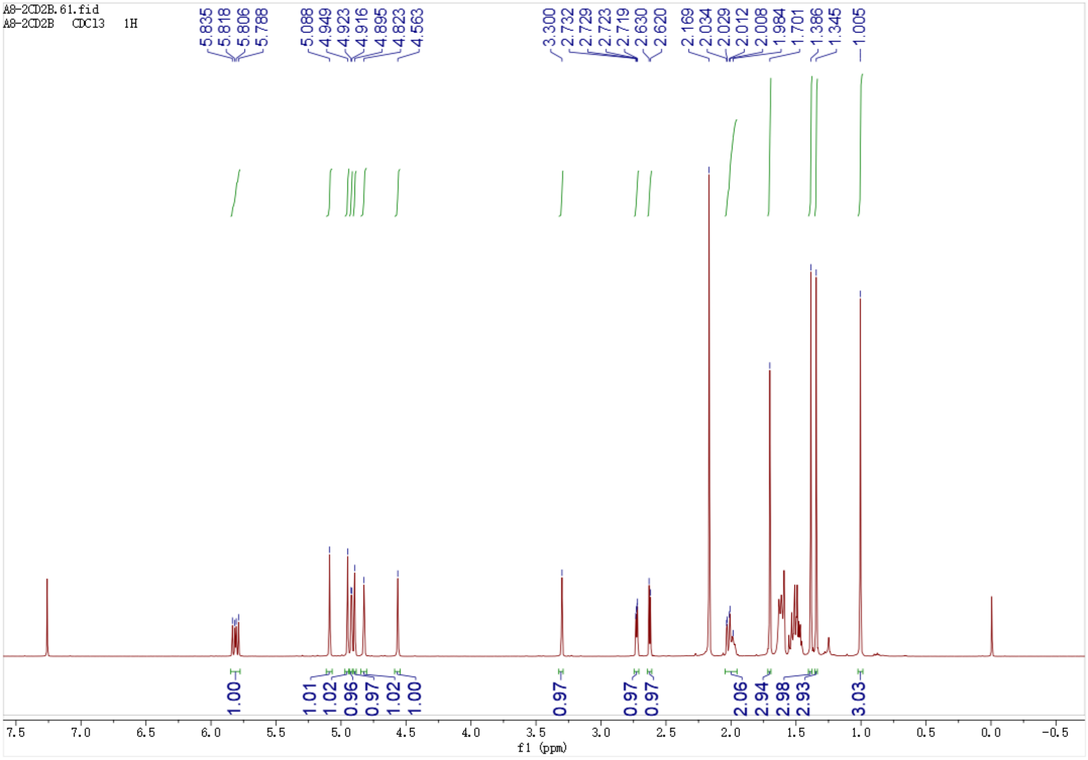

Figure S59. ¹H NMR spectrum of **10** (600MHz, $CDCl_3$)

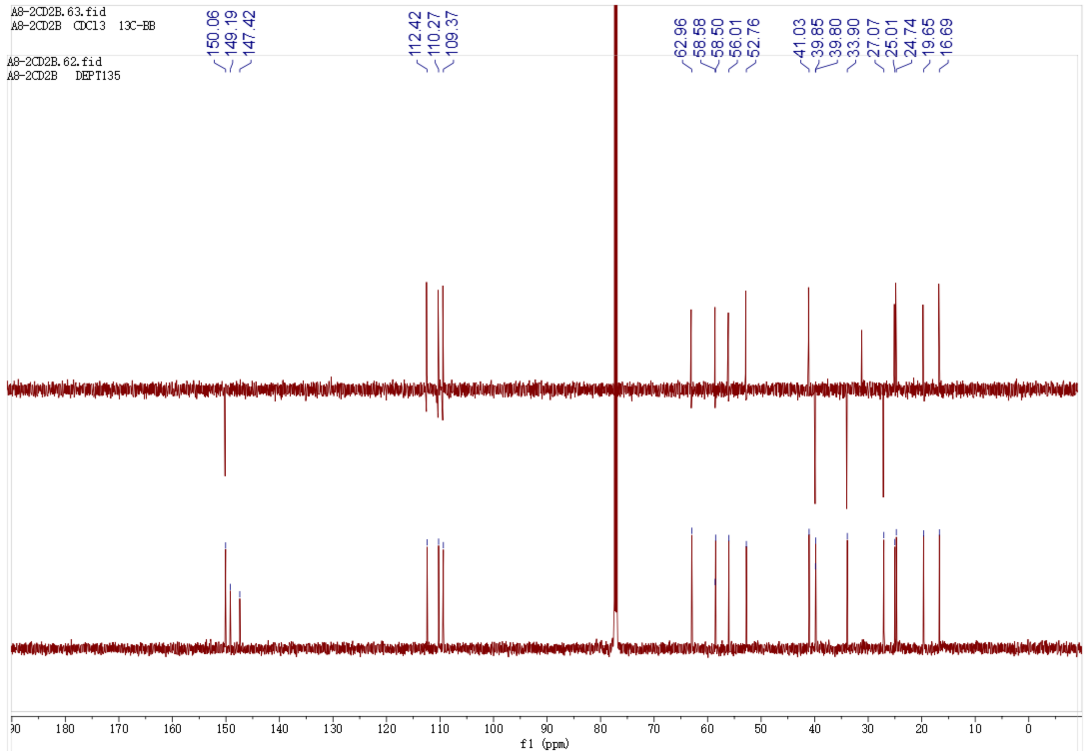

Figure S60. ¹³C NMR spectrum of **10** (125 MHz, $CDCl_3$)

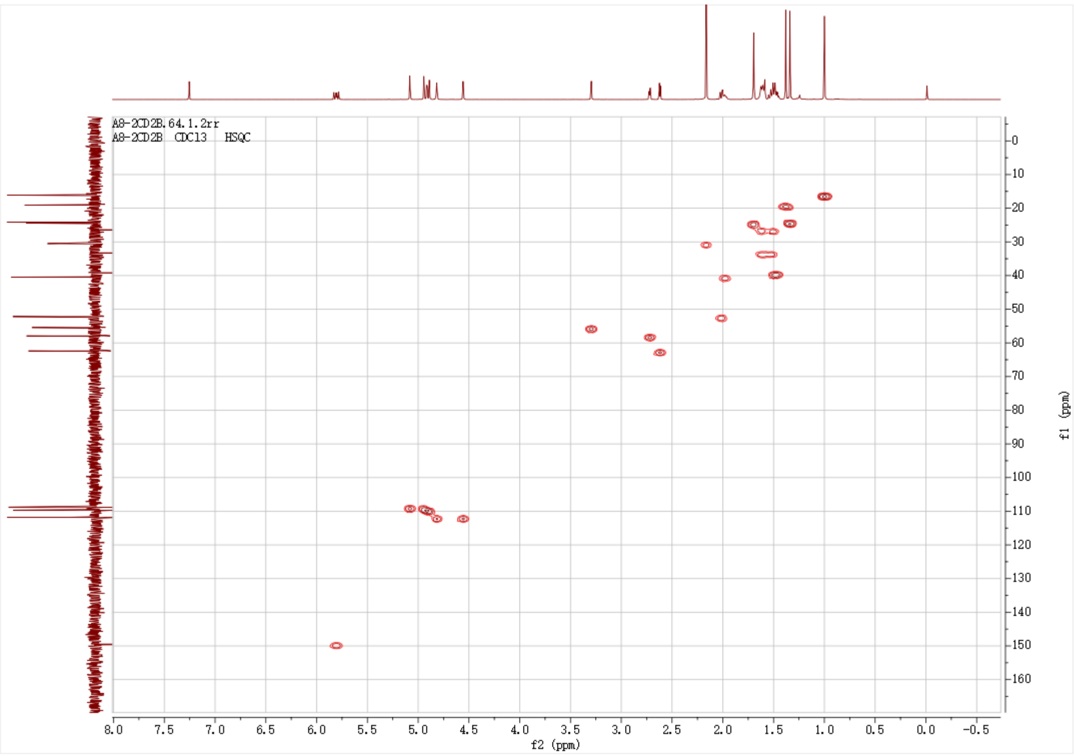

Figure S61. HSQC spectrum of **10** (600 MHz, $CDCl_3$)

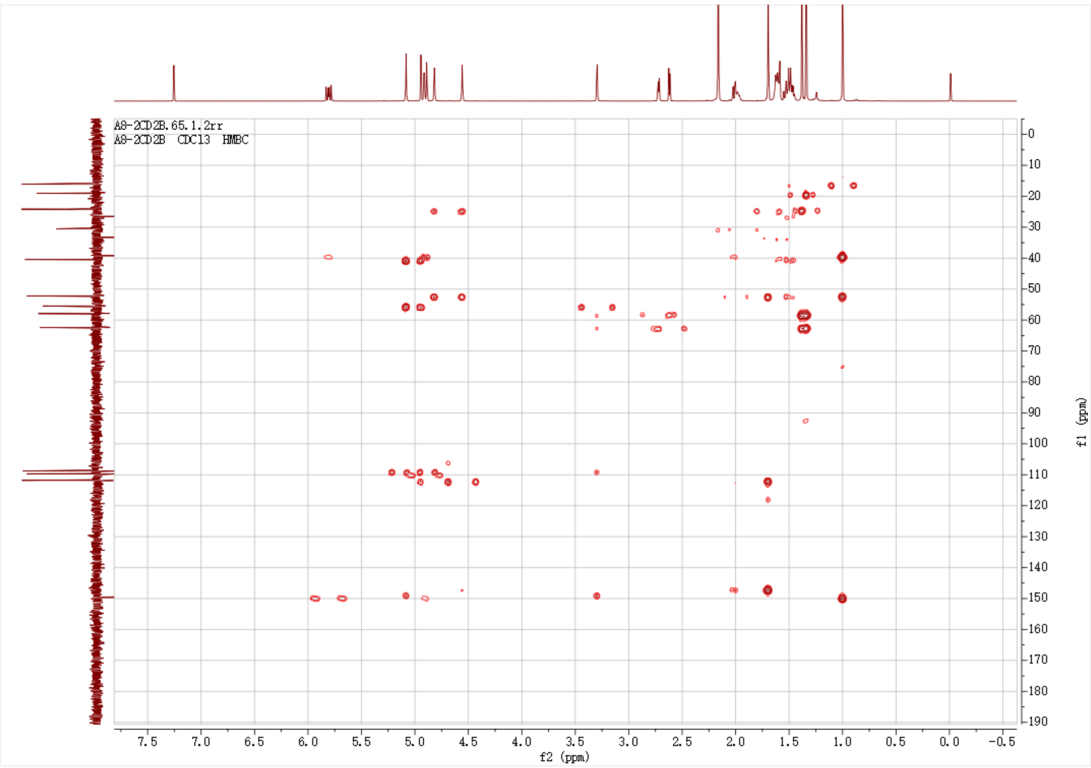

Figure S62. HMBC spectrum of **10** (600 MHz, $CDCl_3$)

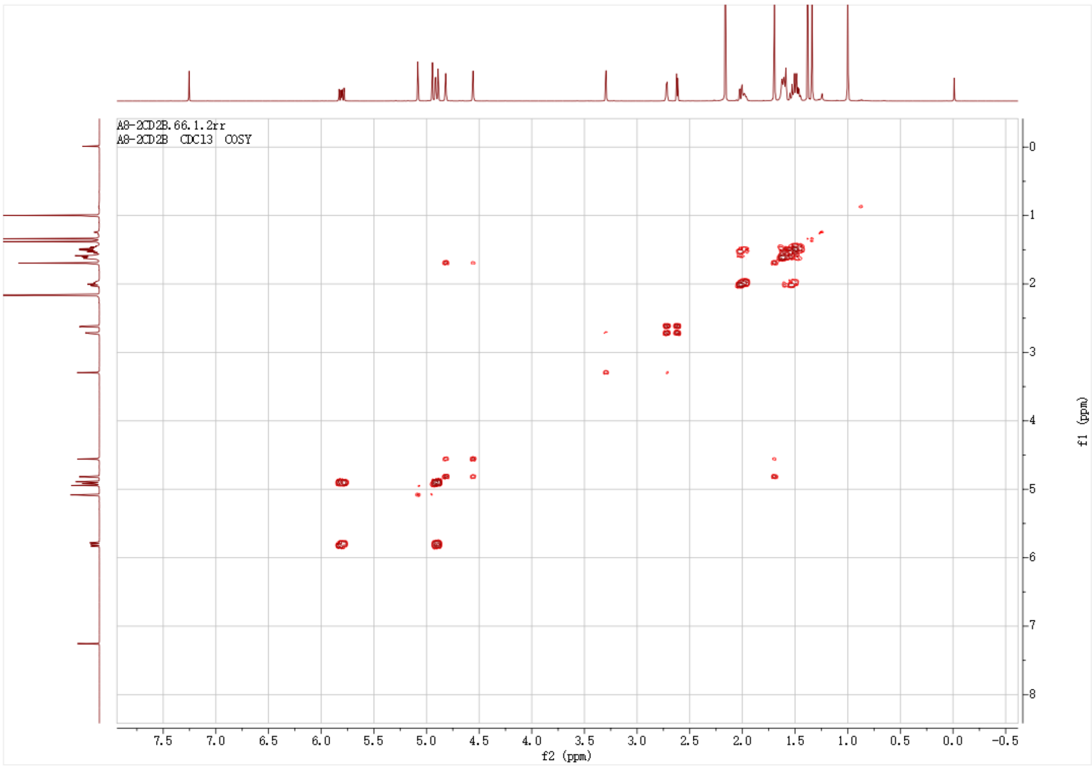

Figure S63. ¹H–¹H COSY spectrum of **10** (600 MHz, $CDCl_3$)

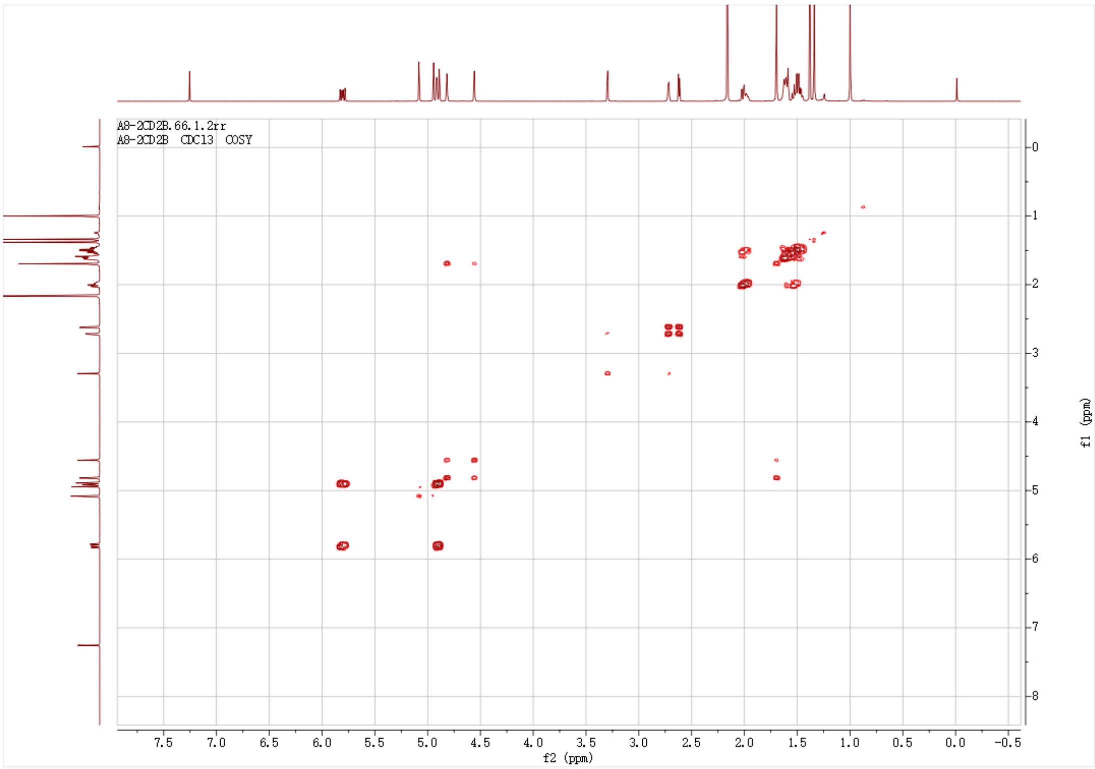

Figure S64. NOESY spectrum of **10** (600 MHz, $CDCl_3$)

Qualitative Analysis Report

**Data Filename**	ESIH202103081.d	**Sample Name**	A8-A8-1CB5-2
**Sample ID**		**Position**	P1-C5
**Instrument Name**	Agilent G6520 Q-TOF	**Acq Method**	20160322_MS_ESIH_POS_1min.m
**Acquired Time**	6/17/2021 19:08:05	**IRM Calibration Status**	Success
**DA Method**	small molecular data analysis method.m	**Comment**	ESIH by zhuzhenyun

User Spectra

Fragmentor Voltage	Collision Energy	Ionization Mode
175	0	ESI

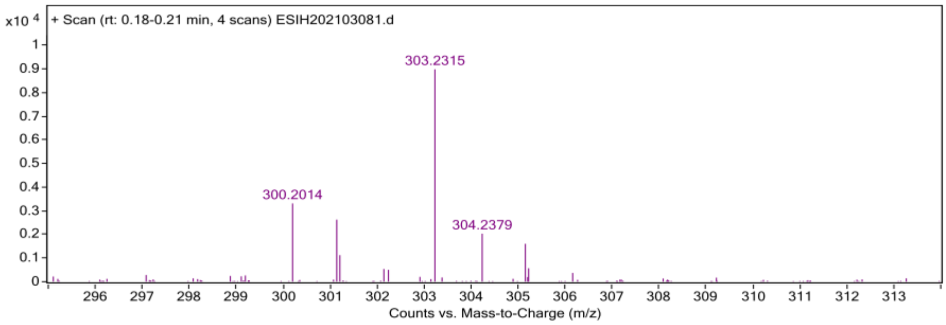

Formula Calculator Results

m/z	Calc m/z	Diff (mDa)	Diff (ppm)	Ion Formula	Ion
303.2315	303.2319	0.35	1.14	C20 H31 O2	(M+H)+

--- End Of Report ---

Figure S65. HRESIMS spectrum of **10**

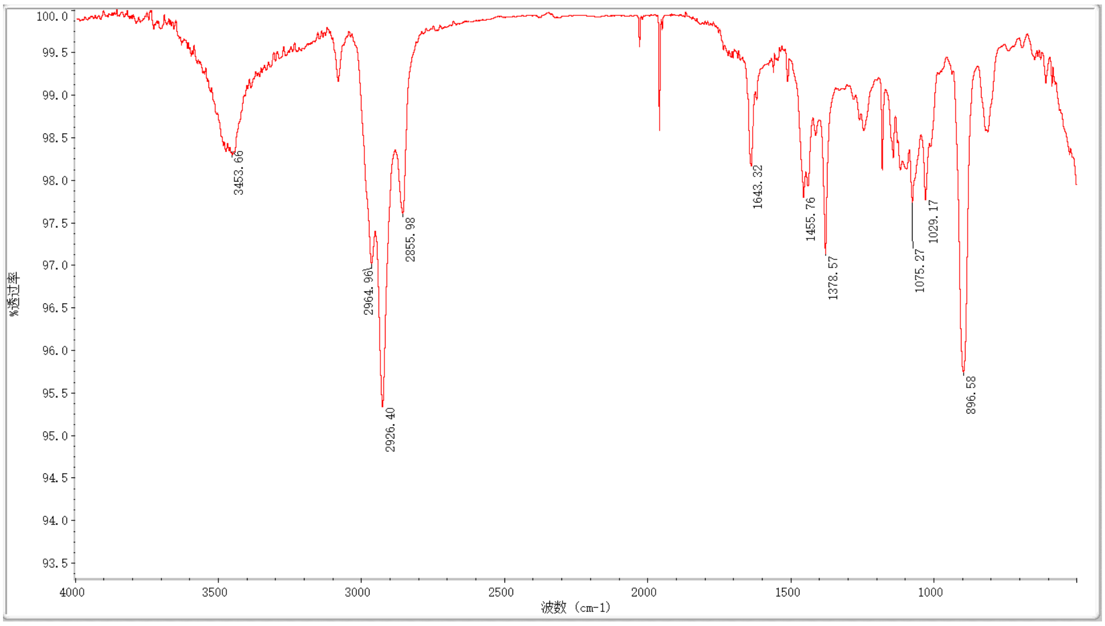

Figure S66. IR spectrum of **10**

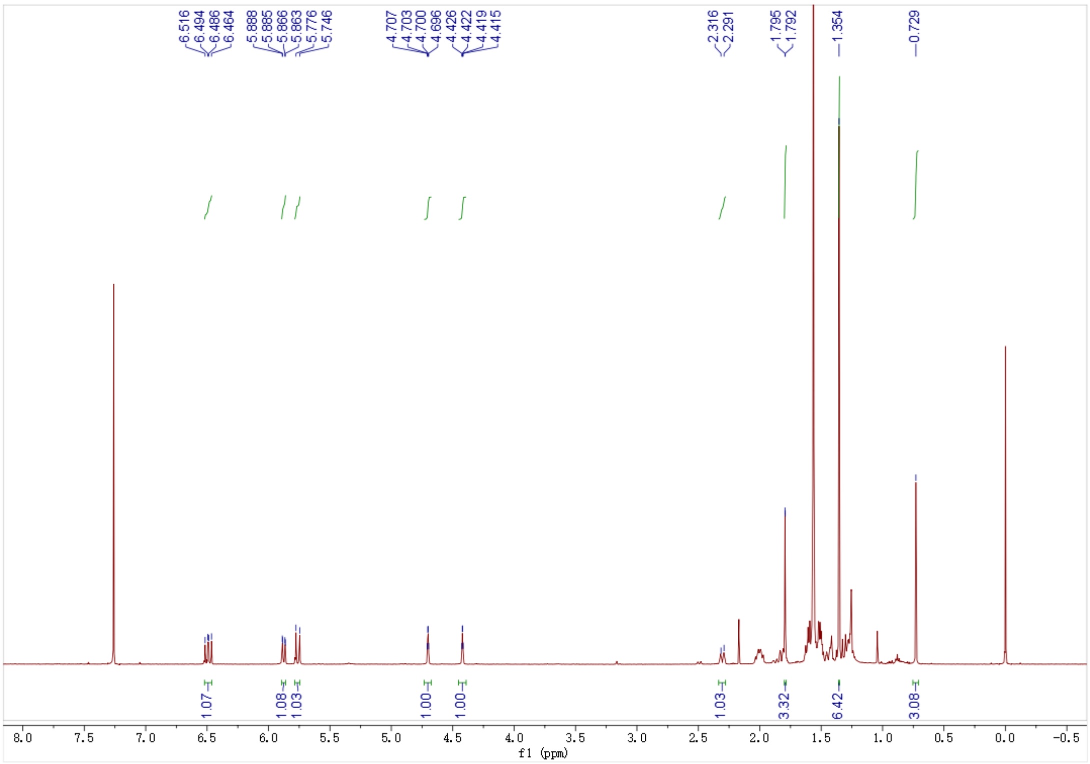

Figure S67. ^{1}H NMR spectrum of **11** (600MHz, $CDCl_3$)

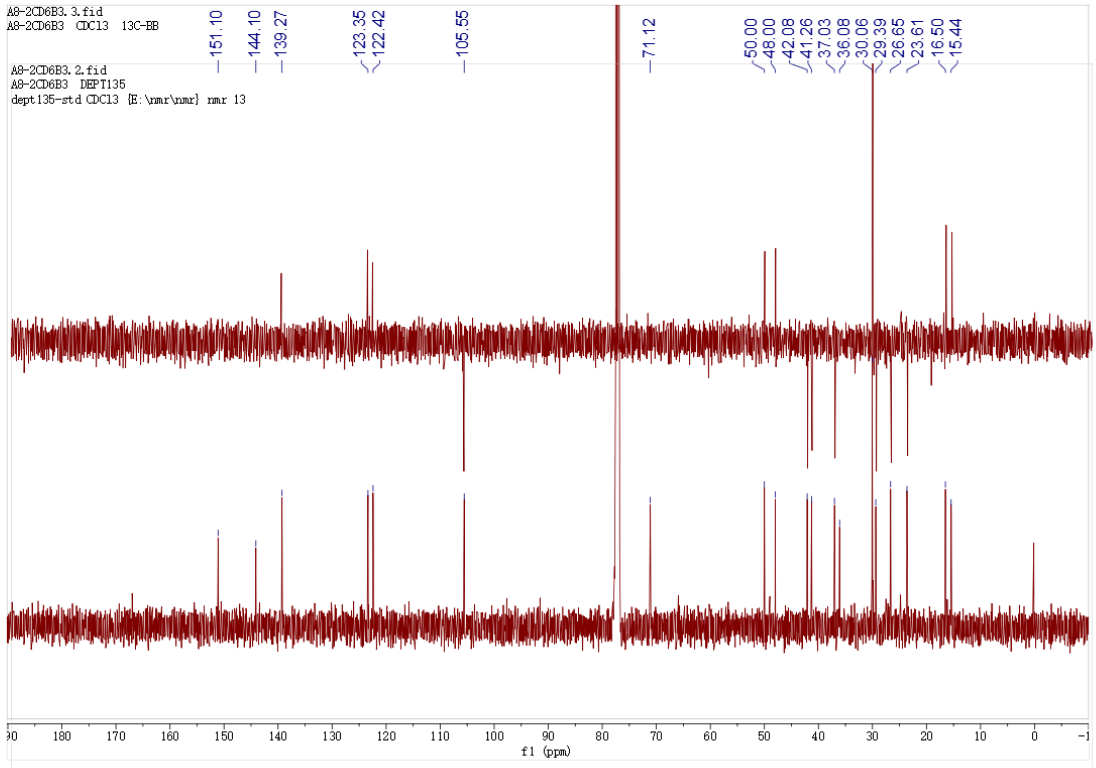

Figure S68. ^{13}C NMR spectrum of **11** (125 MHz, $CDCl_3$)

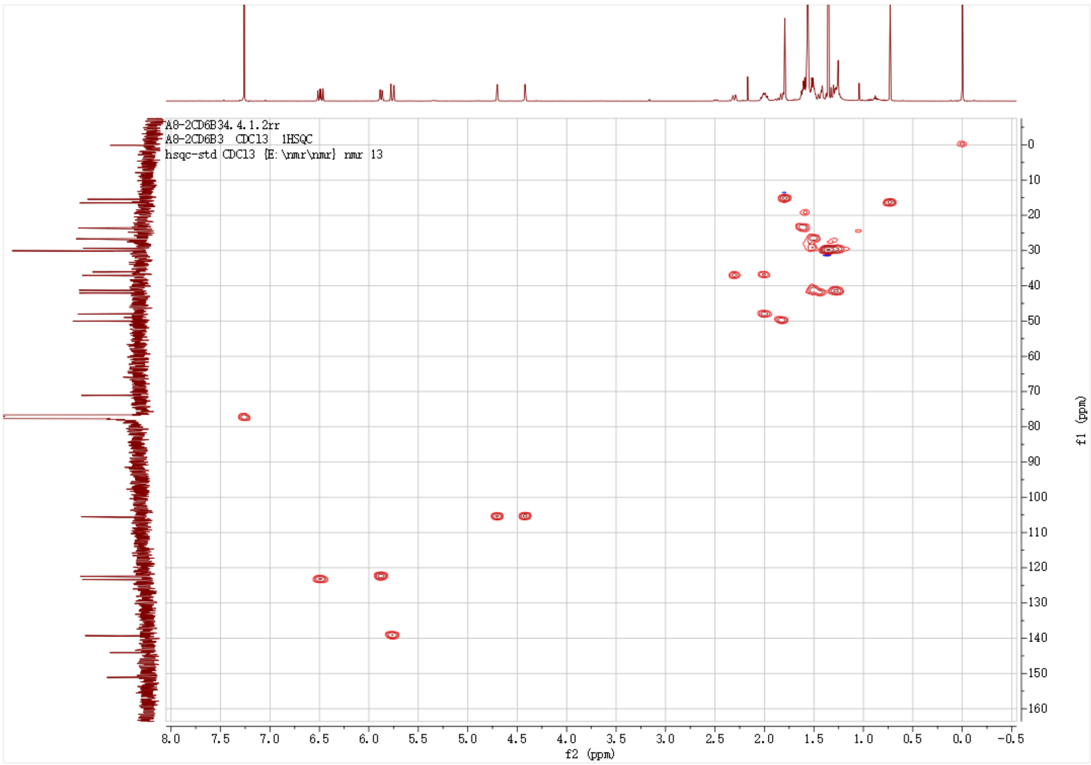

Figure S69. HSQC spectrum of **11** (600 MHz, CDCl3)

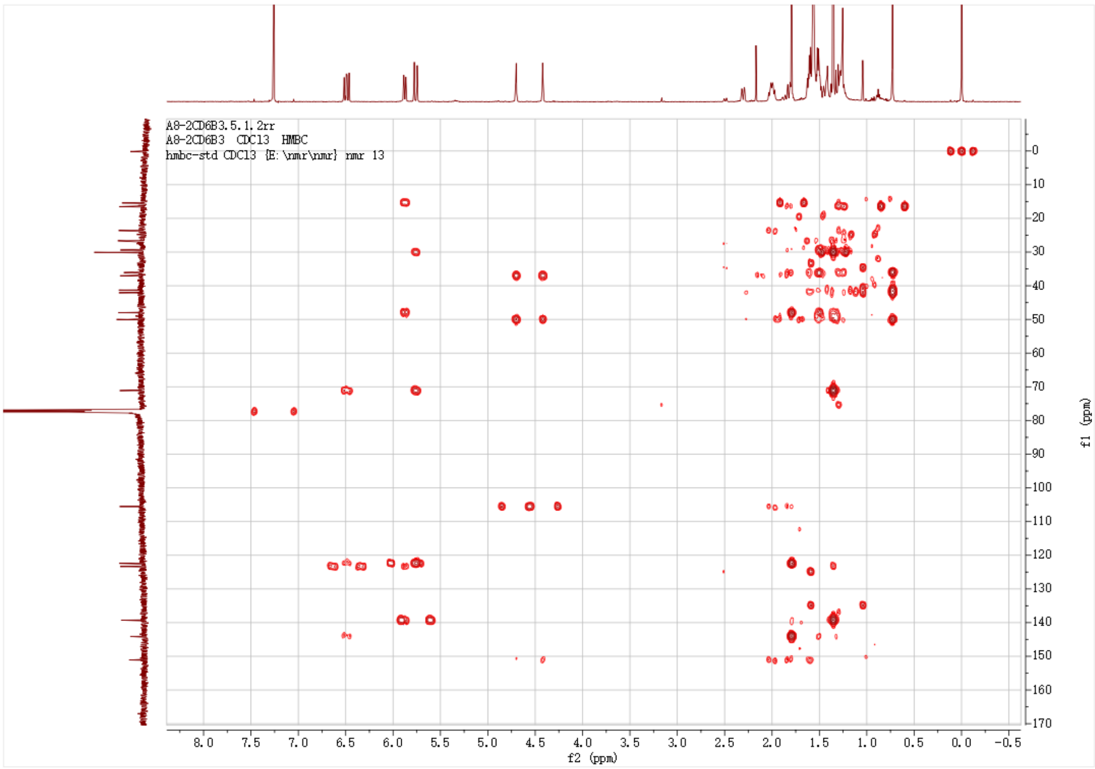

Figure S70. HMBC spectrum of **11** (600 MHz, CDCl3)

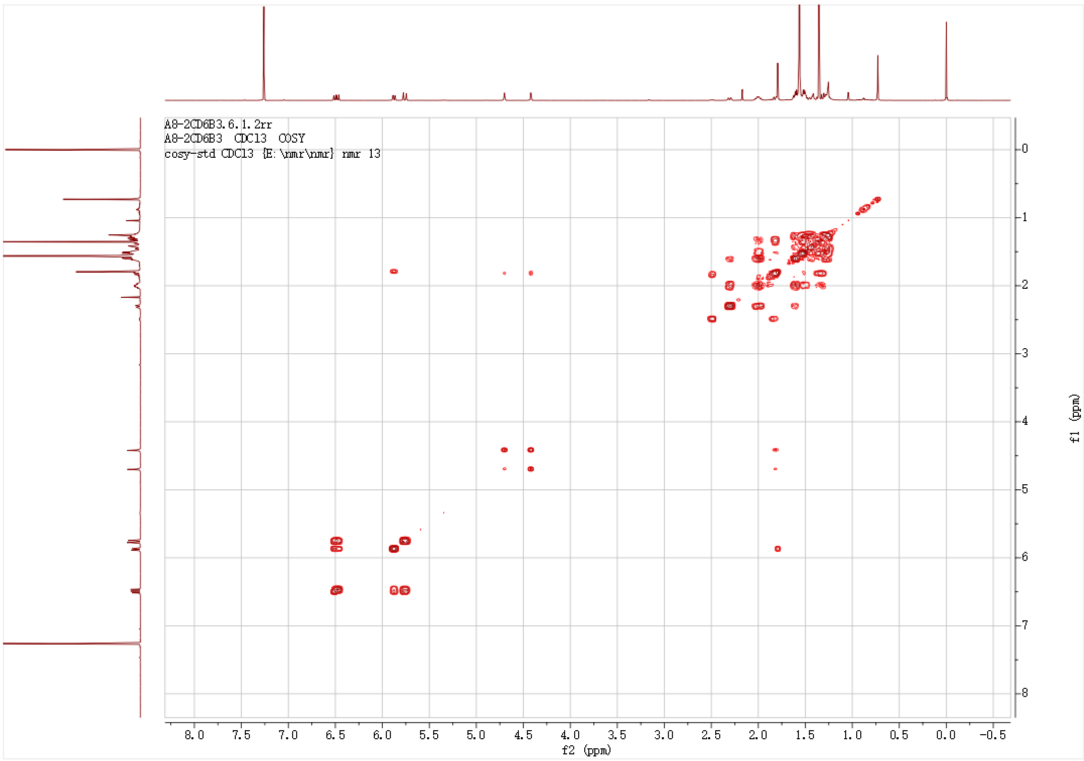

Figure S71. ^{1}H–^{1}H COSY spectrum of **11** (600 MHz, $CDCl_3$)

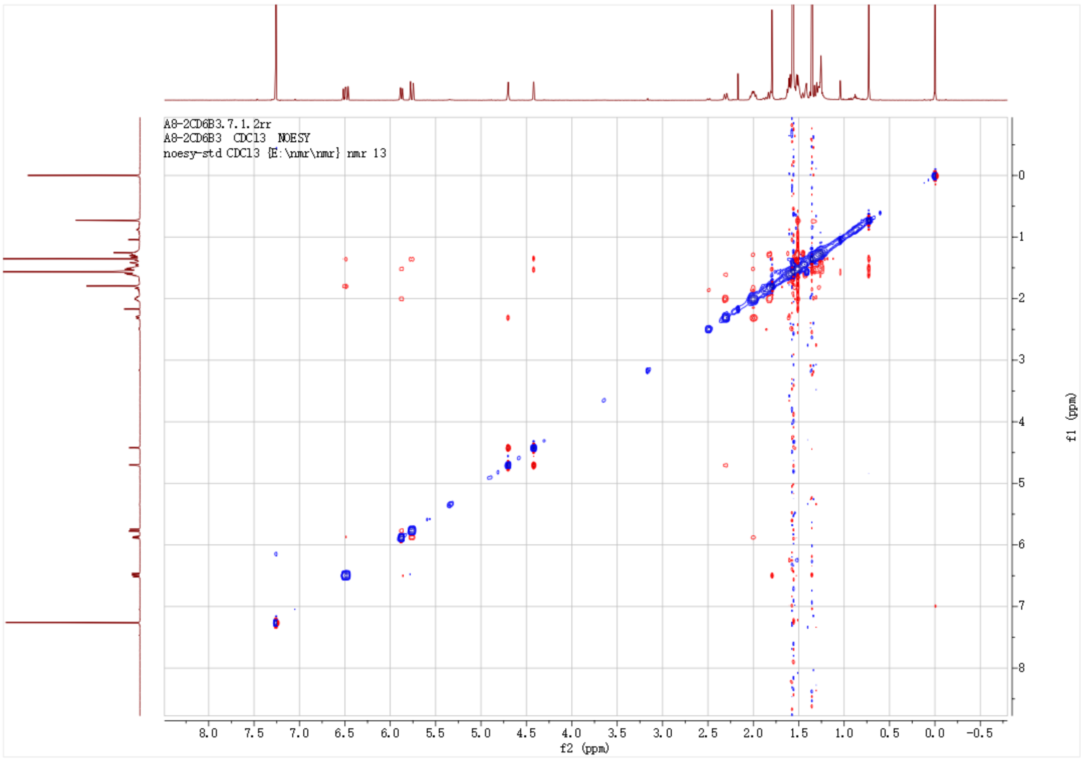

Figure S72. NOESY spectrum of **11** (600 MHz, $CDCl_3$)

D:\data\2021\EI202101757_A8-2CD6B3 -c1 10/11/2021 4:44:27 PM

EI202101757 A8-2CD6B3 -c1#7 RT: 1.24
T: + c EI Full ms [49.50-800.50]
m/z= 48-803

m/z	Intensity	Relative	Theo. Mass	Delta (mmu)	RDB equiv.	Composition
69.0696	2159378.0	10.91	69.0699	-0.28	1.5	C_5H_9
79.0177	5271272.0	26.64	79.0178	-0.11	4.5	$C_5H_3O_1$
80.0239	1806400.0	9.13	80.0257	-1.77	4.0	$C_5H_4O_1$
81.0312	6581477.0	33.26	81.0335	-2.30	3.5	$C_5H_5O_1$
93.0702	8451328.0	42.71	93.0699	0.35	3.5	C_7H_9
95.0878	8269501.0	41.79	95.0855	2.29	2.5	C_7H_{11}
105.0361	7764670.0	39.24	105.0335	2.61	5.5	$C_7H_5O_1$
111.0212	4003476.0	20.23	111.0229	-1.74	8.5	C_9H_3
119.0858	6925085.0	35.00	119.0855	0.28	4.5	C_9H_{11}
131.0856	2796667.0	14.13	131.0855	0.12	5.5	$C_{10}H_{11}$
133.1014	8804864.0	44.50	133.1012	0.18	4.5	$C_{10}H_{13}$
134.1076	2902759.0	14.67	134.1090	-1.40	4.0	$C_{10}H_{14}$
135.1166	3465007.0	17.51	135.1168	-0.22	3.5	$C_{10}H_{15}$
145.1010	4440810.0	22.44	145.1012	-0.15	5.5	$C_{11}H_{13}$
147.1167	13904640.0	70.27	147.1168	-0.17	4.5	$C_{11}H_{15}$
148.1221	3253231.0	16.44	148.1247	-2.53	4.0	$C_{11}H_{16}$
149.1315	2609460.0	13.19	149.1325	-0.99	3.5	$C_{11}H_{17}$
159.1147	4436779.0	22.42	159.1168	-2.14	5.5	$C_{12}H_{15}$
160.1219	2032857.0	10.27	160.1247	-2.73	5.0	$C_{12}H_{16}$
161.1305	14538752.0	73.47	161.1325	-1.97	4.5	$C_{12}H_{17}$
162.1380	11487232.0	58.05	162.1403	-2.31	4.0	$C_{12}H_{18}$
163.1452	3687488.0	18.63	163.1481	-2.93	3.5	$C_{12}H_{19}$
173.1324	3984464.0	20.14	173.1325	-0.12	5.5	$C_{13}H_{17}$
175.1479	3621926.0	18.30	175.1481	-0.21	4.5	$C_{13}H_{19}$
187.1482	3997293.0	20.20	187.1481	0.11	5.5	$C_{14}H_{19}$
188.1552	3067359.0	15.50	188.1560	-0.77	5.0	$C_{14}H_{20}$
189.1633	19788288.0	100.00	189.1638	-0.51	4.5	$C_{14}H_{21}$
199.1476	2196013.0	11.10	199.1481	-0.54	6.5	$C_{15}H_{19}$
201.1635	6774948.0	34.24	201.1638	-0.26	5.5	$C_{15}H_{21}$
215.1779	4036843.0	20.40	215.1794	-1.50	5.5	$C_{16}H_{23}$
216.1857	10152704.0	51.31	216.1873	-1.55	5.0	$C_{16}H_{24}$
227.1796	2300580.0	11.63	227.1794	0.15	6.5	$C_{17}H_{23}$
255.2101	6456835.0	32.63	255.2107	-0.61	6.5	$C_{19}H_{27}$
270.2343	6491346.0	32.80	270.2342	0.10	6.0	$C_{20}H_{30}$
271.2398	2065761.0	10.44	271.2420	-2.23	5.5	$C_{20}H_{31}$
273.2213	7769219.0	39.26	273.2213	-0.01	5.5	$C_{19}H_{29}O_1$
288.2443	19262976.0	97.35	288.2448	-0.47	5.0	$C_{20}H_{32}O_1$

Figure S73. HREIMS spectrum of **11**

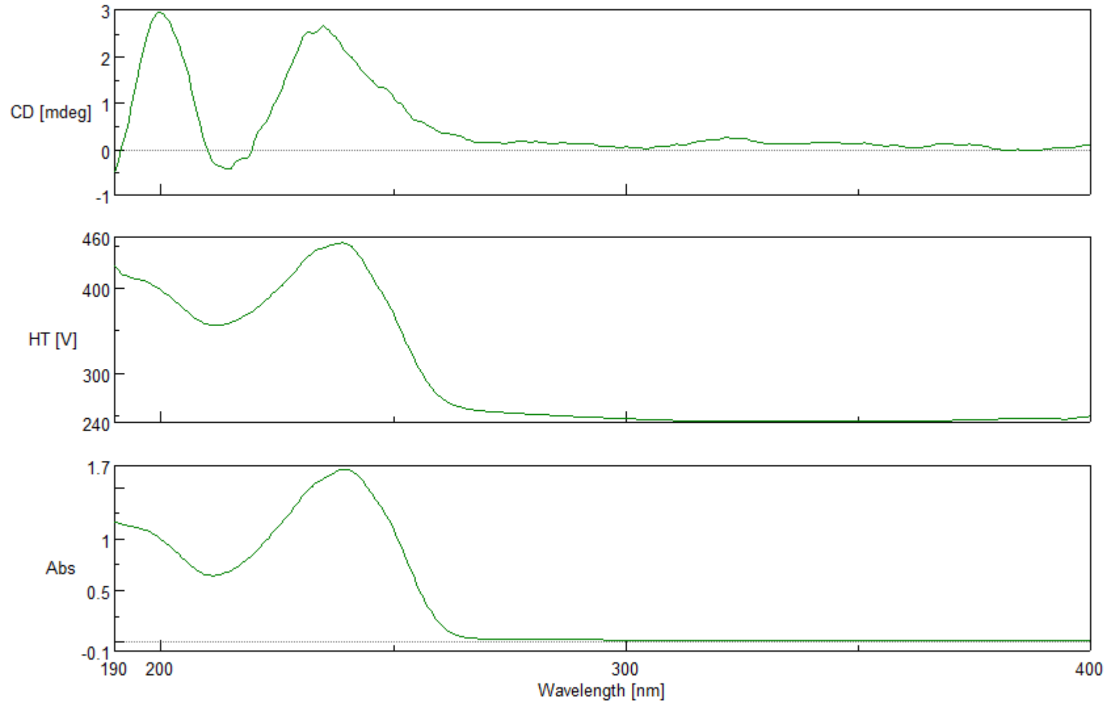

Figure S74. UV and CD spectrum of **11**

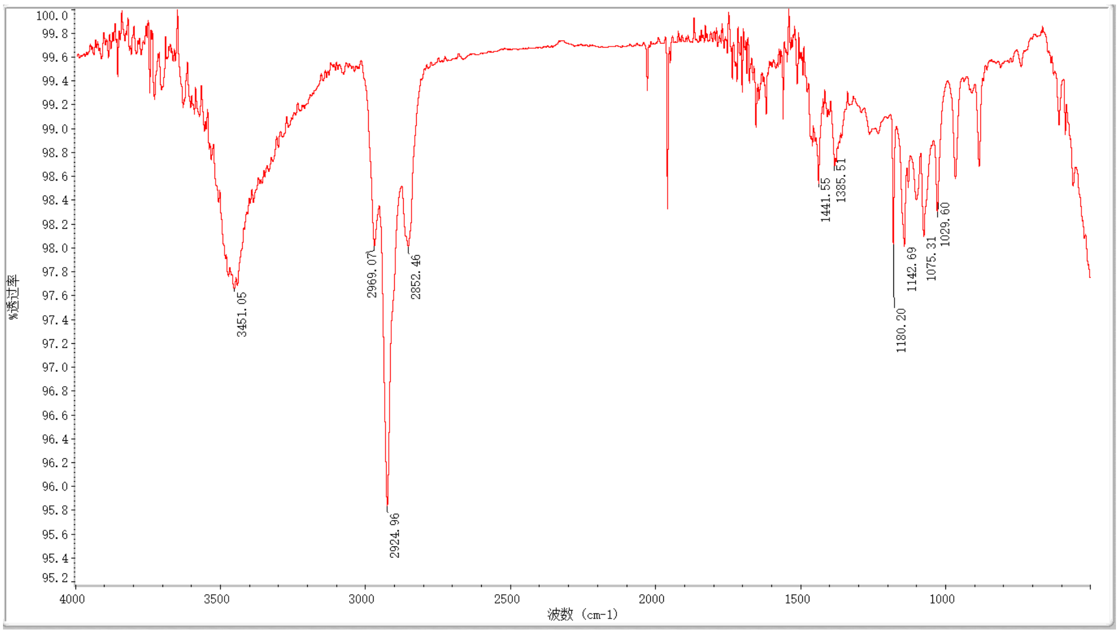

Figure S75. IR spectrum of **11**

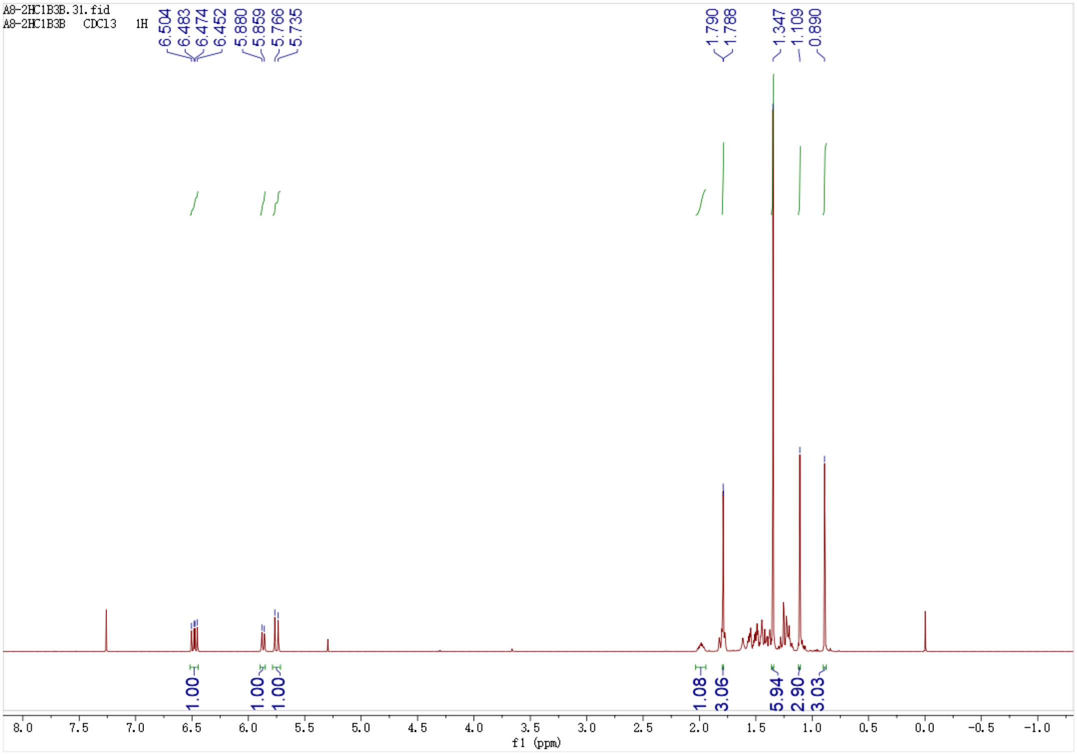

Figure S76. ^{1}H NMR spectrum of **12** (600MHz, $CDCl_3$)

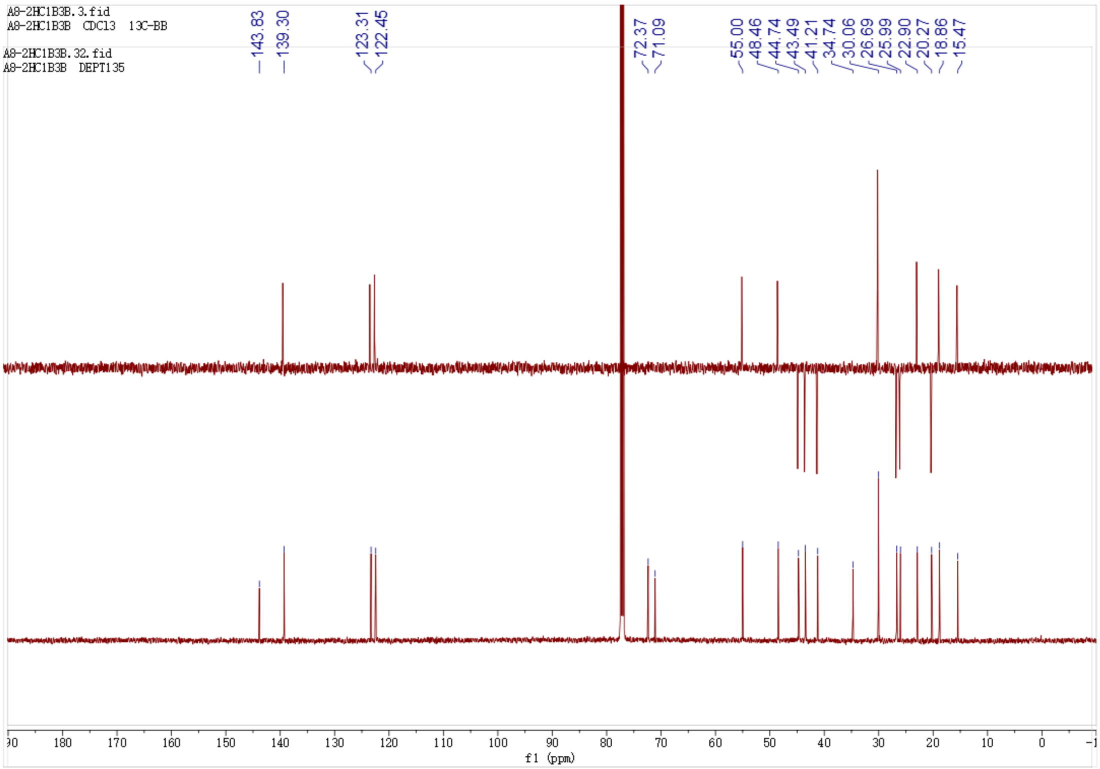

Figure S77. ^{13}C NMR spectrum of **12** (125 MHz, $CDCl_3$)

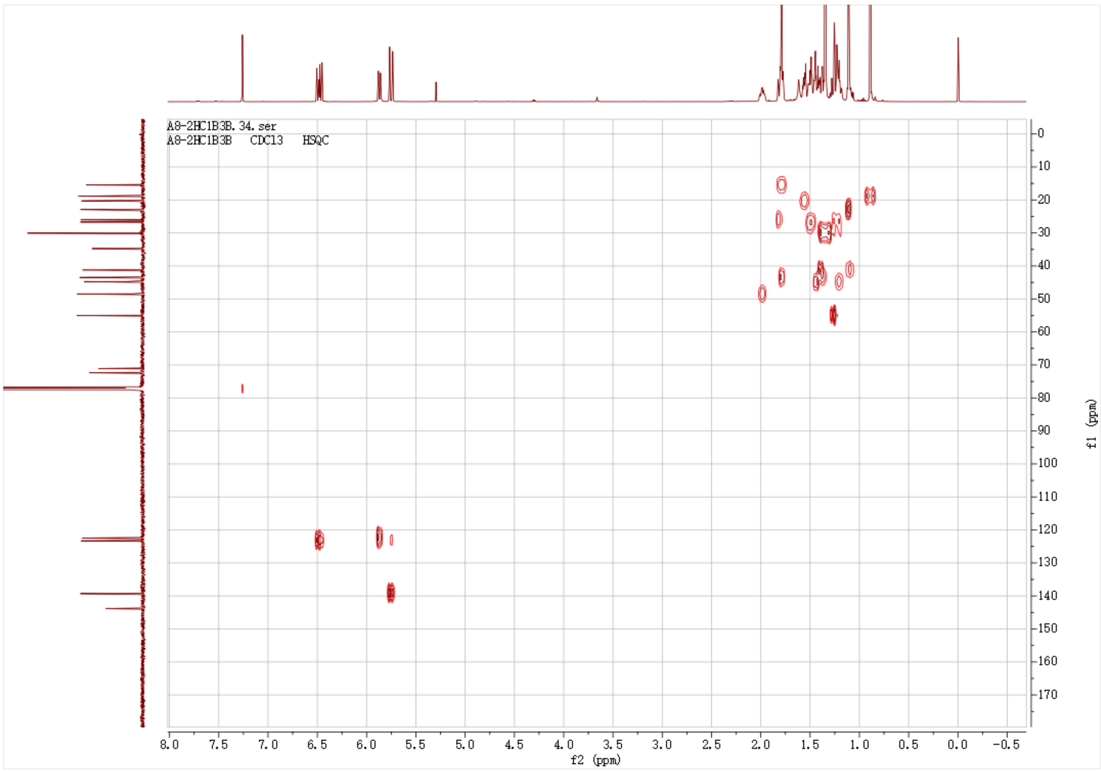

Figure S78. HSQC spectrum of **12** (600 MHz, $CDCl_3$)

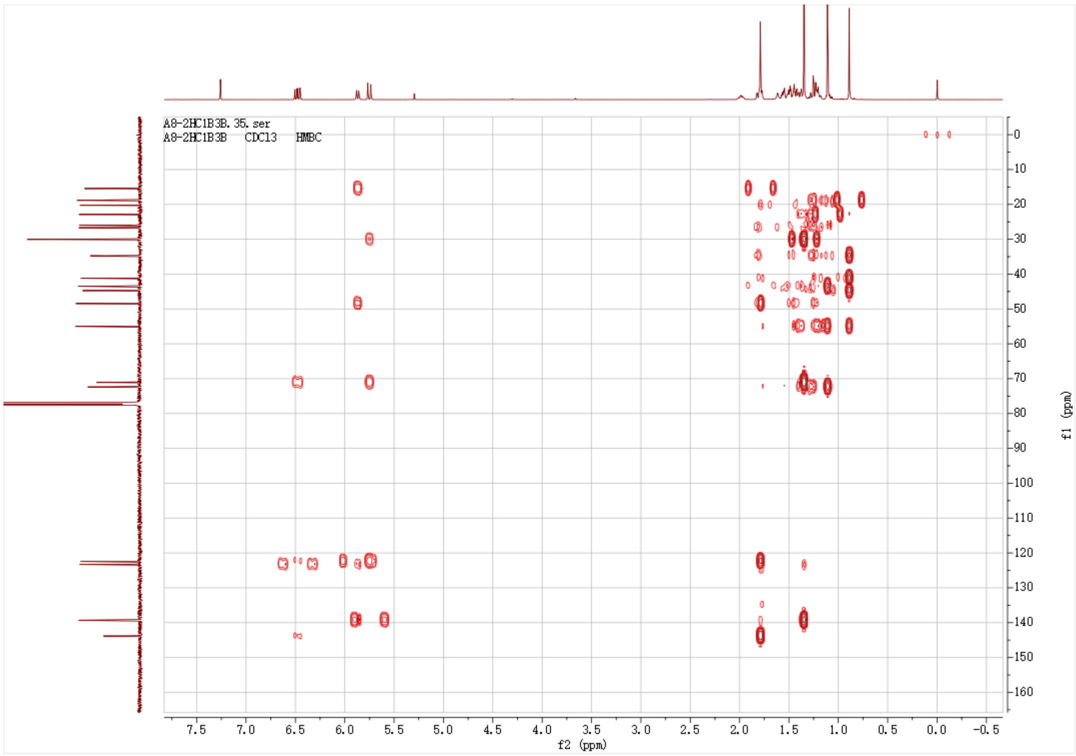

Figure S79. HMBC spectrum of **12** (600 MHz, $CDCl_3$)

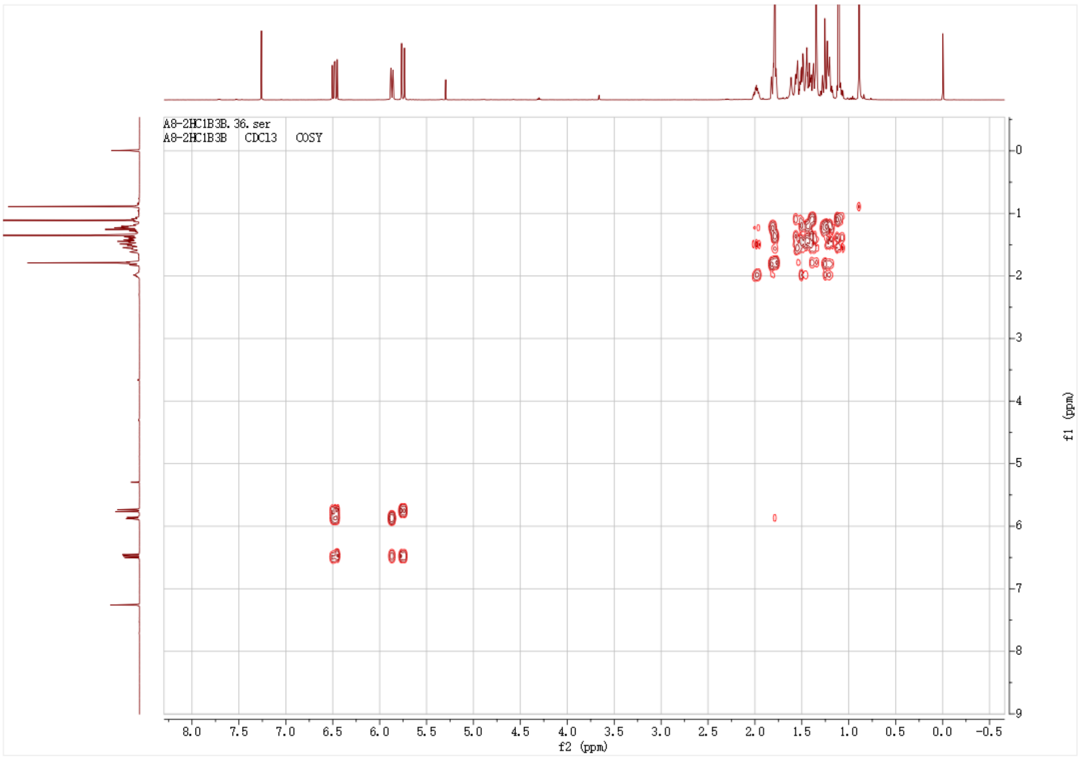

Figure S80. ^1H–^1H COSY spectrum of **12** (600 MHz, $CDCl_3$)

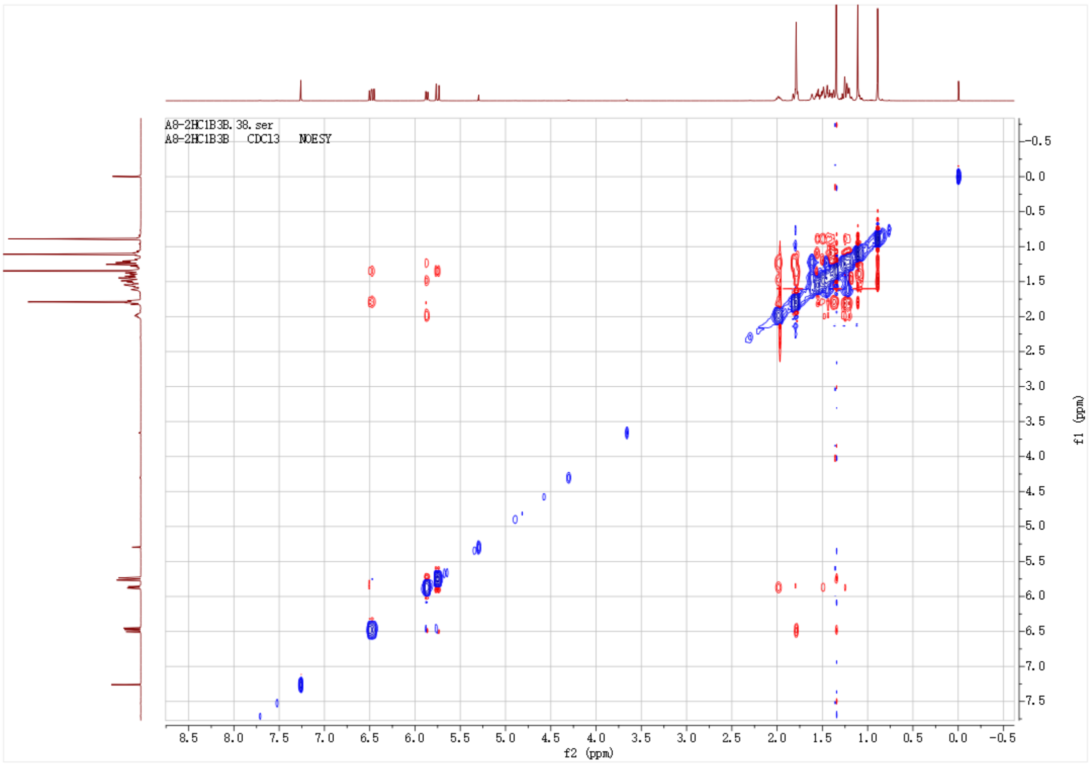

Figure S81. NOESY spectrum of **12** (600 MHz, $CDCl_3$)

D:\data\2021\EI202101774_-A8-2HC1B3B -c1   10/20/2021 12:29:00 PM

EI202101774_-A8-2HC1B3B -c1#14 RT: 2.68
T: + c EI Full ms [49.50-800.50]
m/z= 48-803

m/z	Intensity	Relative	Theo. Mass	Delta (mmu)	RDB equiv.	Composition
59.0103	2524197.0	10.65	59.0128	-2.42	1.5	$C_2H_3O_2$
162.0071	4810107.0	20.30	162.0100	-2.93	12.0	$C_{12}H_2O_1$
176.9954	1557874.0	6.58	176.9971	-1.73	12.5	$C_{12}H_1O_2$
245.2269	6149729.0	25.96	245.2264	0.57	4.5	$C_{18}H_{29}$
255.2113	2755052.0	11.63	255.2107	0.61	6.5	$C_{19}H_{27}$
270.2337	1391885.0	5.87	270.2342	-0.46	6.0	$C_{20}H_{30}$
273.2204	5979248.0	25.24	273.2213	-0.87	5.5	$C_{19}H_{29}O_1$
288.2453	6068558.0	25.61	288.2448	0.55	5.0	$C_{20}H_{32}O_1$
289.2497	1726042.0	7.29	289.2526	-2.88	4.5	$C_{20}H_{33}O_1$
306.2553	3863438.0	16.31	306.2553	-0.08	4.0	$C_{20}H_{34}O_2$

Figure S82. HREIMS spectrum of **12**

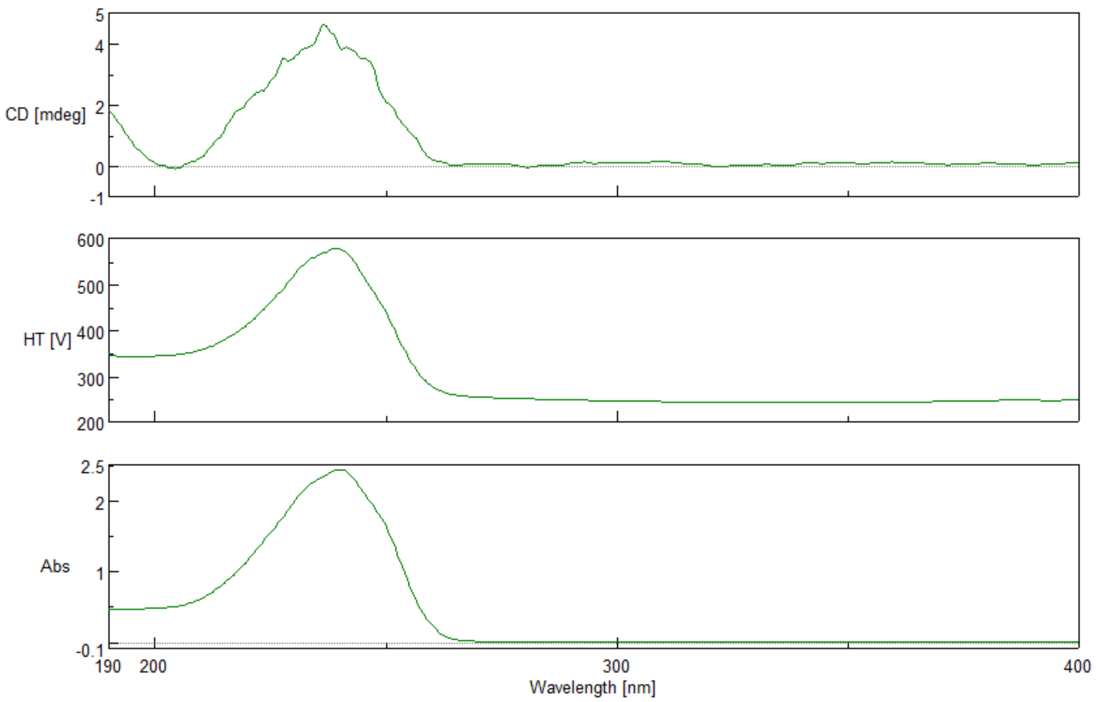

Figure S83. UV and CD spectrum of **12**

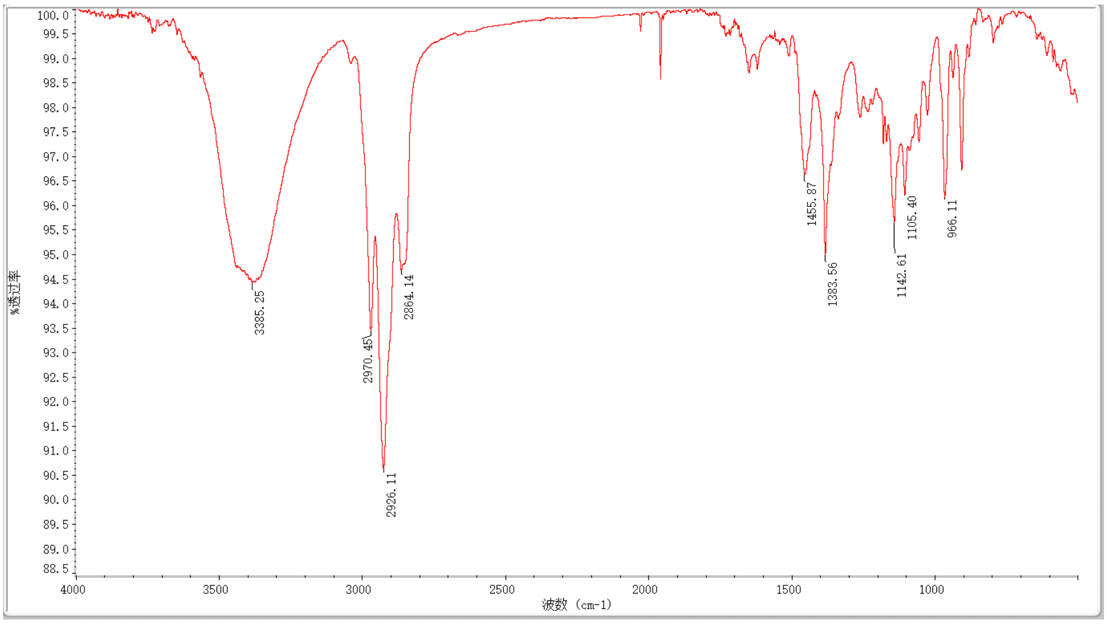

Figure S84. IR spectrum of **12**

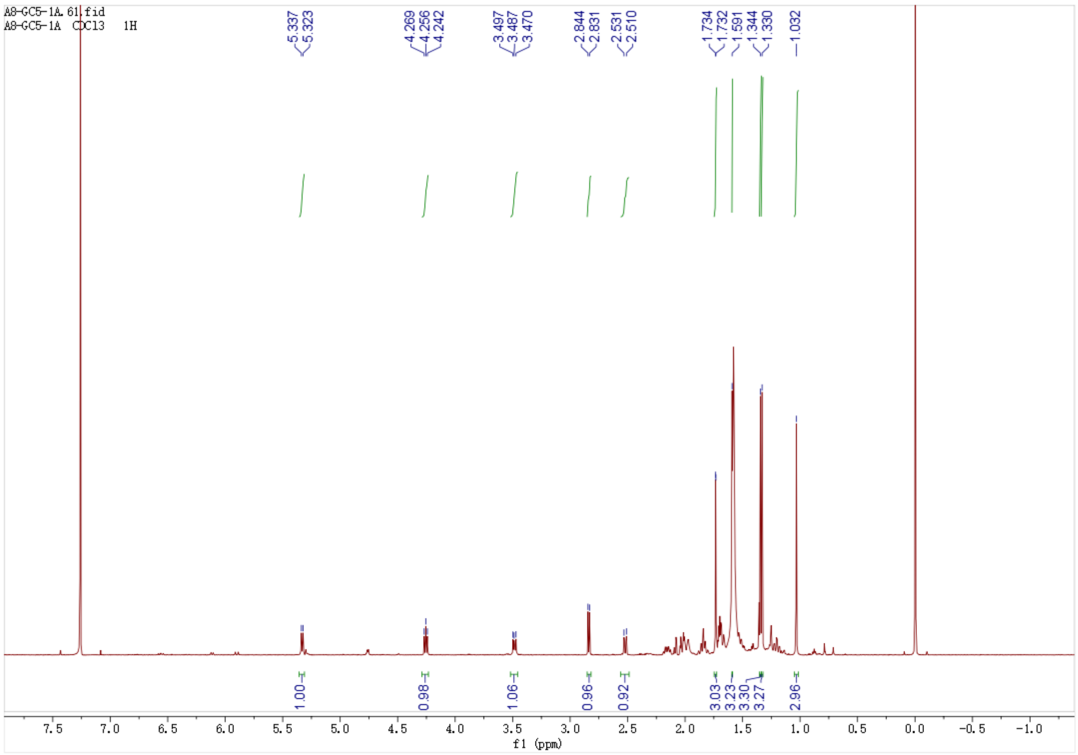

Figure S85. ^{1}H NMR spectrum of **13** (600MHz, $CDCl_3$)

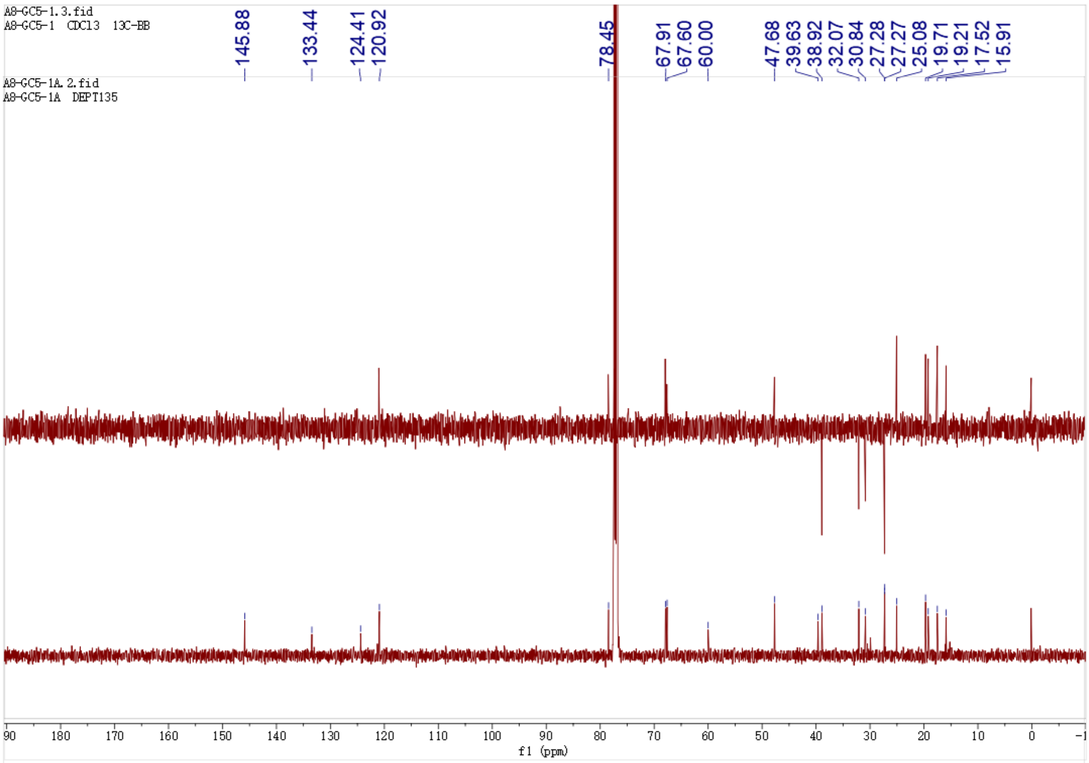

Figure S86. ^{13}C NMR spectrum of **13** (125 MHz, $CDCl_3$)

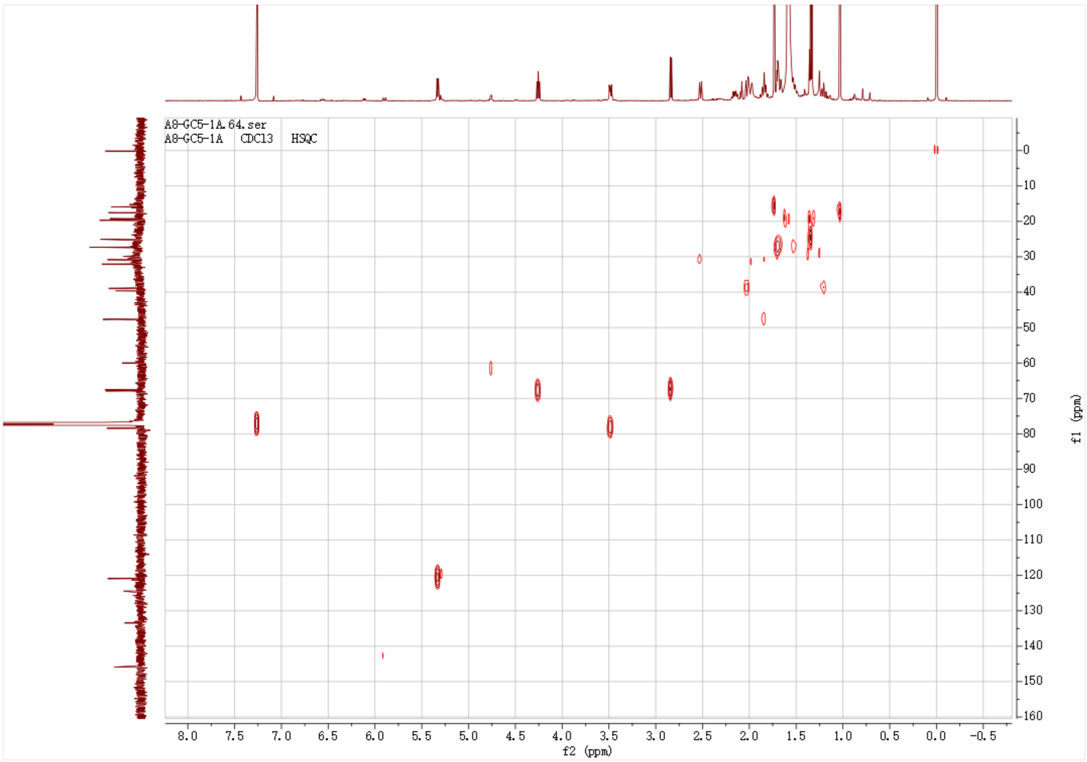

Figure S87. HSQC spectrum of **13** (600 MHz, $CDCl_3$)

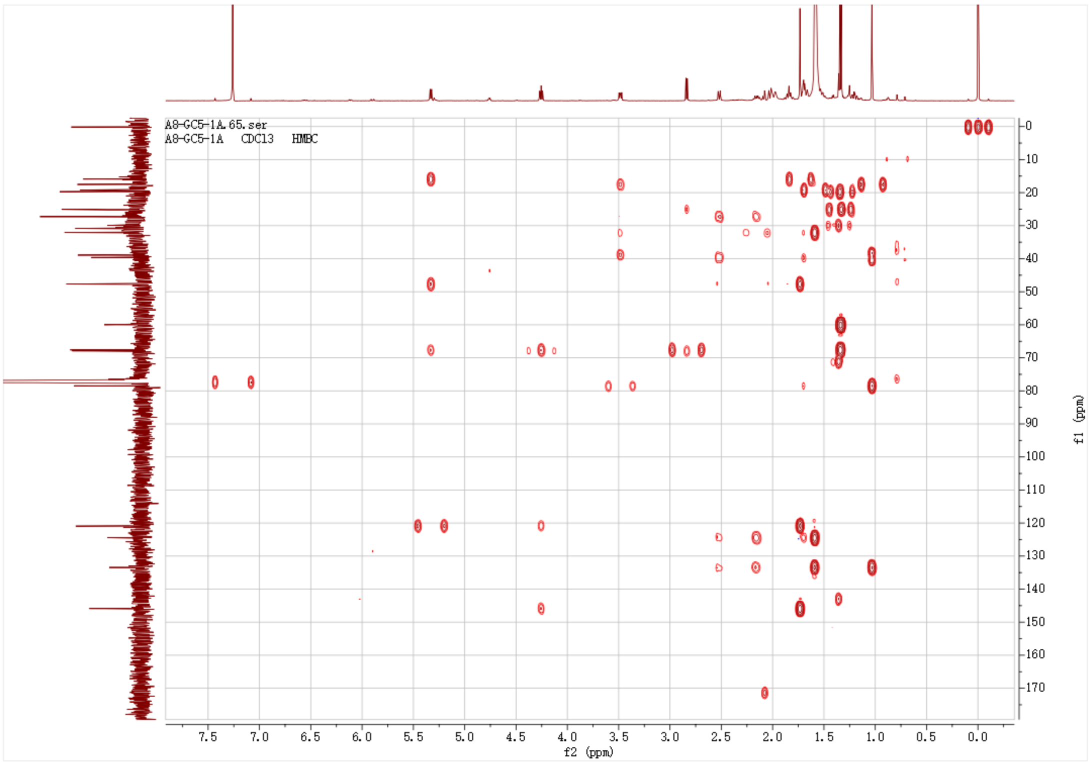

Figure S88. HMBC spectrum of **13** (600 MHz, $CDCl_3$)

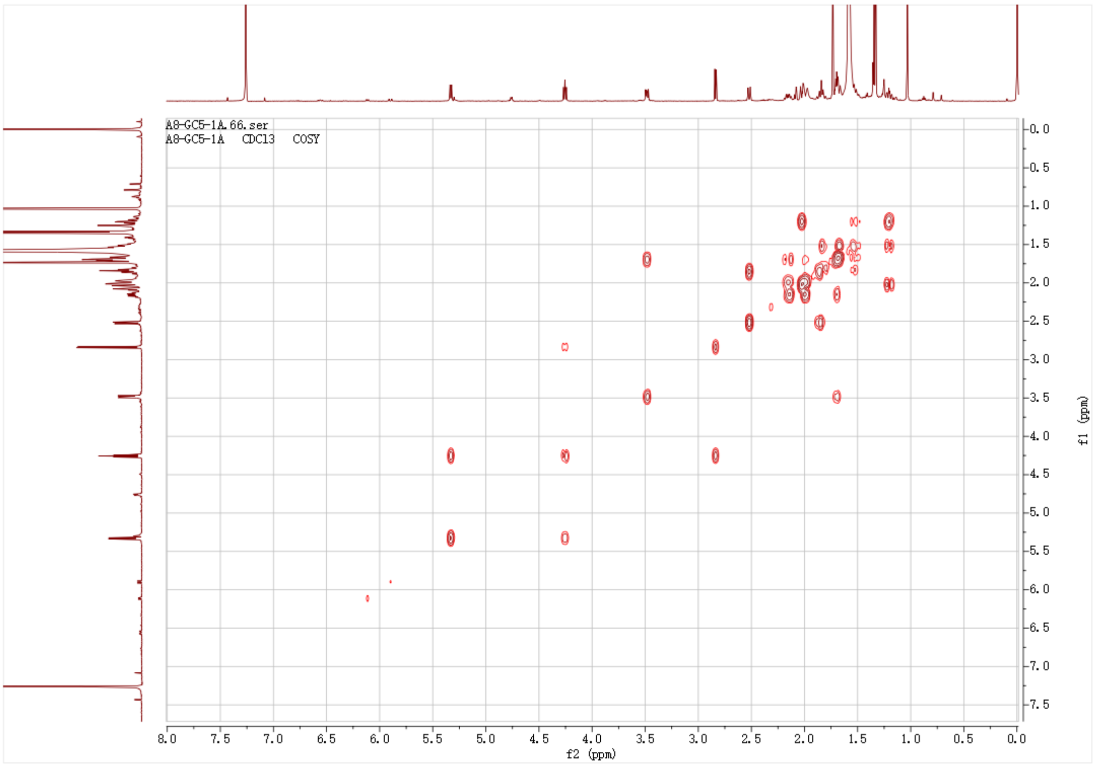

Figure S89. ${}^{1}H$–${}^{1}H$ COSY spectrum of **13** (600 MHz, $CDCl_3$)

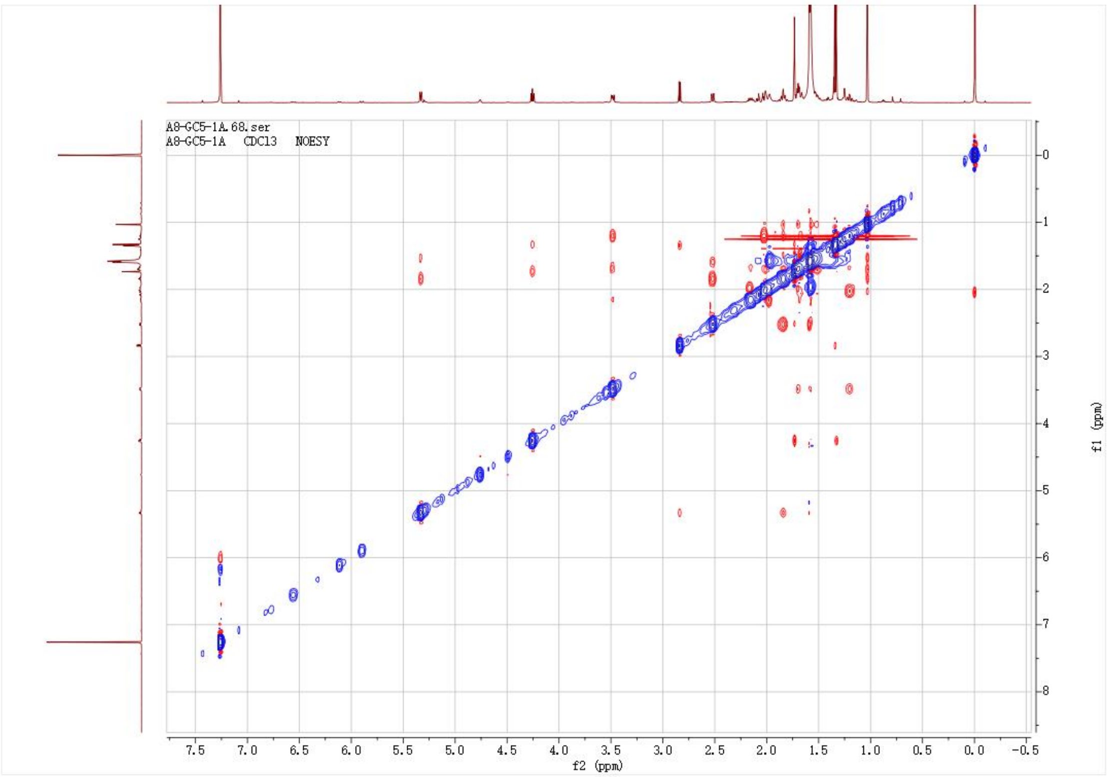

Figure S90. NOESY spectrum of **13** (600 MHz, $CDCl_3$)

D:\data\2021\EI202101749_A8-GC5-1A -c1 10/11/2021 3:14:34 PM

EI202101749_A8-GC5-1A -c1#14 RT: 2.68
T: + c EI Full ms [49.50-800.50]
m/z= 48-803

m/z	Intensity	Relative	Theo. Mass	Delta (mmu)	RDB equiv.	Composition
212.1546	1397829.0	4.66	212.1560	-1.35	7.0	$C_{16}H_{20}$
213.1626	2581159.0	8.60	213.1638	-1.14	6.5	$C_{16}H_{21}$
215.1426	1684101.0	5.61	215.1430	-0.43	6.5	$C_{15}H_{19}O_{1}$
218.1657	812947.0	2.71	218.1665	-0.78	5.0	$C_{15}H_{22}O_{1}$
229.1579	841138.0	2.80	229.1587	-0.82	6.5	$C_{16}H_{21}O_{1}$
230.1657	2084501.0	6.94	230.1665	-0.82	6.0	$C_{16}H_{22}O_{1}$
231.1729	1604021.0	5.34	231.1743	-1.45	5.5	$C_{16}H_{23}O_{1}$
244.1823	1308243.0	4.36	244.1822	0.10	6.0	$C_{17}H_{24}O_{1}$
248.1773	1264526.0	4.21	248.1771	0.22	5.0	$C_{16}H_{24}O_{2}$
249.1839	932739.0	3.11	249.1849	-0.96	4.5	$C_{16}H_{25}O_{2}$
259.1698	870855.0	2.90	259.1693	0.51	6.5	$C_{17}H_{23}O_{2}$
269.1890	787152.0	2.62	269.1900	-0.96	7.5	$C_{19}H_{25}O_{1}$
284.2138	1668902.0	5.56	284.2135	0.32	7.0	$C_{20}H_{28}O_{1}$
287.2004	1104993.0	3.68	287.2006	-0.16	6.5	$C_{19}H_{27}O_{2}$
302.2240	1388705.0	4.63	302.2240	-0.06	6.0	$C_{20}H_{30}O_{2}$
320.2347	1207871.0	4.02	320.2346	0.15	5.0	$C_{20}H_{32}O_{3}$

Figure S91. HREIMS spectrum of **13**

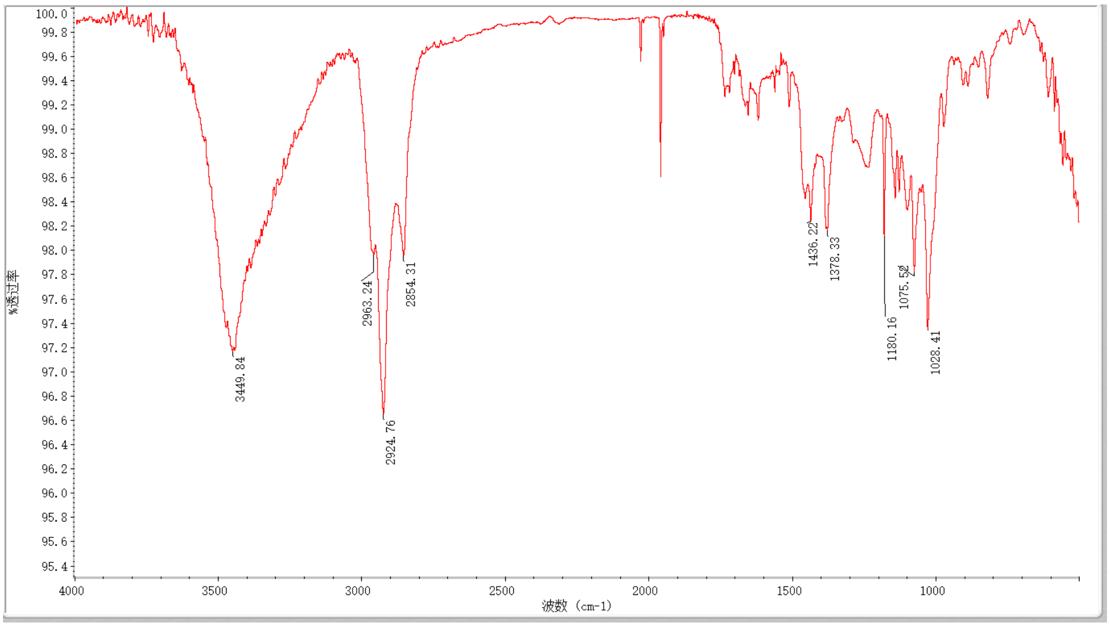

Figure S92. IR spectrum of **13**

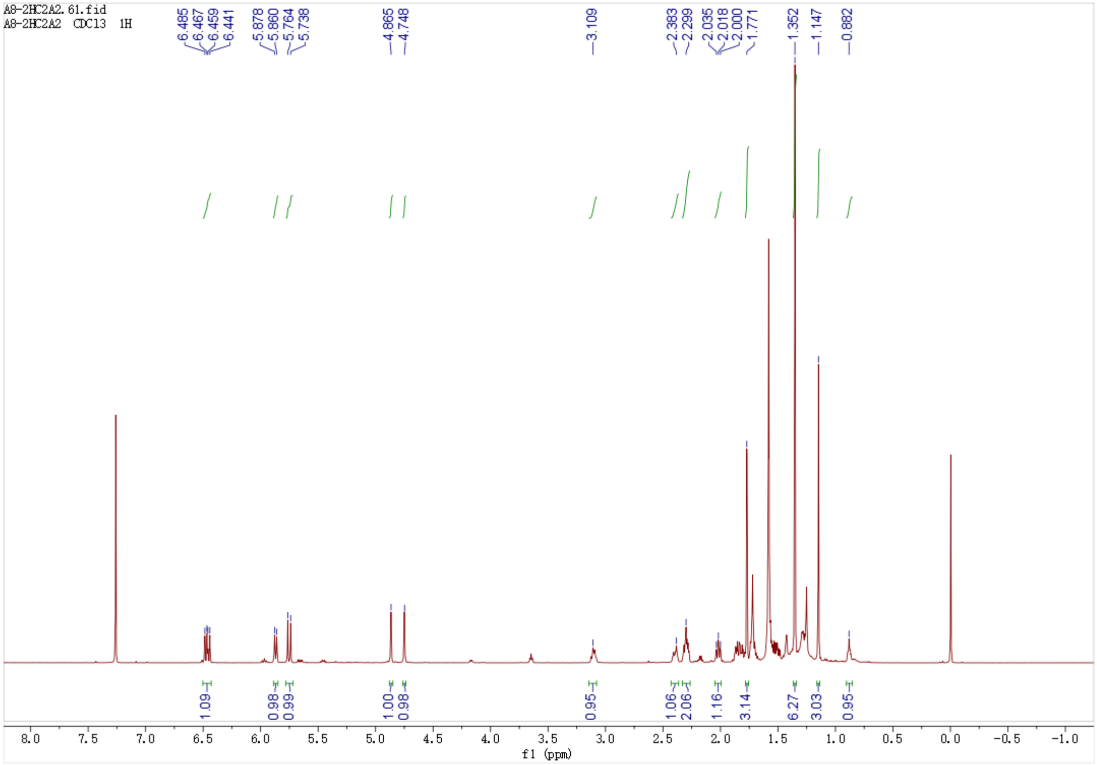

Figure S93. ^{1}H NMR spectrum of **14** (600MHz, $CDCl_3$)

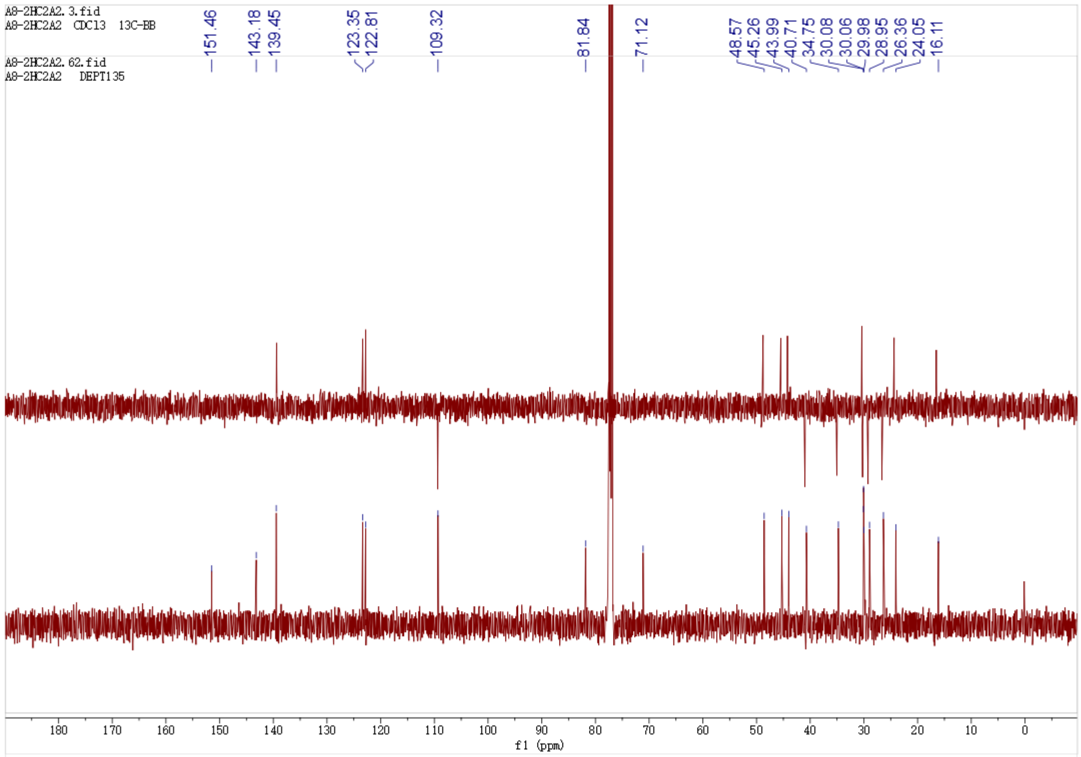

Figure S94. ^{13}C NMR spectrum of **14** (125 MHz, $CDCl_3$)

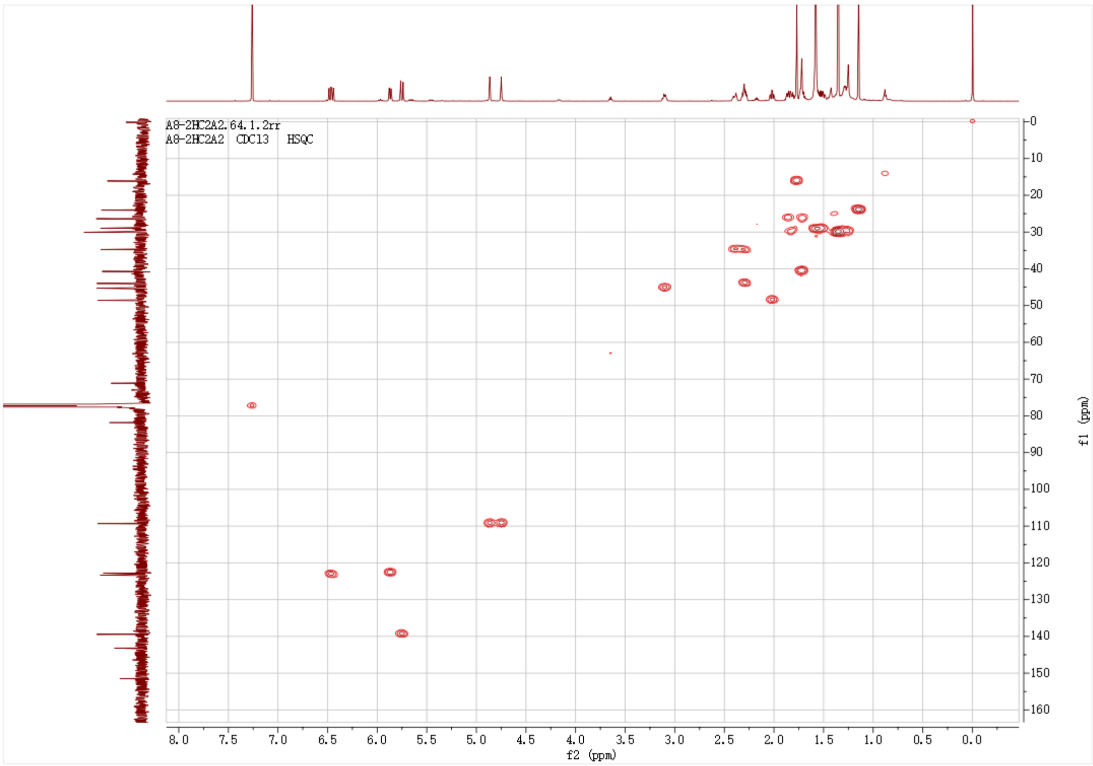

Figure S95. HSQC spectrum of **14** (600 MHz, $CDCl_3$)

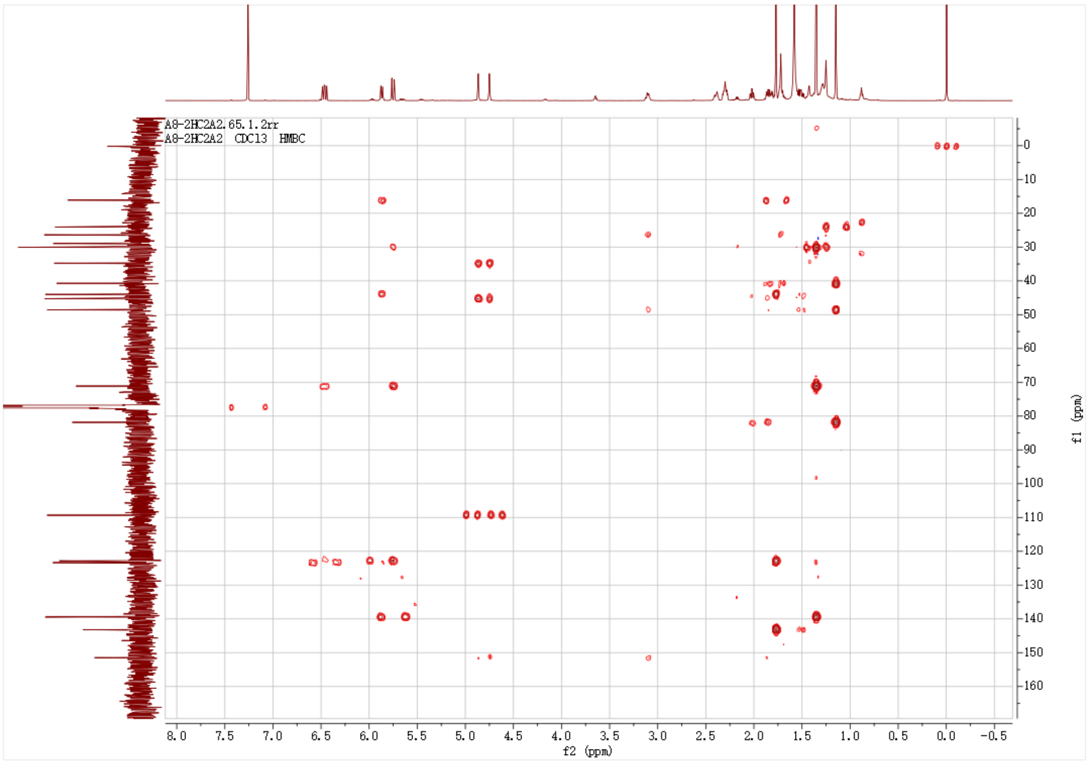

Figure S96. HMBC spectrum of **14** (600 MHz, $CDCl_3$)

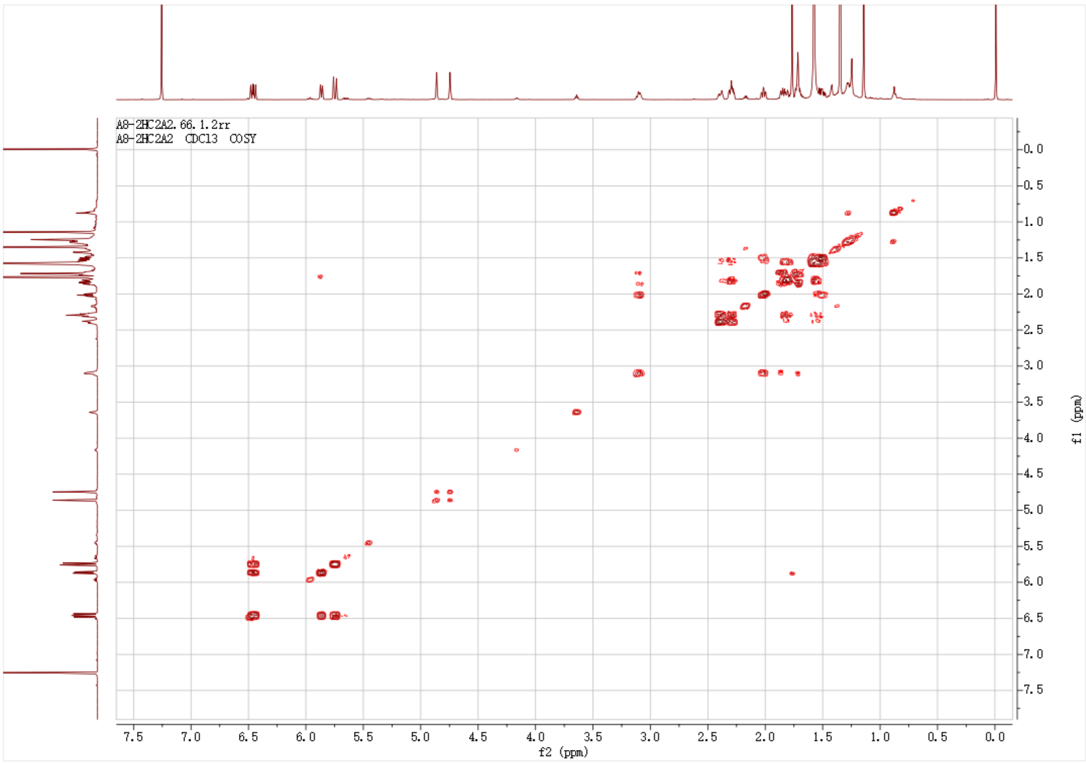

Figure S97. ^{1}H–^{1}H COSY spectrum of **14** (600 MHz, $CDCl_3$)

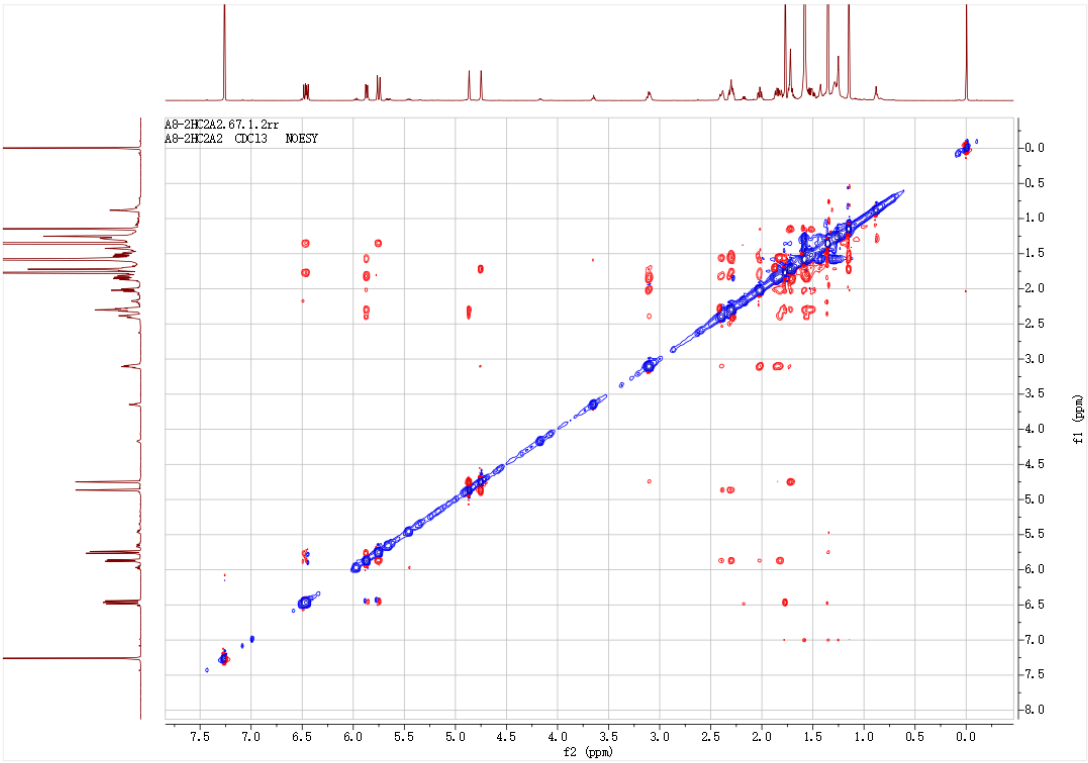

Figure S98. NOESY spectrum of **14** (600 MHz, $CDCl_3$)

D:\data\2021\EI202101755_A8-2HC2A2 -c1 10/11/2021 3:42:24 PM

EI202101755 A8-2HC2A2 -c1#11 RT: 2.07
T: + c EI Full ms [49.50-800.50]
m/z= 48-803

m/z	Intensity	Relative	Theo. Mass	Delta (mmu)	RDB equiv.	Composition
215.1774	2748265.0	22.35	215.1794	-2.05	5.5	$C_{16}H_{23}$
217.1586	843208.0	6.86	217.1587	-0.09	5.5	$C_{15}H_{21}O_{1}$
218.1642	316370.0	2.57	218.1665	-2.27	5.0	$C_{15}H_{22}O_{1}$
225.1641	1429262.0	11.62	225.1638	0.31	7.5	$C_{17}H_{21}$
226.1701	750871.0	6.11	226.1716	-1.51	7.0	$C_{17}H_{22}$
227.1793	1622788.0	13.20	227.1794	-0.15	6.5	$C_{17}H_{23}$
228.1868	6052541.0	49.22	228.1873	-0.48	6.0	$C_{17}H_{24}$
229.1587	621327.0	5.05	229.1587	-0.02	6.5	$C_{16}H_{21}O_{1}$
230.1654	554594.0	4.51	230.1665	-1.12	6.0	$C_{16}H_{22}O_{1}$
231.1738	837951.0	6.81	231.1743	-0.59	5.5	$C_{16}H_{23}O_{1}$
232.1801	288342.0	2.34	232.1822	-2.02	5.0	$C_{16}H_{24}O_{1}$
239.1790	399418.0	3.25	239.1794	-0.44	7.5	$C_{18}H_{23}$
240.1862	306836.0	2.50	240.1873	-1.09	7.0	$C_{18}H_{24}$
241.1586	317595.0	2.58	241.1587	-0.08	7.5	$C_{17}H_{21}O_{1}$
241.1948	353250.0	2.87	241.1951	-0.29	6.5	$C_{18}H_{25}$
243.1741	855220.0	6.96	243.1743	-0.23	6.5	$C_{17}H_{23}O_{1}$
243.2104	2031386.0	16.52	243.2107	-0.32	5.5	$C_{18}H_{27}$
244.1803	359733.0	2.93	244.1822	-1.83	6.0	$C_{17}H_{24}O_{1}$
245.1898	461657.0	3.75	245.1900	-0.21	5.5	$C_{17}H_{25}O_{1}$
246.1980	1533774.0	12.47	246.1978	0.14	5.0	$C_{17}H_{26}O_{1}$
253.1955	2744070.0	22.32	253.1951	0.43	7.5	$C_{19}H_{25}$
257.1899	333257.0	2.71	257.1900	-0.09	6.5	$C_{18}H_{25}O_{1}$
258.1976	279843.0	2.28	258.1978	-0.21	6.0	$C_{18}H_{26}O_{1}$
268.2187	1867822.0	15.19	268.2186	0.16	7.0	$C_{20}H_{28}$
269.2236	699282.0	5.69	269.2264	-2.74	6.5	$C_{20}H_{29}$
271.2057	3967903.0	32.27	271.2056	0.08	6.5	$C_{19}H_{27}O_{1}$
286.2289	8008206.0	65.13	286.2291	-0.23	6.0	$C_{20}H_{30}O_{1}$
289.2159	348483.0	2.83	289.2162	-0.35	5.5	$C_{19}H_{29}O_{2}$
304.2400	350254.0	2.85	304.2397	0.36	5.0	$C_{20}H_{32}O_{2}$

Figure S99. HREIMS spectrum of **14**

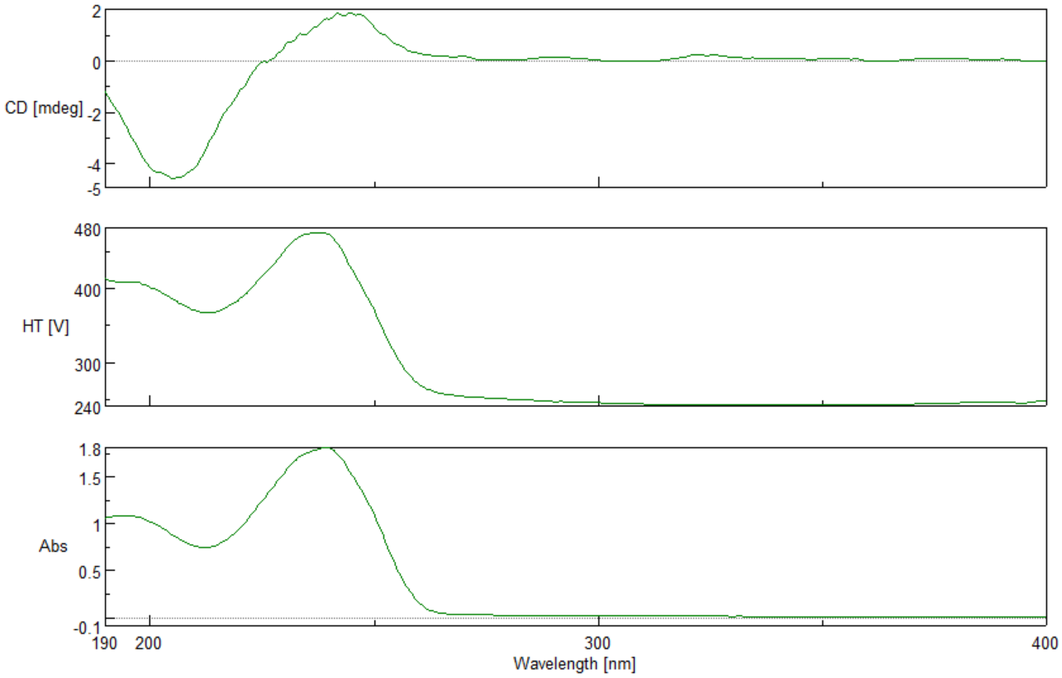

Figure S100. UV and CD spectrum of **14**

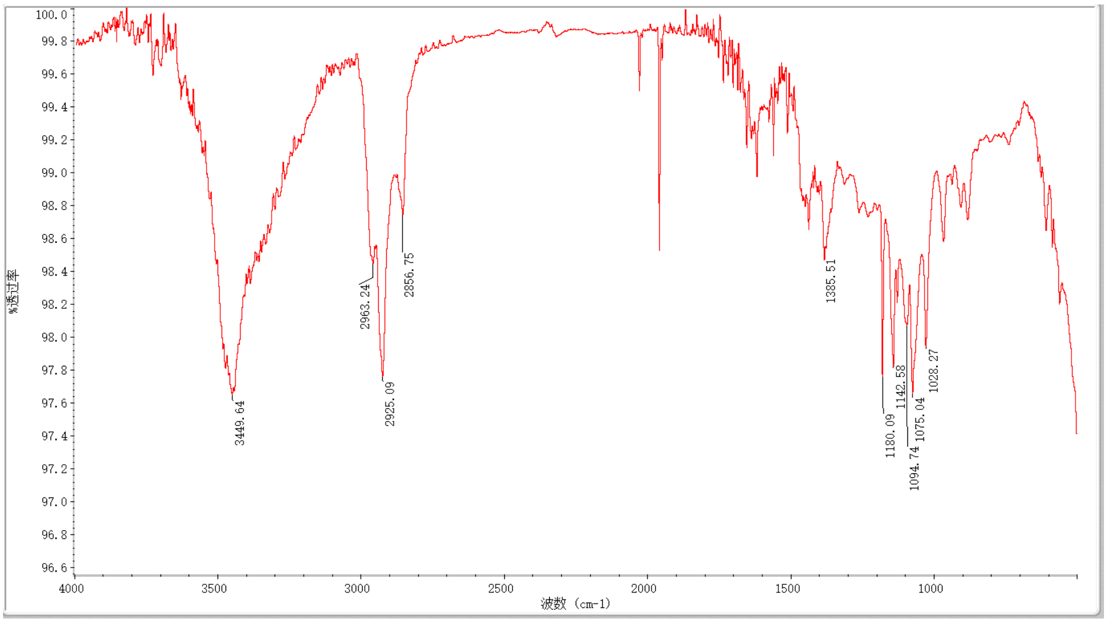

Figure S101. IR spectrum of **14**

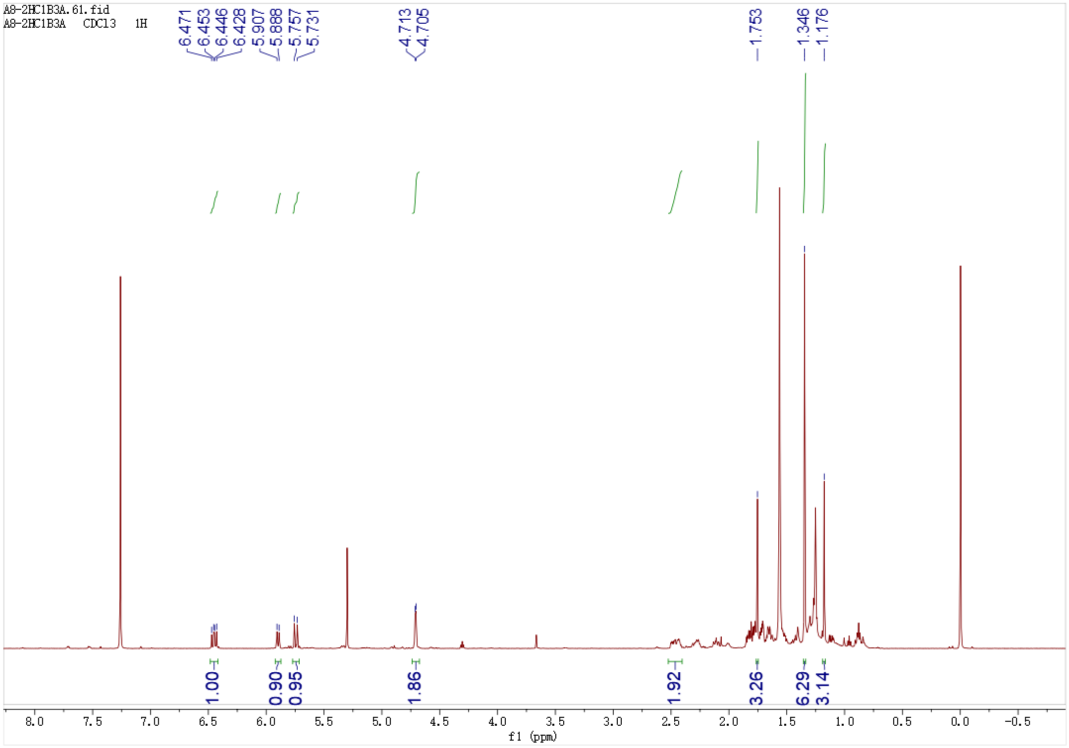

Figure S102. ^{1}H NMR spectrum of **15** (600MHz, $CDCl_3$)

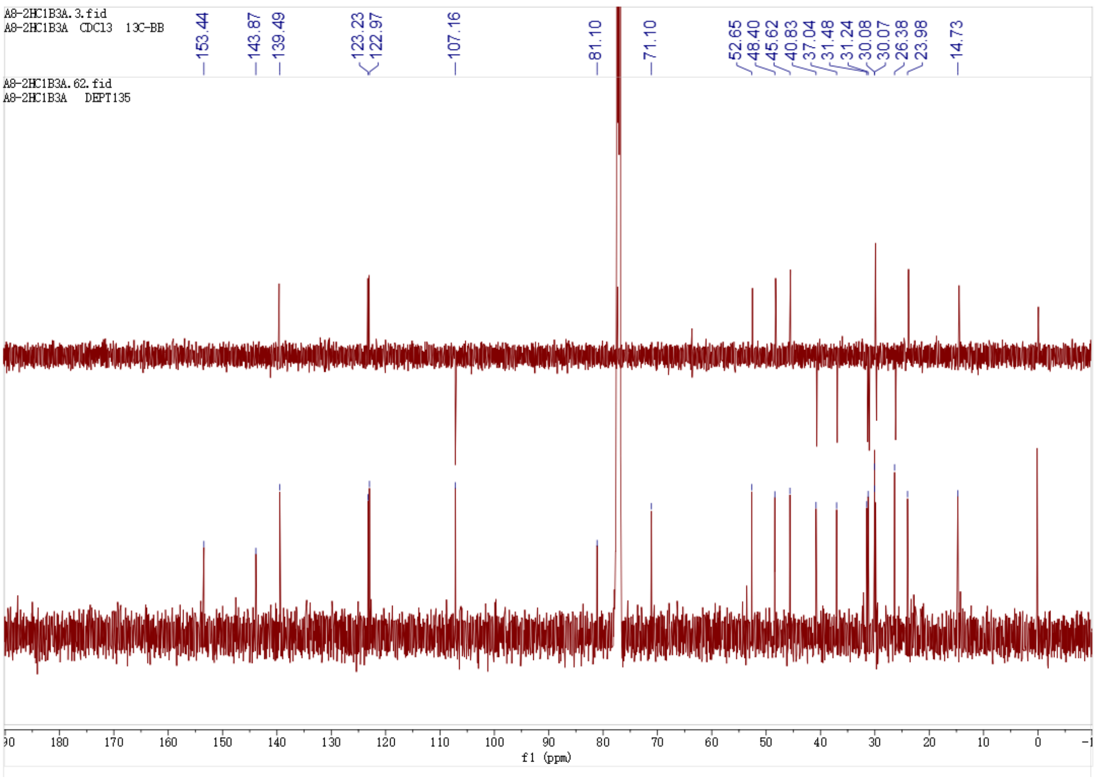

Figure S103. ^{13}C NMR spectrum of **15** (125 MHz, CDCl3)

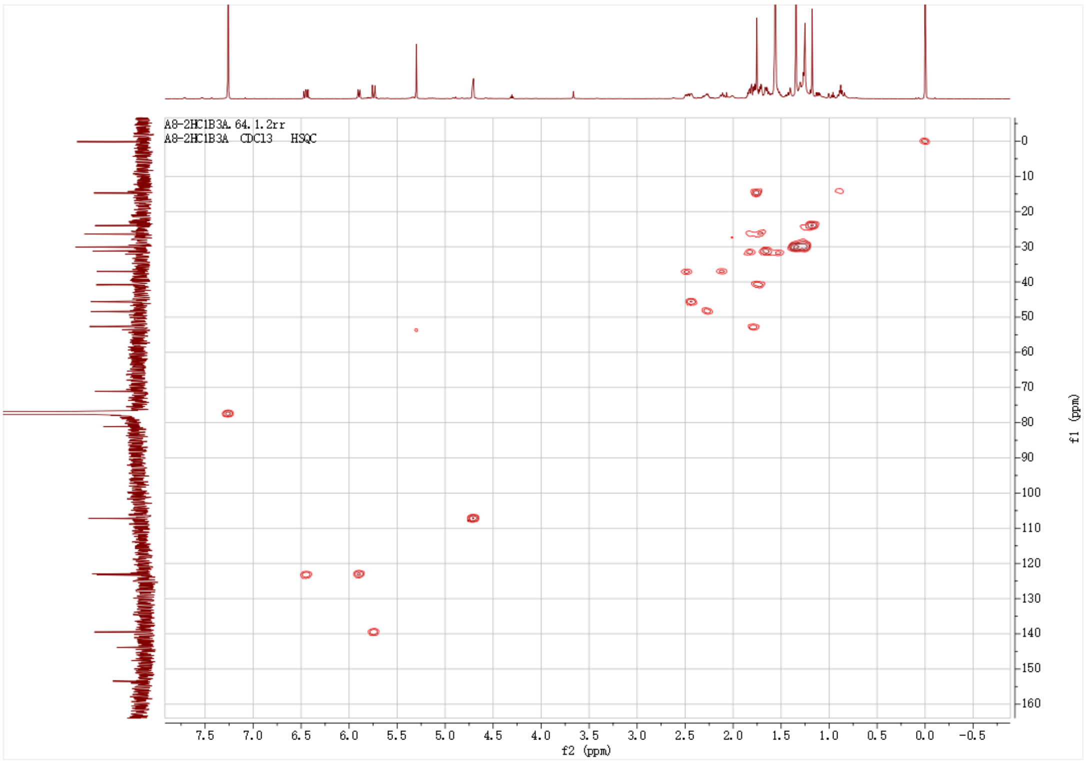

Figure S104. HSQC spectrum of **15** (600 MHz, CDCl3)

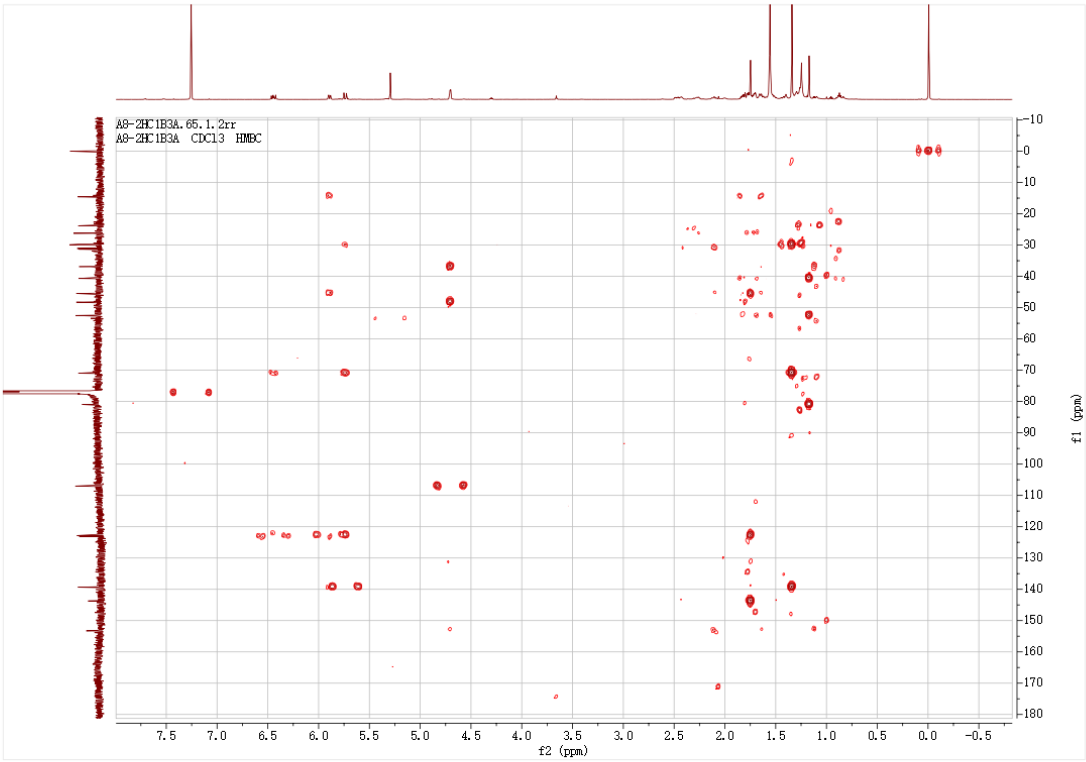

Figure S105. HMBC spectrum of **15** (600 MHz, $CDCl_3$)

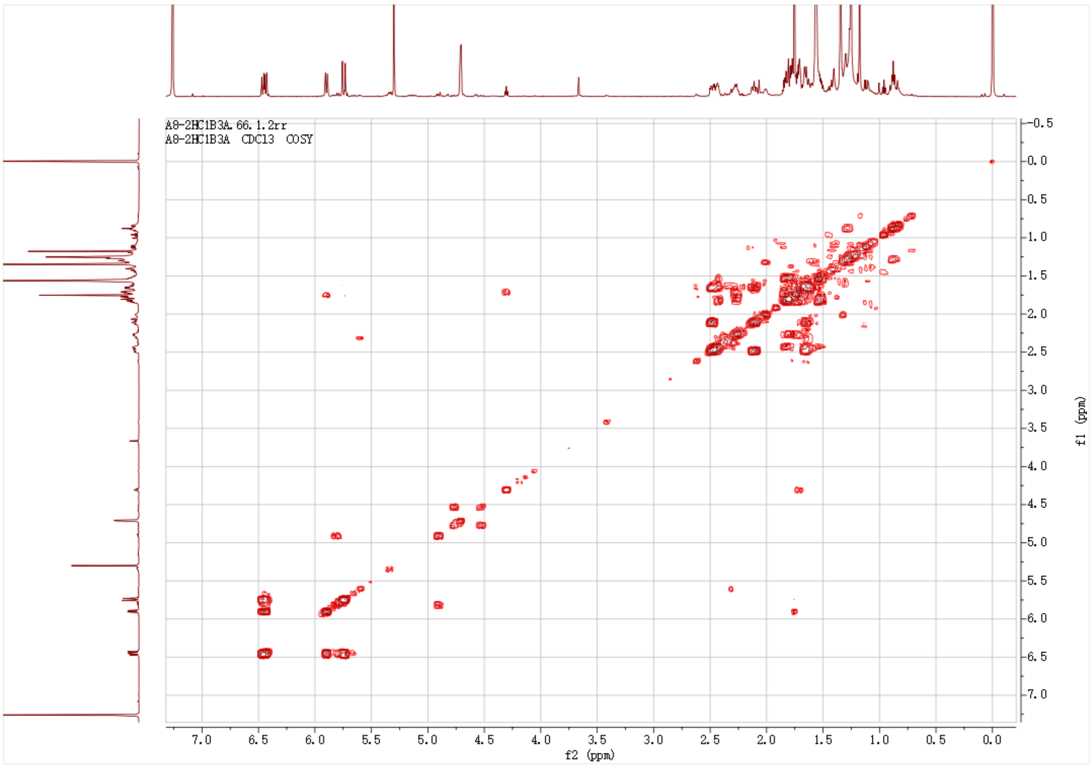

Figure S106. ^1H–^1H COSY spectrum of **15** (600 MHz, $CDCl_3$)

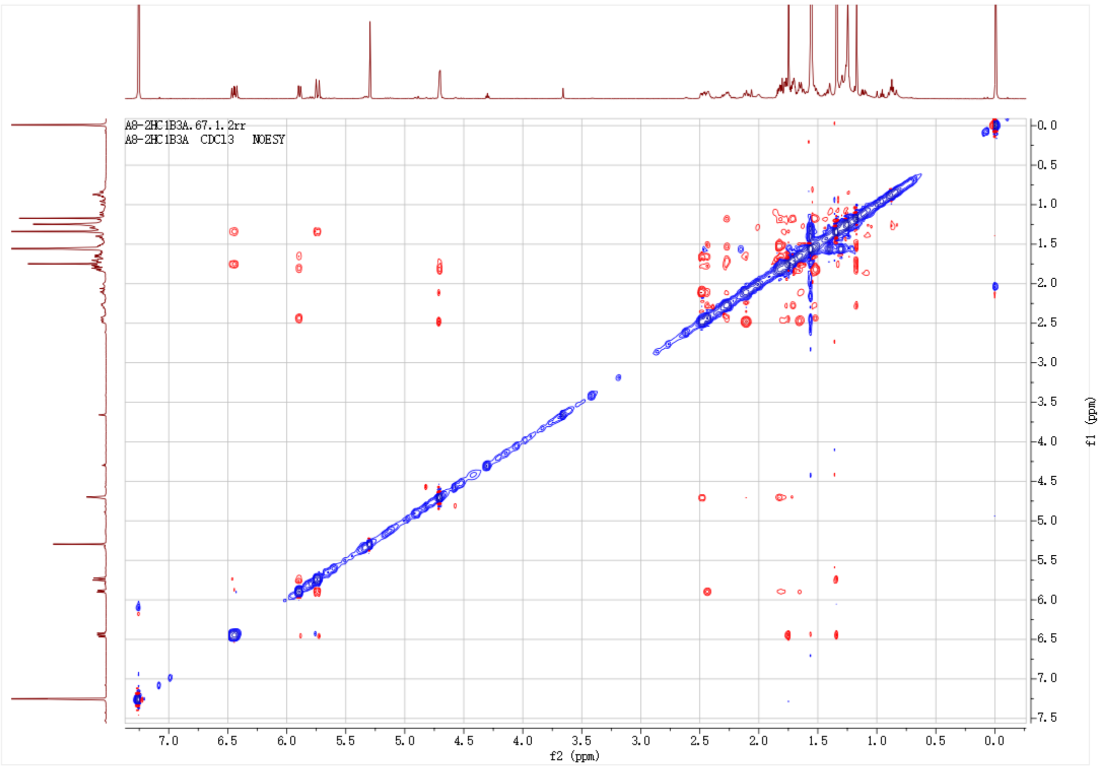

Figure S107. NOESY spectrum of **15** (600 MHz, $CDCl_3$)

D:\data\2021\EI202101813_A8-2HC1B3A -c1   10/21/2021 1:24:13 PM

EI202101813_A8-2HC1B3A -c1#7 RT: 1.24
T: + c EI Full ms [49.50-800.50]
m/z= 48-803

m/z	Intensity	Relative	Theo. Mass	Delta (mmu)	RDB equiv.	Composition
199.1479	3644199.0	31.50	199.1481	-0.23	6.5	$C_{15}H_{19}$
200.1538	1192140.0	10.30	200.1560	-2.18	6.0	$C_{15}H_{20}$
201.1635	2498025.0	21.59	201.1638	-0.23	5.5	$C_{15}H_{21}$
204.1504	1388662.0	12.00	204.1509	-0.44	5.0	$C_{14}H_{20}O_{1}$
211.1467	1077776.0	9.32	211.1481	-1.47	7.5	$C_{16}H_{19}$
213.1630	3543084.0	30.63	213.1638	-0.82	6.5	$C_{16}H_{21}$
214.1705	4530516.0	39.16	214.1716	-1.06	6.0	$C_{16}H_{22}$
215.1779	2846127.0	24.60	215.1794	-1.57	5.5	$C_{16}H_{23}$
225.1640	1082919.0	9.36	225.1638	0.24	7.5	$C_{17}H_{21}$
227.1796	1183173.0	10.23	227.1794	0.13	6.5	$C_{17}H_{23}$
228.1871	1855571.0	16.04	228.1873	-0.15	6.0	$C_{17}H_{24}$
243.1738	961042.0	8.31	243.1743	-0.59	6.5	$C_{17}H_{23}O_{1}$
243.2101	3286915.0	28.41	243.2107	-0.65	5.5	$C_{18}H_{27}$
253.1949	2327920.0	20.12	253.1951	-0.19	7.5	$C_{19}H_{25}$
268.2187	1472032.0	12.72	268.2186	0.14	7.0	$C_{20}H_{28}$
271.2054	4733635.0	40.92	271.2056	-0.21	6.5	$C_{19}H_{27}O_{1}$
286.2294	2905988.0	25.12	286.2291	0.28	6.0	$C_{20}H_{30}O_{1}$
304.2402	1077830.0	9.32	304.2397	0.53	5.0	$C_{20}H_{32}O_{2}$

Figure S108. HREIMS spectrum of **15**

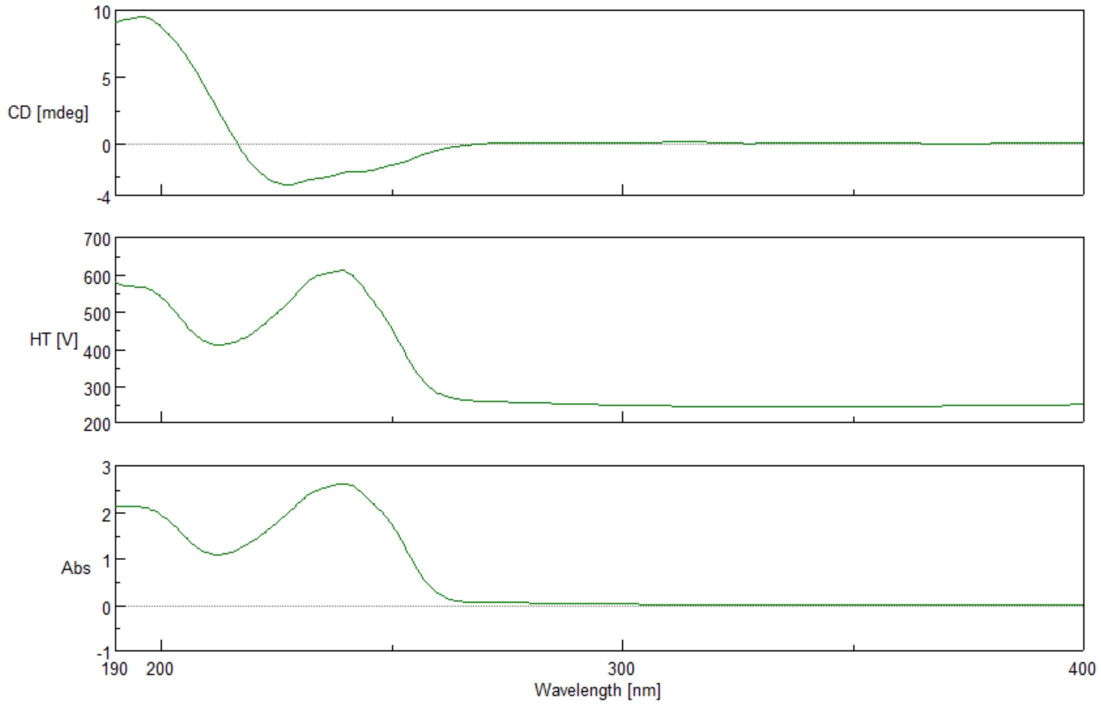

Figure S109. UV and CD spectrum of **15**

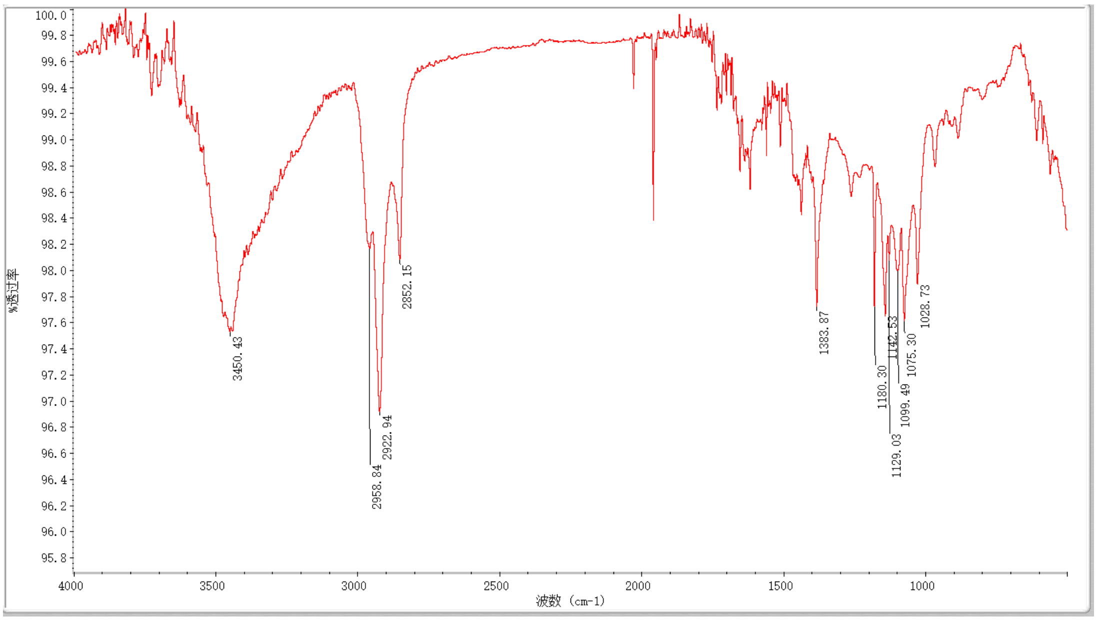

Figure S110. IR spectrum of **15**

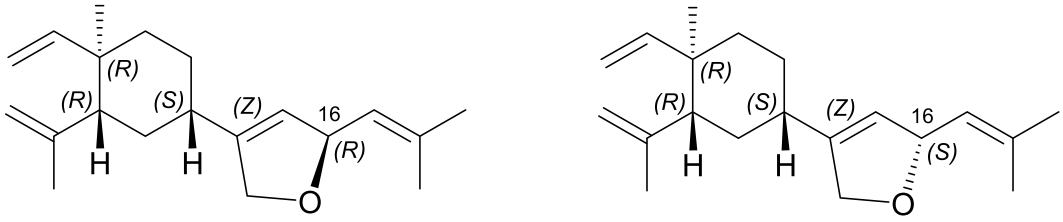

9a
isomer 1

9b
isomer 2

Figure S111. Structure of studied isomers of compound **9**

Functional		Solvent?		Basis Set		Type of Data	
mPW1PW91		PCM		6-31+G(d,p)		Shielding Tensors	
		DP4+	99.83%	0.17%	-	-	-
Nuclei	sp2?	xperimenta	Isomer 1	Isomer 2	Isomer 3	Isomer 4	Isomer 5
C		39.9	153.2	153.1			
C		52.6	143.1	143.0			
C	x	33.1	162.6	161.8			
C		37.2	156.5	156.7			
C	x	27.2	167.9	168.9			
C	x	39.7	160.3	160.2			
C		150.1	50.6	50.5			
C		147.5	50.6	50.6			
C	x	112.4	84.2	84.3			
C		24.9	169.2	169.1			
C	x	110.2	88.6	88.5			
C		16.7	176.44	176.64			
C		145.7	53.22	53.38			
C		120.9	75.26	75.37			
C	x	75.3	121.71	121.50			
C		83.1	111.39	111.22			
C		125.7	72.28	72.37			
C		135.5	62.77	62.64			
C		26	169.22	169.24			
C		18.2	177.55	177.55			
H		2.01	29.77	29.79			
H	x	1.61	29.83	30.09			
H	x	1.61	30.24	30.20			
H		2.1	29.50	29.54			
H	x	1.68	30.2202172	29.9820828			
H	x	1.44	30.0807476	30.1060126			
H	x	1.46	30.1707753	30.1569978			
H	x	1.46	30.0517074	30.0419154			
H		5.81	25.2314334	25.2541179			
H	x	4.58	26.3859087	26.4066554			
H	x	4.83	26.6772847	26.7058152			
H		1.71	30.0896834	30.1114021			
H		1.71	29.894635	29.9109793			
H		1.71	29.8363177	29.8413915			
H	x	4.9	26.4957979	26.4920136			
H	x	4.9	26.5262444	26.5121271			
H		1.01	31.3017912	31.2775949			
H		1.01	30.1543136	30.1677567			
H		1.01	30.5443112	30.5417124			
H		5.31	26.1803821	26.1739778			
H	x	4.54	26.8569364	27.1196528			
H	x	4.64	27.1185903	26.8802879			
H		5.5	26.1423184	26.1429093			
H		5.15	26.3244063	26.3122161			
H		1.73	30.0380179	30.041951			
H		1.73	30.0194962	30.174225			
H		1.73	30.1830762	30.0170812			
H		1.73	30.023883	30.3550914			
H		1.73	30.427398	29.4801701			
H		1.73	29.8146288	30.4130154			

Figure S112. Averaged isotropic magnetic shielding constants (σ) of studied isomers and experimental ^{1}H and ^{13}C data of **9**

Functional	Solvent?		Basis Set		Type of Data	
mPW1PW91	PCM		6-31+G(d,p)		Shielding Tensors	
	Isomer 1	Isomer 2	Isomer 3	Isomer 4	Isomer 5	Isomer 6
sDP4+ (H data)	42.51%	57.49%	–	–	–	–
sDP4+ (C data)	56.15%	43.85%	–	–	–	–
sDP4+ (all data)	48.63%	51.37%	–	–	–	–
uDP4+ (H data)	99.86%	0.14%	–	–	–	–
uDP4+ (C data)	46.34%	53.66%	–	–	–	–
uDP4+ (all data)	99.84%	0.16%	–	–	–	–
DP4+ (H data)	99.81%	0.19%	–	–	–	–
DP4+ (C data)	52.51%	47.49%	–	–	–	–
DP4+ (all data)	99.83%	0.17%	–	–	–	–

Figure S113. DP4+ results obtained using experimental ^{1}H and ^{13}C data of compound **9** versus isomers **9a** and **9b**

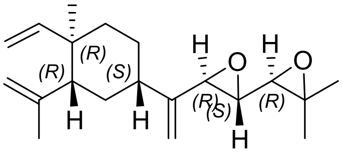

10a

isomer 1

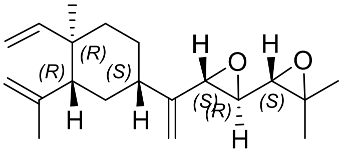

10b

isomer 2

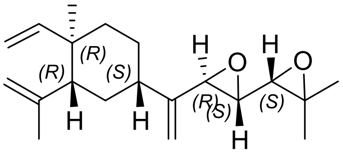

10c

isomer 3

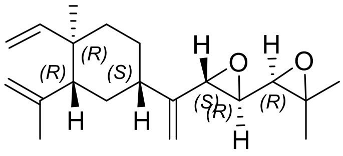

10d

isomer 4

Figure S114. Structure of studied isomers of compound **10**

Functional		Solvent?		Basis Set		Type of Data	
mPW1PW91		PCM		6-31+G(d,p)		Shielding Tensors	
		DP4+	0.00%	0.00%	0.00%	100.00%	–
Nuclei	sp2?	xperimenta	Isomer 1	Isomer 2	Isomer 3	Isomer 4	Isomer 5
C		39.8	153.2	153.2	153.0	153.3	
C		52.8	142.7	143.0	142.8	142.6	
C	x	33.9	161.3	161.7	160.8	161.5	
C		41.1	150.3	150.7	150.4	151.1	
C	x	27.1	168.7	168.6	169.2	167.7	
C	x	39.8	160.2	160.4	160.5	160.3	
C		150.1	50.6	50.7	50.5	50.6	
C		147.4	50.5	50.2	50.4	50.6	
C	x	110.3	88.5	88.5	88.6	88.7	
C	x	112.4	84.3	84.6	84.4	84.3	
C		16.7	176.1	176.6	176.5	176.2	
C		25	169.26	168.40	168.31	169.14	
C		149.2	48.95	49.01	48.77	48.48	
C	x	109.4	88.10	88.55	88.69	88.13	
C		56	140.54	140.28	139.04	138.60	
C		58.5	134.21	133.95	135.71	135.28	
C		63	132.43	132.44	134.01	134.51	
C		58.6	137.30	137.33	136.13	136.21	
C		24.7	171.51	171.52	171.89	171.77	
C		19.7	176.09	176.09	176.60	176.57	

H		2.02	29.78	29.80	29.83	29.81
H	x	1.6	29.98	29.81	29.92	29.81
H	x	1.53	30.16	30.26	30.18	30.27
H		1.99	29.5340503	29.517696	29.5956355	29.6243786
H	x	1.62	29.7831772	29.9978599	29.7877729	29.9591575
H	x	1.5	30.2143417	30.0858343	30.1919774	30.0895766
H	x	1.48	30.1046551	30.1281622	30.1176191	30.1096067
H	x	1.48	29.9937712	30.0083197	29.9991686	30.0194794
H		5.81	25.2271971	25.2583384	25.2592416	25.2241271
H	x	4.9	26.4846061	26.4854008	26.4922313	26.4890081
H	x	4.9	26.4944741	26.5012281	26.5043046	26.5027924
H	x	4.83	26.3857173	26.3608126	26.3667446	26.3900452
H	x	4.57	26.6718796	26.6631036	26.6368418	26.673243
H		1.01	31.2459623	29.8978105	29.8906625	31.2651856
H		1.01	30.1154923	29.8163244	29.818158	30.0740645
H		1.01	30.5278597	30.0877662	30.092478	30.528799
H		1.71	30.0889904	31.2842685	31.2837359	30.086878
H		1.71	29.8894967	30.145295	30.0930534	29.8766822
H		1.71	29.838201	30.4981115	30.4880025	29.8909581
H	x	5.09	26.392703	26.3902534	26.4149611	26.3753123
H	x	4.95	26.5050457	26.5294215	26.5164921	26.4797489
H		3.31	28.4239683	28.4298219	28.2685267	28.2544793
H		2.73	29.3437228	29.3441615	29.0729206	28.9523859
H		2.63	29.4835126	29.4809384	29.0557675	28.9546145
H		1.35	30.2351614	30.24241	30.2165028	30.1538297
H		1.35	31.0853627	30.161786	30.1429085	30.2238291
H		1.35	30.1598604	31.091115	31.0473772	31.0521698
H		1.39	30.1808229	30.3462962	30.2116177	30.3701416
H		1.39	30.8349274	30.8392735	30.3502838	30.2137848
H		1.39	30.3443156	30.1821691	30.7772859	30.7714605

Figure S115. Averaged isotropic magnetic shielding constants (σ) of studied isomers and experimental ^{1}H and ^{13}C data of **10**

Functional	Solvent?		Basis Set		Type of Data	
mP[illegible]1P[illegible]91	PC[illegible]		6-31+G(d,p)		Shielding Tensors	
	Isomer 1	Isomer 2	Isomer 3	Isomer 4	Isomer 5	Isomer 6
sDP4+ (H data)	0.01%	0.00%	0.00%	99.99%	-	-
sDP4+ (C data)	45.05%	19.28%	6.69%	28.98%	-	-
sDP4+ (all data)	0.01%	0.00%	0.00%	99.99%	-	-
uDP4+ (H data)	0.03%	0.00%	0.00%	99.97%	-	-
uDP4+ (C data)	15.75%	27.27%	20.23%	36.76%	-	-
uDP4+ (all data)	0.01%	0.00%	0.00%	99.99%	-	-
DP4+ (H data)	0.00%	0.00%	0.00%	100.00%	-	-
DP4+ (C data)	29.13%	21.59%	5.55%	43.73%	-	-
DP4+ (all data)	0.00%	0.00%	0.00%	100.00%	-	-

Figure S116. DP4+ results obtained using experimental ^{1}H and ^{13}C data of compound **10** versus isomers **10a**, **10b, 10c, 10d**
